# Phylogeny in Aid of the Present and Novel Microbial Lineages: Diversity in *Bacillus*

**DOI:** 10.1371/journal.pone.0004438

**Published:** 2009-02-12

**Authors:** Shalini Porwal, Sadhana Lal, Simrita Cheema, Vipin Chandra Kalia

**Affiliations:** 1 Microbial Biotechnology and Genomics, Institute of Genomics and Integrative Biology (IGIB), CSIR, Delhi University Campus, Delhi, India; 2 Department of Biotechnology, University of Pune, Pune, India; University of California, Berkeley, United States of America

## Abstract

*Bacillus* represents microbes of high economic, medical and biodefense importance. *Bacillus* strain identification based on 16S rRNA sequence analyses is invariably limited to species level. Secondly, certain discrepancies exist in the segregation of *Bacillus subtilis* strains. In the RDP/NCBI databases, out of a total of 2611 individual 16S rDNA sequences belonging to the 175 different species of the genus *Bacillus*, only 1586 have been identified up to species level. 16S rRNA sequences of *Bacillus anthracis* (153 strains), *B. cereus* (211 strains), *B. thuringiensis* (108 strains), *B. subtilis* (271 strains), *B. licheniformis* (131 strains), *B. pumilus* (83 strains), *B. megaterium* (47 strains), *B. sphaericus* (42 strains), *B. clausii* (39 strains) and *B. halodurans* (36 strains) were considered for generating species-specific framework and probes as tools for their rapid identification. Phylogenetic segregation of 1121, 16S rDNA sequences of 10 different *Bacillus* species in to 89 clusters enabled us to develop a phylogenetic frame work of 34 representative sequences. Using this phylogenetic framework, 305 out of 1025, 16S rDNA sequences presently classified as *Bacillus* sp. could be identified up to species level. This identification was supported by 20 to 30 nucleotides long signature sequences and *in silico* restriction enzyme analysis specific to the 10 *Bacillus* species. This integrated approach resulted in identifying around 30% of *Bacillus* sp. up to species level and revealed that *B. subtilis* strains can be segregated into two phylogenetically distinct groups, such that one of them may be renamed.

## Introduction

Phylogenetics, the science of estimating the evolutionary past is based on the comparison of DNA or protein sequences [1]. In this age of rapid and rampant gene sequencing, the availability of a large amount of genomic information from 639 sequenced genomes (http://www.ncbi.nlm.nih.gov) and 16S rDNA sequencing data of 451545 isolates (http://rdp.cme.msu.edu/) has given new dimensions to microbial taxonomy and is likely to lead to revision of concepts such as species, organism and evolution [2]. 16S rDNA gene sequencing is often used as an alternative method to define microbes at species level. Protein coding genes having high variability has been successfully used to differentiate taxa that cannot be identified solely on the basis of 16S rDNA sequences e.g., heat shock protein (*hsp65*) [3], *hsp70*, ATPase-ß-subunit, RNA polymerases and recombinase (*recA*) [4]. In addition, partial *rpoB* sequences have been applied to classify members of the genus *Mycobacterium* [3], [5]; *gyrB* gene sequences have been used to define *Acinetobacter* members [6]; *Mycobacterium* [7]; *Pseudomonas* [8] and *Shewanella* [9], *gyrA* gene for defining *Bacillus subtilis* and related taxa [10].

Members of the genus *Bacillus* comprise gram-positive, spore-forming, rod-shaped, aerobic bacteria. *Bacillus* species are phenotypically and genotypically heterogenous [11], [12]. *Bacillus* represents microbes of high economic, medical and biodefense importance such as bio-pesticides [13] and biofuels [14]–[16], pathogens [17], [18]. *Bacillus thuringiensis* is currently used for the biological control of insects and in crop protection [19]. *B. subtilis* strains produce a broad spectrum of bioactive peptides with great potential for biotechnological and biopharmaceutical applications [20]. *Bacillus licheniformis* strains also produce a variety of peptide antibiotics such as bacitracin [21], [22], bacteriocin [23] and are also known to contaminate industrial processes [24]–[26] and cause food poisoning [27], [28]. *Bacillus* spores are being used as human and animal probiotics despite the fact that studies now indicate extensive mislabeling of constituent *Bacillus* strains [29], [30]. Therefore, it is becoming increasingly clear that a more rigorous selection process is required for *Bacillus* probiotic candidates [31], [32]. Because of these divergent characteristics, questions arise concerning intra species diversity that could differentiate isolates of potential economic importance. It is for these reasons that 11 closely related *Bacillus* are among the 29 Bacillales sequenced so far (http://www.ncbi.nlm.nih.gov).

### Bacterial Systematics

Bacterial systematics began long before the discovery of DNA as the heritable material. Bacteria were originally classified largely on the basis of phenotype, morphology, ecology and other associated metabolic characteristics. Bacterial taxonomy has been a tedious, esoteric and uncertain discipline. Some excitement was brought in by the team led by Dr. Carl Woese. They provided a detailed insight into bacterial phylogeny by exploiting molecular biology in an innovative manner [33]. Genomic discoveries are posing a challenge to the classical bacterial systematics [34].

The most common molecular ecological techniques applied to assess the bacterial diversity and to analyze the genetic relationships between *Bacillus* species are based on genome fingerprinting [34]; DNA-DNA re-association studies [35], [36]. Rep-PCR has been shown to be a useful technique in the subtyping of *Bacillus* species [37], [38]. On the other hand, protein coding genes such as *gyrA* and *rpoB* exhibit much higher genetic variation and have been used for the classification of closely related taxa within the *B. subtilis* group [10], [39]. The highly discriminating power of these genes has also been used to assess intraspecies diversity of *B. licheniformis* [40]. In general, 16S rDNA sequences are used in bacterial classification as a frame work for species delineation e.g., in *Bacillus* [41]. The genetic relationship and the phylogeny among organisms are analysed on the basis of molecular chronometer, the ribosomal operon, especially the 16S and 23S rDNA genes [42], [43]. Since these genes are much conserved among microbes, they cannot be used to unambiguously discriminate at the species level [44]. With respect to the ribosomal genes, the internal transcribed spacers (ITS) between the 16S and 23S rDNA genes are hypervariable and can display polymorphisms especially in regions not implicated in rDNA maturation [43]–[48]. Several species resembling *Bacillus* species have been described but these new species cannot be phenotypically differentiated from the bona fide members.

Recent advances in the areas of genomics and proteomics have provided excellent opportunities to exploit genome sequence databases. Since the beginning of the genomic era the phylogenetic relationships among bacteria has been done on the basis of 16S rDNA gene Restriction Fragment Length Polymorphism (RFLP), such as for *Streptococcus aeruginosa* and *S. adjacens* [49]; *Pseudomonas aeruginosa* [50]; *Borrelia* [51]; *Lactobacillus* [52]; *Helicobacter* [53] and *Bacillus* [54]. Other structural genes that are highly conserved between species and which are useful to infer phylogenesis are the tRNA genes [55], [56]. Consensus tRNA gene primers generally produce species-specific patterns, which have been successfully used to discriminate species belonging to the same genus as reported for *Acinetobacter* sp. [57]; *Streptococcus* sp. [58]; *Staphylococcus* sp. [57] and *Bacillus* sp. [59]–[61].

The Ribosomal Data Project (RDP-II) provides the research community with aligned and annotated 16S rDNA gene sequences, and a phylogenetically consistent taxonomic framework for these data. The RDPII database has 451545, 16S rDNA sequences as on Nov 2007 (http:// rdp.cme.msu.edu).

Species demarcation of some *Bacillus* isolates is quite complex, since many isolates don't provide characteristic reactions that are considered typical of that species [62], [63]. Although diversity identification is based on the use of a range of different morpho-physiological and chemical parameters as well as genetic analysis to tentatively identify the isolates to the species level. For example, the API system only allows the identification of 19 of the best identified and most common species and therefore fails to identify a number of isolates [31].

The various studies have emphasized the need to reclassify certain species. Ash et al., [42] analysed 51 species of *Bacillus* and found that they fell into five distinct grouping based on 16S rDNA sequences, which they believed, “clearly represent separate genera”. A few of the sporulating aerobes proved to be sequenced outliers, which might represent the nuclei of other hitherto unrecognized genera. This marked the beginning of a new era in the reorganization for the genus *Bacillus*. It is so far a continuous process, since several new genera have been defined from organisms previously known as *Bacillus* [64]: *Alicyclobacillus*, *Aneurinibacillus*, *Brevibacillus*, *Geobacillus*, *Gracibacillus*, *Paenibacillus*, *Salibacillus*, *Ureibacillus* and *Virgibacillus*. Out of the 41 *Bacillus* species reported by Joung and Coté [54], *B. kaustophilus*, *B. sterothermophilus*, *B. thermoglucosidasius* and *B. thermoleovorans* have been transferred to the newly created genus *Geobacillus* [65]. Certain others such as *Bacillus globisporus*, *B. pasteurii* and *B. psychrophilus* have been reclassified to the genus *Sporosarcina* [66]. Similarly, *Bacillus marinus* has been reclassified as *Marinibacillus marinus* [67].

### Defining Diversity of *Bacillus*

#### *Bacillus cereus* group

Three species of the *Bacillus cereus* group (*B. cereus*, *Bacillus anthracis* and *B. thuringiensis*) have a marked impact on human activity. *B. cereus* and *B. anthracis* are important pathogens of mammals, including humans, and *B. thuringiensis* is extensively used in the biological control of insects [68]. In *B. cereus* group the chromosomes of the sequenced members are extremely similar. The number of genes unique to one species is quite limited and often represents metabolic adaptations [34]. Although *B. anthracis* can be distinguished from *B. cereus* on the basis of biochemical tests [69], however, certain isolates on the periphery such as the pathogenic *B. cereus* G9241 [70] cannot be properly classified unless it is checked for the plasmid pX01, which is native to *B. anthracis* [34]. Since comparisons of chromosomal contents are not able to easily distinguish different members of *B. cereus* group - *B. cereus*, *B. anthracis* and *B. thuringiensis*, one may have to look for some more easily recognizable markers. Based on the phylogenetic homogeneity, 86 strains of *B. thuringiensis* could be closely clustered together in four different groups (Bt group I-IV) at a DNA similarity rate of 93% [54]. *B. thuringiensis* is closely related to *B. anthracis* and *Bacillus mycoides* and is regarded as a subvariant of *B. cereus* based on genotypic data [12], [71], [72]. Demonstration of the high genetic relatedness of *B. thuringiensis*, *B. anthracis* and *B. cereus* has led to the suggestion, that these are members of a single species of *B. cereus sensu lato* [48], [72], [73]. Overall genetic studies have shown that *B. cereus* and *B. thuringiensis* are essentially identical [74]. *B. anthracis* can be distinguished from *B. cereus* and *B. thuringiensis* through microbiological and biochemical tests. *B. anthracis* isolates are non-hemolytic, non-motile, penicillin sensitive, susceptible to γ-phage and produce a poly-γ-D-glutamic acid capsule [70]. The classification and taxonomic separation of members of *B. cereus* group is rather difficult even with modern molecular tools.

Analysis of large culture collections of *B. cereus*, *B. anthracis* and *B. thuringiensis* by AFLP and MLST [70], [75], [76] have identified a class of organisms containing toxigenic *B. cereus* and *B. thuringiensis* that are closely related to *B. anthracis*. These isolates were phylogenetically distinct from environmental *B. cereus* and *B. thuringiensis* [75] and might represent the closest ancestor *B. anthracis*.

#### *Bacillus subtilis* group

Although several species resembling *B. subtilis* have been described over the last two decades, identification of *B. subtilis* like organisms has been quite difficult and laborious. Presently, the difficult part lies in confirming the significance of the sequence clusters. Such organisms have almost identical 16S rDNA sequences (99.2 to 99.6% sequence similarity) [42], [77]. Comparative sequence analysis for the *gyrA* gene, which codes for DNA gyrase subunit A of 7 representatives of *B. subtilis* and allied taxa provided a frame work for their rapid and accurate classification and identification [10], [65] divided *B. subtilis* in to two subspecies, namely *B. subtilis* subsp. *subtilis* and *B. subtilis* subsp. *spizizenii* on the basis of cell wall chemistry and DNA-DNA relatedness data.

#### *Bacillus licheniformis*

The intra-specific diversity of *B. licheniformis* studied by means of MLEE and phenotypic analysis could distinguish these isolates into two main subgroups [78]. Despite the high phenotypic similarities among the 182 isolates of *B. licheniformis*, the DNA-DNA reassociation studies showed three very distinct groups. These were therefore regarded by Manachini et al., [79] as genomovars of *B. licheniformis*. A 5′ hypervariable region of the 16S rDNA corresponding to *B. subtilis* at positions 41–307 and similarly a *B. licheniformis* specific Taq probe 5′- FAM-GAG CTT GCT CCC TTA GGT CAG – Dab Syl – 3′ were designed for targeting a section of this region corresponding to *B. subtilis* 16S rDNA numbering positions 79–99.

The *B. licheniformis* chromosome contains large regions that are co-linear with the genomes of *B. subtilis* and *Bacillus halodurans* and approximately 80% of the predicted *B. licheniformis* coding sequences have *B. subtilis* orthologs [80]. Recent taxonomic studies indicate that *B. licheniformis* is closely related to *Bacillus amyloliquefaciens* and *B. subtilis* on the basis of comparisons of 16S rDNA and 16S–23S ITS nucleotide sequence Lapidus et al., [81] and Xu and Cote [82] recently constructed a physical map of the *B. licheniformis* chromosome using a PCR approach and established a number of regions of co-linearity where gene content and organization were conserved with the *B. subtilis* genome. The close relationship between *B. licheniformis* and *B. halodurans* compared to *B. subtilis* has been shown on the basis of i) replication terminator protein (*rtp*), which is lacking in *B. licheniformis* [80] and *B. halodurans* [83]; ii) putative transposase of *B. licheniformis* shows close relation to *B. halodurans* [80], [83]; iii) the 27 predicted extracellular proteins encoded by *B. licheniformis* ATCC 14580 genome that are not found in *B. subtilis* 168 [80]; iv) two gene clusters involved in cellulose degradation and utilization were discovered in *B. licheniformis* and there are no counterparts in *B. subtilis* 168. Sixty six per cent of the predicted *B. licheniformis* genes have orthologs in *B. subtilis* and 55% of the genes models are represented by orthologous sequences in *B. halodurans*, 1719 orthologs are common to all these three species. These conservations clearly support previous hypothesis [82] that *B. subtilis* and *B. licheniformis* are phylogenetically and evolutionarily closer to each other than to *B. halodurans* [80]. In our study, *B. halodurans* reference strains were very distinct from *B. licheniformis* and *B. subtilis*.

#### *Bacillus halodurans*

*B. halodurans* is a group of rod shaped gram positive, aerobic or anaerobic bacterium. An alkaliphilic bacterium, strain C-125 (JCM9153), isolated in 1975, and was reidentified as *B. halodurans* based on 16S rDNA sequence DNA-DNA hybridization analysis. Out of 11 factors which belong to the extracytoplasmic function family, 10 are unique to *B. halodurans*. One hundred and twelve CDSs in *B. halodurans* genome showed significant similarity to the transposases or recombinases from various species such as *Anabeana* sp., *Rhodobacter capsulatus*, *Lactococcus lactis*, *Enterococcus faecium*, *Clostridium beijerinckii*, *Staphylococcus aureus* and *Yersinia pseudotuberculosis* indicating that these have played an important evolutionary role in HGT and also in internal rearrangement of the genome [83].

*B. halodurans* and *B. subtilis* similarities: genome sequence comparisons between *B. halodurans* and *B. subtilis* reveal that among the total CDSs; 8.8% match sequences of proteins found only in *B. subtilis*. The Shine-Dalgarno (SD) sequence was complementary to the one found at the 3′ end of 16S rDNA (UCU UUC UCC ACU AG…) of alkaliphilic *B. halodurans* C-125 [83]; is the same as that of *B. subtilis*. *B. halodurans* C-125 is quite similar to *B. subtilis* in terms of genome size, G+C content of the genomic DNA and the physiological properties used for taxonomical identification, except the alkaliphilic phenotype [84]. Also, the phylogenetic placement of *B. halodurans* C-125 based on 16S rDNA sequence analysis indicates that this organism is more closely related to *B. subtilis* than to other members of the genus *Bacillus*. Four types of ATPases were also well conserved between *B. halodurans* and *B. subtilis*. ABC transporter genes are the most frequent class of protein coding genes found in *B. halodurans* genomes as an in the case of *B. subtilis*.

#### *Bacillus pumilus*

*Bacillus pumilus* is commonly isolated from a variety of environmental sources, particularly feaces of animals. *B. pumilus* grows as a smooth colony that becomes yellow with increased incubation; the organism is motile, b-hemolytic on blood agar, catalase positive, salt tolerant and penicillin susceptible and does not grow under strict anaerobic conditions [85]. *B. pumilus* has toxic properties; it has cytopathic effects in vero cells, hemolytic activity, lecithinase production, and proteolytic action on casein. Recently, From et al. [86] detected an emetic toxin that can be related to food poisoning incidents. Human infection due to *B. pumilus* is exceptional.

#### *Bacillus megaterium*

*Bacillus megaterium* is a gram-positive, mainly aerobic spore forming bacterium found in widely diverse habitats from soil to seawater, sediment, rice paddies, honey, fish and dried food. *B. megaterium* has been industrially employed for more than 50 years, as it possesses some very useful and unusual enzymes and a high capacity for the production of exoenzymes. Genetic tools for this species include transducing phages and several hundred mutants covering the processes of biosynthesis, catabolism, division, sporulation, germination, antibiotic resistance, and recombination [87].

#### *Bacillus sphaericus*

*Bacillus sphaericus* is an aerobic, mesophilic, spore-forming bacterium with terminal swollen sporangia and spherical spores [88]. Strains of *B. sphaericus* are toxic towards mosquito larvae and can be used as biological control agents of the important vectors of filariasis, malaria and yellow fever [89]. Most studies interested in the use of these highly toxic strains in biocontrol programmes such as in Brazil have focused on the isolation of more adapted strains in tropical conditions [90]. Strains of *B. sphaericus* were divided in to five distinct groups and group II was formed by two subgroups, IIA and IIB. All toxic strains were located in DNA homology subgroup IIA [91] but this homology group also contained non-pathogens [92]. Different techniques such as phage typing [93], serotyping [94], [95], cellular fatty acid analysis [96] and MLEE on agarose gel [97] were also used in order to identify entomopathogenic *B. sphaericus*. However, only a few works report the diversity within mosquito toxic *B. sphaericus* strains [98], [99]. As result, the attempt to define a new species (DNA homology group IIA) based on mosquito pathogenicity as the unique characteristic was then discarded [92]. Subsequently, using cloned toxin genes *bin* and *mtx* from *B. sphaericus* as probes resulted in segregating 30 strains into 22 groups within the DNA homology group IIA [37]. From the variation in the number and size of bands it was possible to identify similarities among the strains resulting in 5 groups in BOX-PCR and 8 groups in REP-PCR. *B. sphaericus* strains isolated from diverse habitats and geographically different locations in Brazil, were phenotypically and genetically quite heterogeneous and can be potentially useful as biological control agents against mosquito larvae [37].

Although the current classification of species within the genus *Bacillus* and correlated genera is well established and is based on a combination of numerous approaches. The present study aims at determining whether or not a new set of parameters be deduced to develop a method, which could be informative enough to be useful for the more effective classification of *Bacillus* species and other microbes. Since 16S rDNA sequence repository is quite large, we focused our studies on this gene.

The genus *Bacillus* contain a heterogeneous assembly of aerobic or facultative anaerobic bacteria, widely distributed in the environment. The phenotypic protocols though important need a supplementation of molecular approaches. Molecular approaches based on DNA sequence minimize problems associated with typability and reproducibility and enables assembly of large reference databases [100]. Sequence specific primers for 16S rDNA gene have proved to be gold standards for the identification of pure cultures of *Bacillus* sp. such a *B. subtilis* [101], *B. cereus* and *B. thuringiensis* [102], *Paenibacillus alvei* (formerly *Bacillus alvei*) [103]. Genus specific primer have been successfully developed for *Lactobacillus*, *Mycoplasma*, *Bifidobacterium*, *Pandorea*, *Clostridium* and recently for certain *Bacillus* strains [104].

## Results

During the investigation, 1121 individual 16S rDNA sequences belonging to the genus *Bacillus* (from RDP/NCBI sites: http://rdp.cme.msu.edu/; http://www.ncbi.nlm.nih.gov/) were analyzed for generating species-specific framework and probes as a rapid tool for their identification. These sequences represented a total of 10 different species. *Bacillus cereus* group comprising of *B. anthracis*, *B. cereus* and *B. thuringiensis* was represented by 472 strains i.e. 21.9% of the total 16S rDNA sequences collected and analysed here in this study. A few other species, for which a fairly large number of 16S rDNA sequences are available, belong to *B. subtilis* (271 strains), *B. licheniformis* (131 strains), *B. pumilus* (83 strains), *B. megaterium* (47 strains), *B. sphaericus* (42 strains), *B. clausii* (39 strains) and *B. halodurans* (36 strains). These later seven species constituted 30.24% (649/2146) of the total sequences studied (Table 1).

**Table 1:** 16S rDNA sequences of *Bacillus* species and number of sequences used in this study (http://rdp.cme.msu.edu/).

S. No.	Organism	No. of sequences
1.	*Bacillus anthracis*	153
2.	*B. cereus*	211
3.	*B. thuringiensis*	108
4.	*B. subtilis*	271
5.	*B. licheniformis*	131
6.	*B. pumilus*	83
7.	*B. megaterium*	47
8.	*B. sphaericus*	42
9.	*B. clausii*	39
10.	*B. halodurans*	36
11.	*B.* sp.	1025
	Total	2146

### Phylogeny of *Bacillus* species

#### *Bacillus cereus* group

**(i)** ***B. anthracis***: Phylogenetic tree based on the 16S rDNA sequences from 153 strains of *B. anthracis* revealed 6 clusters (BAI to BAVI) (Figure S1). These different clusters were represented by 5 to 87 strains. Twelve strains could not be segregated clearly into any of these clusters. All the sequences from each of the cluster when subjected to multiple alignments showed that the strains were fairly similar over a large region. A visual scan of the profiles in each of the clusters (BAI to BAVI), showed that the level of genetic variability is quite low, since 84.2% of the total sequence i.e. 1309/1554 nucleotides (nts) were deemed to be indistinguishable. Further a comparison of the total length of each of the groups support the limited genetic diversity between cluster BAI - BAIV on one hand and BAV - BAVI on the other. These clusters possess nearly identical similarity rate and were considered to be identical. Hence, the 153 *B. anthracis* 16S rDNA sequences were in fact belonging to 4 representative groups varying in length from 1309 nts to 1554 nts. The variability among four clusters was found to extend on either side of the core region, 78 nts upstream and 169 nts downstream.

**(ii)** ***B. cereus***: *B. cereus*, another member of the *B. cereus* group was represented by 211 different strains. A phylogenetic tree based on the 16S rDNA sequences showed 7 clusters – BCI to BCVII (Figure S2). These 7 clusters consisted of 12 to 48 strains. Three strains could not be segregated clearly into any of these clusters. Multiple alignments of all the strains revealed the conserved region in each of the 7 clusters. A visual scan of the profiles in each of the clusters BCI to BCVII, reflects that there is around 83.8% (1276/1522 nts) similarity among them. This 1276 nts long sequence may represent the core region of this *Bacillus* species. The 16.22% variability present in the region flanking the core sequence stretch was found to extend up to 86 nts upstream and 142 nts downstream. However, some further similarity was recorded in two sets of clusters i) BCI, BCIV and BCVI, ii) BCV and BCVII, which were thus considered as identical. These two major groups were comprised of 77 and 87 strains, respectively. The final number of *B. cereus* clusters could thus be reduced to 4 from the 7 clusters observed in phylogenetic tree.

**(iii)** ***B. thuringiensis***: The third member of the *B. cereus* group is represented by 108 strains of *B. thuringiensis*. The phylogenetic tree based on the nucleotide sequences of the 16S rDNA gene of these 108 strains were primarily represented by 12 different clusters. The number of strains in each of these 12 clusters, BTI to BTXII, varied from 3 to 17 (Figure S3). Twenty six strains could not be segregated clearly into any of these clusters. Multiple alignments of all members within each group revealed their respective conserved regions. Further alignment of the representative conserved regions from all the 12 clusters BTI to BTXII revealed that a completely conserved stretch of 1322/1516 nts equal to 81.2% was visible. This seems to represent the core region of the *B. thuringiensis*. A visual scan of the profiles of the sequences flanking the core region in each of the BTI to BXII cluster shows that there is 100% similarity between clusters BTIII-BTX and BTV-BTXII, which thus reduced the final tally of *B. thuringiensis* phylogenetic clusters from 12 to 10. The overall genetic variability around the core region extended up to 42 nts upstream and 142 nts downstream.

The 472 strains of the *B. cereus* group could thus be segregated in to 18 clusters, where the core region varied from 1276 to 1322, covering 81.2% to 84.2% of the total 16S rDNA gene length.

#### *B. subtilis*

Apart from the *B. cereus* group of 472 strains, *B. subtilis* is represented by 271 different strains. All the 16S rDNA sequences of *B. subtilis* were segregated in to 30 clusters (BSI to BSXXX) on the phylogenetic tree (Figure S4). Each cluster had 4 to 18 organisms. Thirty nine strains could not be segregated clearly into any of these clusters. Multiple alignments within each of the group revealed the regions which were shared by all the strains. The length of the conserved region within clusters BSI to BSXXX varied from 1190 to 1554 nts and exceptionally was 900 nts long. A further realignment of the representative conserved sequences of each of the cluster revealed a DNA stretch of 1278 nts to be common to all. It represented 82.1% of the total length of the 16S rDNA gene of *B. subtilis*. The 17.9% variability in the flanking regions was found to extend on either side of the core region, up to 180 nts upstream and 174 nts downstream. It also reflects that there is a large genetic diversity in *B. subtilis*. On the basis of a visual scan of the regions flanking the conserved sequences, the 30 BS clusters could be reduced to 26. The four clusters quite similar to each other were BSI-BSII, BSVIII-BSXXI, BSXV-BSXXV and BSXXII-BSXXIV. In addition, to the long and variable regions, there were two unique groups, BSXVII and BSXXIII, where the highly conserved region was intercalated by a highly variable region of 105 nts and 24 nts length, respectively. These were the only two such cases comprised of 14 sequences of *B. subtilis* strains, with this unique characteristic among all the 2146 sequences studied here.

#### *B. licheniformis*

In the RDP/NCBI database, *B. licheniformis* was represented by 131 different strains. These strains were distributed as 13 clusters, BLI to BLXIII on the 16S rDNA phylogenetic tree. Three to 15 organisms were present in each of the clusters (Figure S5). Multiple alignment of these 16S rDNA sequence of all the members in each group revealed the regions which were shared among them. The length of the conserved region in clusters BLI to BLXIII, varied from 1219 to 1497 nts. Further alignment of the representative conserved sequences from each of the clusters revealed a completely conserved core DNA stretch of 1139 nts i.e. at position 210 to 1348. On the basis of this high similarity between the representative core regions equivalent to 74.59% of the total length, the 13 clusters could be reduced to 10. BLI, BLIII, BLVII and BLX, BLXI were of almost indistinguishable among themselves. The long and variable regions flanking the core sequence, indicates the high genetic variability within the *B. licheniformis*. The 25.41% variability in the flanking regions was found to extend on either side of the core region, up to 183 nts upstream and 180 nts downstream.

#### *B. pumilus*

A group of 83 strains represents the *B. pumilus* species in the 16S rDNA RDP database. The phylogenetic tree of 16S rDNA from *B. pumilus* strains showed 12 clusters (Figure S6). Nine strains could not be segregated clearly into any of these clusters. Each of the clusters, BPI to BPXII was represented by 4 to 13 strains. Multiple alignment of gene sequences of members of each of the cluster showed that the conserved regions vary in length from 1215 nts to 1503 nts. Further alignment of the representative 16S rDNA sequences from each of the 12 clusters showed that there is a conserved region of 1215 nts. The core region represented 79.61% of the total length of the *B. pumilus* 16S rDNA sequence. It indicates the extent of similarity within this *Bacillus* species. On the other hand, the genetic variability extends on either side of the core region, up to 103 nts upstream and 205 nts downstream. Taking into account the core region and the flanking regions, the 12 clusters BPI to BPXII did not show any redundancy and could be easily distinguished from each other.

#### *B. sphaericus*

The phylogenetic tree based on the 16S rDNA gene from a small group of 42 strains of *B. sphaericus*, showed 7 clusters (Figure S7). Each of the clusters BSPI to BSPVII was represented by 2 to 12 different strains. Five strains could not be segregated clearly into any of these clusters. Multiple alignment of gene sequences of members of each of the cluster revealed that the conserved regions varied in length from 1179 nts to 1501 nts. Subsequent alignment of the representative 16S rDNA sequences from each of the 7 clusters showed that there is a conserved region of 1081 nts. It represented 72.06% of the total length of the *B. sphaericus* 16S rDNA gene. There is thus a great genetic variability among the different clusters. The variable region extended up to 248 nts upstream of the 16S rDNA core region and 174 nts downstream. When all the clusters and their core regions are taken into account, two clusters BSPI and BSPVI showed high similarity and could be considered as one. The final tally of clusters of *B. sphaericus* could thus be reduced to six.

#### *B. halodurans*

*B. halodurans* group was constituted by 36 strains. Phylogenetic tree of the 16S rDNA sequences could segregate these 36 strains into 4 clusters (Figure S8). These 4 clusters, BHI to BHIV were represented by 3 to 13 strains. Three strains could not be segregated clearly into any of these clusters. Multiple alignment of 16S gene sequences within each group showed that the conserved region varies from 1443 to 1548 nts in length. Further alignment of the representative sequences of each of the group revealed that the core conserved region is 1416 nts long. It represents 91% of the total length. The low variability in the 16S rDNA gene sequence is evident by the length of the flanking regions which varies from 65 to 70 nts. However, the 4 clusters were still maintained in spite of low genetic variability.

#### *Bacillus clausii*

*Bacillus clausii* group is constituted of 39 strains. On the basis of the 16S rDNA gene phylogenetic tree, 6 clusters were observed for 34 of the strains (Figure S9). Five strains did not fall clearly into clusters. The 6 clusters BCI to BCVI were represented by 2 to 10 different strains. Multiple alignments of gene sequences of members of each of the cluster revealed the size of the conserved region, which varied in length from 1343 nts to 1551 nts. However, subsequently when the representative 16S rDNA sequences from each of the 6 clusters were considered, the conserved region was found to be 1337 nts long. It represented 86.2% of the total length of which 16S rDNA gene of *B. clausii* may extend. It reflects that there is quite a large genetic variability among the different clusters. The variable region extends only up to 164 nts upstream of the 16S rDNA core region and 52 nts downstream. In spite of a large homologous region among all the clusters of *B. clausii*, they were still distinguishable from each other particularly the clusters BCII, BCIII and BCVI.

#### *B. megaterium*

The *B. megaterium* group was represented by 47 strains. A phylogenetic tree based on the 16S rDNA gene segregated these 47 strains in to 8 clusters: BMI to BMVIII (Figure S10). Each of the clusters was composed of 2 to 12 isolates, 6 strains could not be clustered concretely. Multiple alignments of gene sequences of members of these 8 clusters revealed the diversity of the core region of the 16S rDNA, which 1240 nts long. It represented 81.2% of the total length of the *B. megaterium* 16S rDNA gene. The 18.8% diversity in the region flanking the core region reflects the extent and range of genetic variability among the clusters BMI to BMVIII. Some of the clusters had high bootstrap value of 963 to 1000, while others had moderate bootstrap value of 562 to 661.

### Phylogeny of *Bacillus* Core Groups

**Figure 1:**
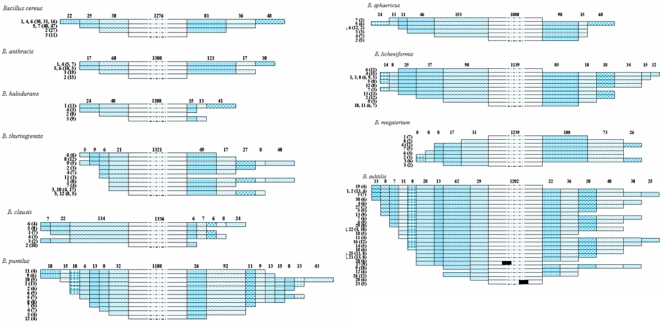
Variability in the terminal regions of the 16S rRNA gene sequences of Bacillus spp.: (a). *B. cereus*; (b). *B. anthracis*; (c). *B. halodurans*; (d). *B. thuringiensis*; (e). *B. clausii*; (f). *B. pumilus*; (g). *B. sphaericus*; (h). *B. licheniformis*; (i). *B. megaterium*; (j). *B. subtilis*. Values represent group number within a species. Values within parentheses represent number of strains within the same group showing ≥95% similarity among them. Values on the top of horizontal bar represent the number of nucleotides showing similarity among groups. Dotted region in the middle is not to scale. Colored pattern corresponds to distinct fragments. Solid black box represents gap with in the sequences in *B. subtilis*.

**Figure 2:**
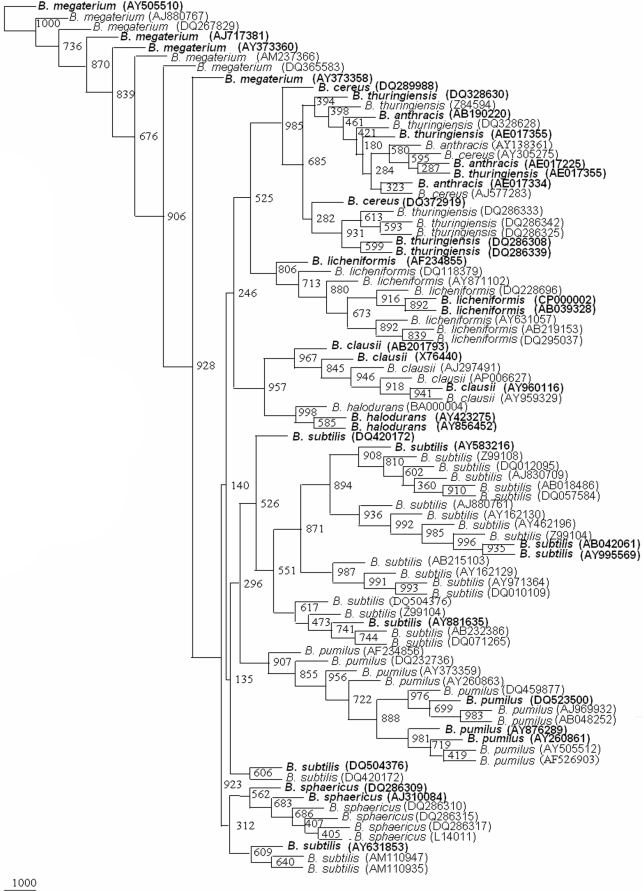
Phylogenetic tree based on 89, 16S rRNA gene sequences from 10 *Bacillus* species. A neighbor-joining analysis with Jukes-Cantor correction and bootstrap support was performed on the gene sequences. Bootstrap values are given at nodes, 1000 bootstrap replicates were run. Bold sequences (34) are the ones considered as framework in the study. Values in parentheses are accession numbers (http://rdp.cme.msu.edu/).

The 1121 strains of *Bacillus cereus* group, *B.subtilis*, *B. licheniformis*, *B. pumilus*, *B. sphearicus B. megaterium*, *B. clausii* and *B. halodurans* strains could be represented by 89 clusters/sequences (Figure 1). A phylogenetic tree drawn on the basis of the representative sequences of the 16S rDNA gene of the 89 clusters (Figure 2) could segregate 10 *Bacillus* species into different clusters (bootstrap value varied from 135 to 716). Members of *B. sphaericus*, *B. halodurans*, *B. pumilus* and *B. licheniformis*, *B. clausii* and *B. megaterium* were very clearly segregated. Since *B. subtilis* clusters were subdivided into two groups. *B. subtilis* Gr I was more similar to *B. sphaericus* (bootstrap value 312) whereas *B. subtilis* Gr II was more close to *B. pumilus* (bootstrap value 296). *B. subtilis* group of 271 strains was in fact observed to form 26 clusters on their 16S rDNA gene phylogenetic tree, which is indicative of greater diversity. *B. cereus* group members, *B. cereus*, *B. thuringiensis* and *B. anthracis* were placed next to each other on the 16S rDNA phylogenetic tree. Except one subclade consisting only of *B. thuringiensis* strains, the other clade was represented by all the three members. Members from each of the clusters were selected to define the range of genetic variability in each of the *Bacillus* species. From each clade, two members were selected for representing *B. sphaericus*, *B. subtilis* Gr I, *B. halodurans*, *B. cereus*. For *B. pumilus*, *B. anthracis*, *B. licheniformis* and *B. clausii* three members were chosen to represent them. Four members of *B. subtilis* Gr II and *B. megaterium* and five members of *B. thuringiensis* were selected to represent these species. Thus 34 sequences were chosen to represent the 10 *Bacillus* species (Figure 2). This reference phylogenetic framework tree has been used to segregate those *Bacillus* isolates which have been presently classified only as *Bacillus* sp.

**Figure 3:**
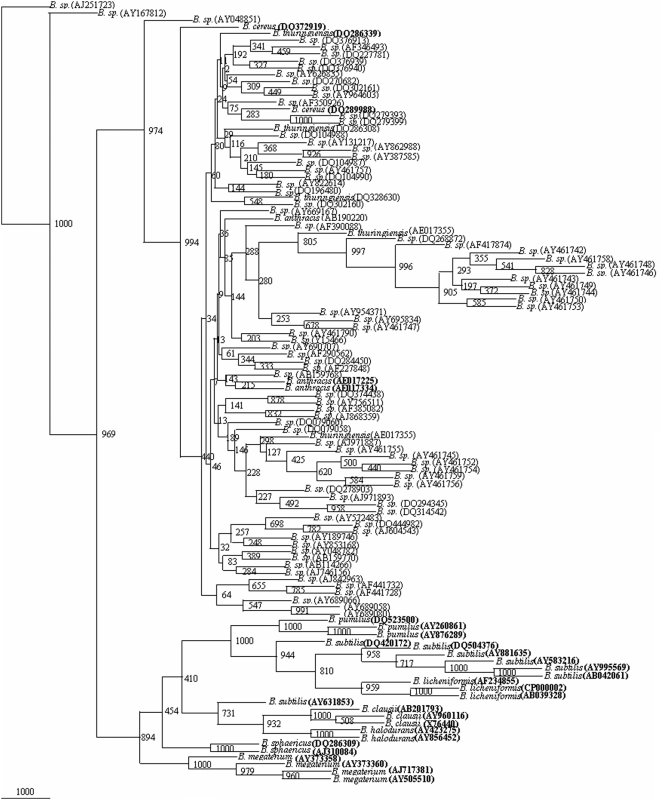
Phylogenetic tree of 34 framework sequences (bold values) and 78 *Bacillus* sp., which could be designated as *B. thuringiensis*, *B. anthracis* and *B. cereus* (Figures S11, S12, S13, S14, S15, S16, S17, S18, S19, S20, S21, S22, S23, S24, S25, S26, S27, S28, S29, S30, S31 to S32 and Table S1). A neighbor-joining analysis with Jukes-Cantor correction and bootstrap support was performed on the gene sequences. Bootstrap values are given at nodes, 1000 bootstrap replicates were run. Values in parentheses are accession numbers (http://rdp.cme.msu.edu/).

A total of 1025 sequences of 16S rDNA gene from *Bacillus* sp. were screened at the rate of 52 entries along with 34 reference phylogenetic framework sequences to generate 22 phylogenetic trees. From all the phylogenetic trees it was possible to classify a total of 305 isolates of the *Bacillus* sp: 75 isolates as *B. cereus*, 2 isolates as *B. thuringiensis*, 44 isolates as *B. subtilis*, 21 isolates as *B. licheniformis*, 32 isolates as *B. pumilus*, 23 isolates as *B. sphaericus*, 7 isolates as *B. halodurans*, 69 isolates *B. megaterium*, 31 isolates as *B. clausii* and 1 as *B. anthracis*. Final phylogenetic trees were drawn on the basis of this preliminary screening showed that 305 out of these 1025 show more similarity having 800 to 1000 bootstrap values with their respective species (Figures 3–7). The accession numbers of these *Bacillus* species are given in Table S1. At this rate about 29.75% of the unclassified *Bacillus* sp. could be identified up to species level. This phylogenetic framework based on 34, 16S rDNA sequences from 10 *Bacillus* species can be used to identify *Bacillus* strains up to species level.

**Figure 4 pone-0004438-g004:**
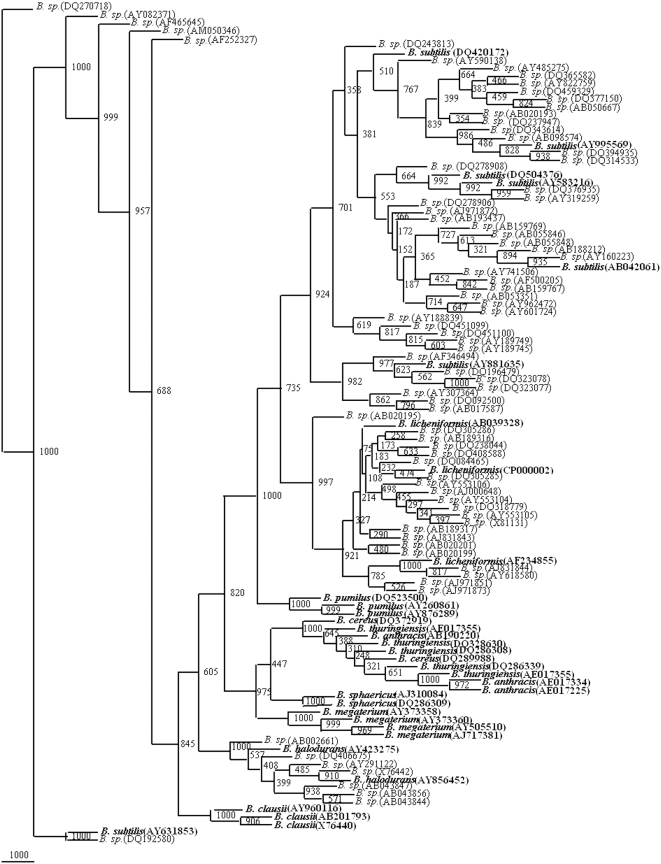
Phylogenetic tree of 34 framework sequences (bold values) and 72 *Bacillus* sp., which could be designated as *B. subtilis*, *B. licheniformis* and *B. halodurans* (Figures S11, S12, S13, S14, S15, S16, S17, S18, S19, S20, S21, S22, S23, S24, S25, S26, S27, S28, S29, S30, S31 to S32 and Table S1). A neighbor-joining analysis with Jukes-Cantor correction and bootstrap support was performed on the gene sequences. Bootstrap values are given at nodes, 1000 bootstrap replicates were run. Values in parentheses are accession numbers (http://rdp.cme.msu.edu/).

**Figure 5 pone-0004438-g005:**
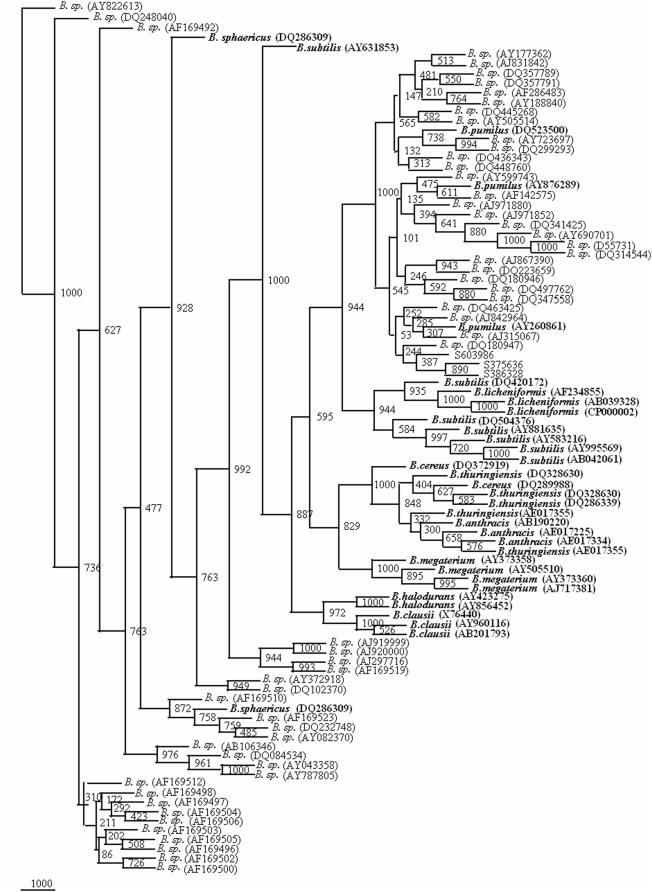
Phylogenetic tree of 34 framework sequences (bold values) and 55 *Bacillus* sp., which could be designated as *B. sphaericus* and *B. pumilus* (Figures S11, S12, S13, S14, S15, S16, S17, S18, S19, S20, S21, S22, S23, S24, S25, S26, S27, S28, S29, S30, S31 to S32 and Table S1). A neighbor-joining analysis with Jukes-Cantor correction and bootstrap support was performed on the gene sequences. Bootstrap values are given at nodes, 1000 bootstrap replicates were run. Values in parentheses are accession numbers (http://rdp.cme.msu.edu/).

**Figure 6 pone-0004438-g006:**
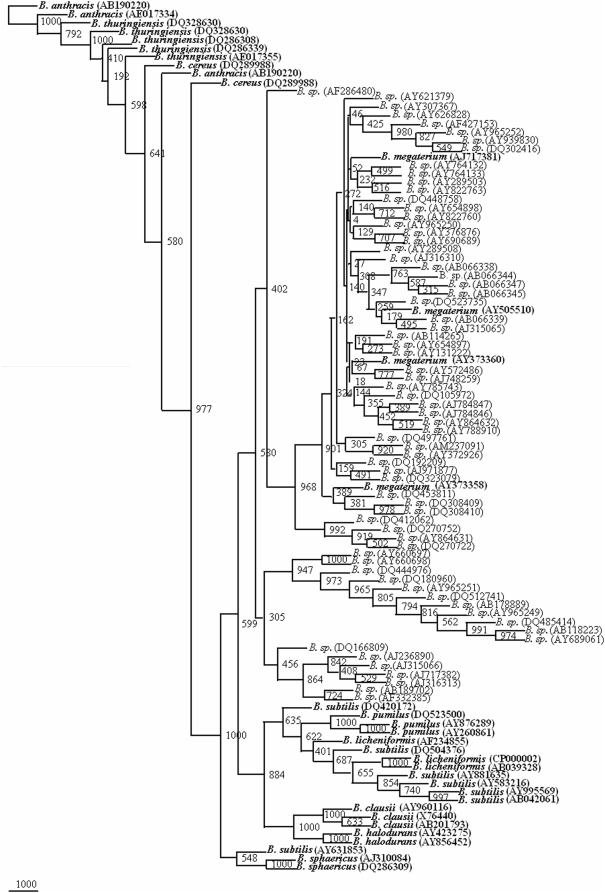
Phylogenetic tree of 34 framework sequences (bold values) and 69 *Bacillus* sp., which could be designated as *B. megaterium* (Figures S11, S12, S13, S14, S15, S16, S17, S18, S19, S20, S21, S22, S23, S24, S25, S26, S27, S28, S29, S30, S31 to S32 and Table S1). A neighbor-joining analysis with Jukes-Cantor correction and bootstrap support was performed on the gene sequences. Bootstrap values are given at nodes, 1000 bootstrap replicates were run. Values in parentheses are accession numbers (http://rdp.cme.msu.edu/).

**Figure 7:**
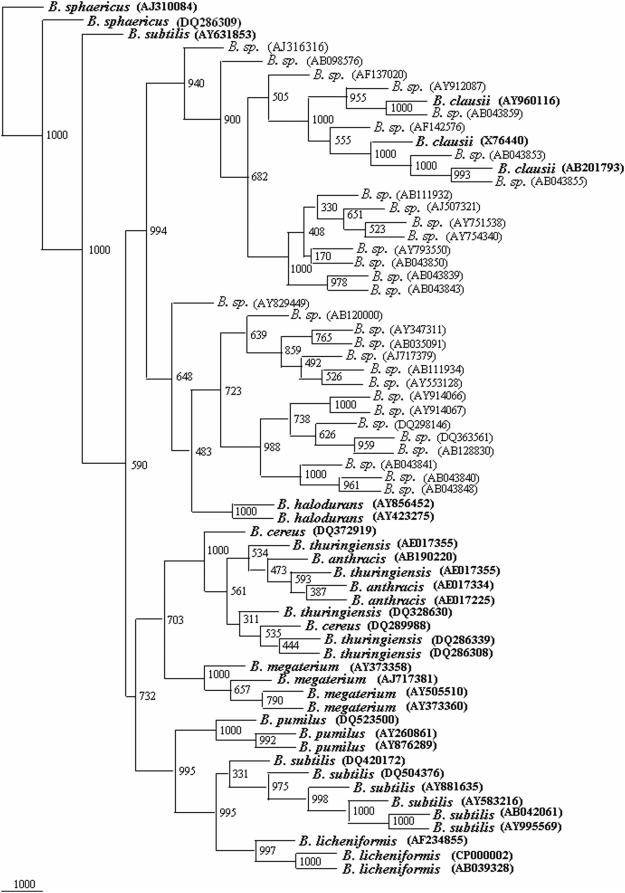
Phylogenetic tree of 34 framework sequences (bold values) and 31 *Bacillus* sp., which could be designated as *B. clausii* (Figures S11, S12, S13, S14, S15, S16, S17, S18, S19, S20, S21, S22, S23, S24, S25, S26, S27, S28, S29, S30, S31 to S32 and Table S1). A neighbor-joining analysis with Jukes-Cantor correction and bootstrap support was performed on the gene sequences. Bootstrap values are given at nodes, 1000 bootstrap replicates were run. Values in parentheses are accession numbers (http://rdp.cme.msu.edu/).

### Signature Analysis

#### *Bacillus* species-specific signature

The sequences of 10 data sets were submitted groupwise to MEME program (http://meme.nbcr.net/meme/meme.html). Ten signatures were identified for each species, which were 25–30 nts long (Table S2). Signature, which was not present in other *Bacillus* spp. and found as distinct was used to blast against the NCBI database. As seen from the Table 2, there were 1–5 regions in each of the *Bacillus* species, which had the potential to be used as signature except in case of *B. anthracis*, *B. licheniformis*, *and B. subtilis*. Signature analyses of 16S rDNA sequences of known *Bacillus* spp. show unique signatures exclusive to them. *B. cereus* and *B. thuringiensis* had unique 16S rDNA signatures of 29 to 30 nts length. These occurred with a frequency of 151/211 and 43 to 63/108 sequences. By this approach, no signatures specific to *B. anthracis* alone could be detected. It may be remarked that among the 3 members of the *B. cereus* group, *B. cereus* shared 8/10 signatures with *B. anthracis* and 5/10 signatures with *B. thuringiensis*. Four out of 10 signatures were shared by all the three members. *B. clausii*, *B. megaterium* and *B. halodurans* can be identified with the aid of 2 to 3 signatures. For *B. licheniformis* and *B. subtilis*, no unique signatures could be identified. Some signatures such as 5′TGTGGTTTAATTCGAAGCAACGCGAAGAA 3′ were shared by all the *Bacillus* spp. except *B. subtilis*, *B. pumilus* and *B. sphaericus*, providing “evidences” of common origins. Similarly, certain signatures 5′TAAAGCTCTGTTGTTAGGGAAGAACAAGT 3′ were shared only between *B. subtilis* and *B. pumilus*. Such similarities in signature sequences were also recorded among *B. licheniformis* and *B. megaterium* on one hand and *B. licheniformis* and *B. pumilus* on the other. For all these signatures, the closest match within the top 50 best hits (BLAST) with their respective species was in the range of 60 to 98%. Exceptionally the best matches were in the lower range of around 24% in the case of *B. cereus*.

**Table 2:** Characteristics of unique nucleotide signatures for 16S rDNA sequences of different *Bacillus* spp. and clusters of *Bacillus* sp.

*Bacillus* spp./Cluster^a^	Signature	Length (nts)^b^	Frequency^c^
***Bacillus cereus***	AAAGTGGAATTCCATGTGTAGCGGTGAAAT	30	151/211
***B. thuringiensis***	ATAACATTTTGAACTGCATGGTTCGAAATT CTTTAGTGCTGAAGTTAACGCATTAAGCA	3029	43/10863/108
***B. anthracis***	No unique signature was detectable		0/153
***B. clausii***	AATCCCATAAAGCCATTCTCAGTTCGGATT AAATGATTGGGGTGAAGTCGTAACAAGGTAAAACCGGAGCTAATACCGGATAATCCCTTT GCATTAGCTAGTTGGTAAGGTAACGGCTTA GTAGTGCCGAAGTTAACACATTAAGCACT	30 30303029	14/39 31/3914/39 15/39 23/39
***B. halodurans***	ATAATAAAAAGAACTGCATGGTTCTTTTTT ACCAAAGGGAGCTTGCTCCTAGAGGTTAGC	3030	21/3601/36
***B. pumilus***	AAGGTTTAGCCAATCCCACAAATCTGTTCT AAGGTTTAGCCAATCCCATAAATCTGTTCT ATAGTTCCTTGAACCGCATGGTTCAAGGAT	303030	14/8336/8342/83
***B. megaterium***	ATGATTGAAAGATGGTTTCGGCTATCACTT AATCCCATAAAACCATTCTCAGTTCGGATT AACTGATTAGAAGCTTGCTTCTATGACGTT TCTTGACATCCTCTGACAACTCTAGAGATA TGGGATAACTTCGGGAAACCGAAGCTAATA	3030303030	36/4719/4728/4733/4734/47
***B. sphaericus***	TAAAACTCTGTTGTAAGGGAAGAACAAGTA ATAGTGGAATTCCAAGTGTAGCGGTGAAAT TAATCCGATAAAGTCGTTCTCAGTTCGGAT AGTAACACGTGGGCAACCTACCTTATAGTT TAACTGGCTGTACCTTGACGGTACCTTATT	3030303030	23/4214/4223/4210/4227/42
***B. subtili*** *s*	No unique signature was detectable		0/211
***B. licheniformis***	No unique signature was detectable		0/131
***Bacillus*** ** sp. Cluster 1**	TAAAACTCTGTTGTAAGGGAAGAACAAGTA AATCCCATAAAACCGTTCCCAGTTCGGAT	3029	19/4605/46
***Bacillus*** ** sp. Cluster 5**	AATCCCATAAATCTATTCTCAGTTCGGATT	30	05/32
***Bacillus*** ** sp. Cluster 6**	AAGCAAATCCCATAAAACCATTCTCAGT TCAAGCAAATCCCATAAAACCATTCTCAGT	2830	09/2307/23
***Bacillus*** ** sp. Cluster 7**	ATAACTCATTTCCTCGCATGAGGAAATGTT	30	10/37
***Bacillus*** ** sp. Cluster 9**	AATCCCATAAAACCACTCTCAGTTCGGATT	30	01/25
***Bacillus*** ** sp. Cluster 10**	AATCCCACAAAACCGTTCCCAGTTCGGATT	30	03/25
***Bacillus*** ** sp. Cluster 11**	TAAACGATGAGTGCTAAGTGTTAGAGGGGT GTCGTAAAGCTCTGTTGTGAGGGACGAAGG	3030	23/5025/50

a Cluster represent isolates defined only up to genus level *Bacillus* sp.

b nucleotides.

c Frequency of occurrence of the signature out of the total sequences screened.

Out of 11 Clusters, no unique signatures were detectable in Clusters 2, 3, 4 and 8.

#### Cluster-specific signature

**Figure 8:**
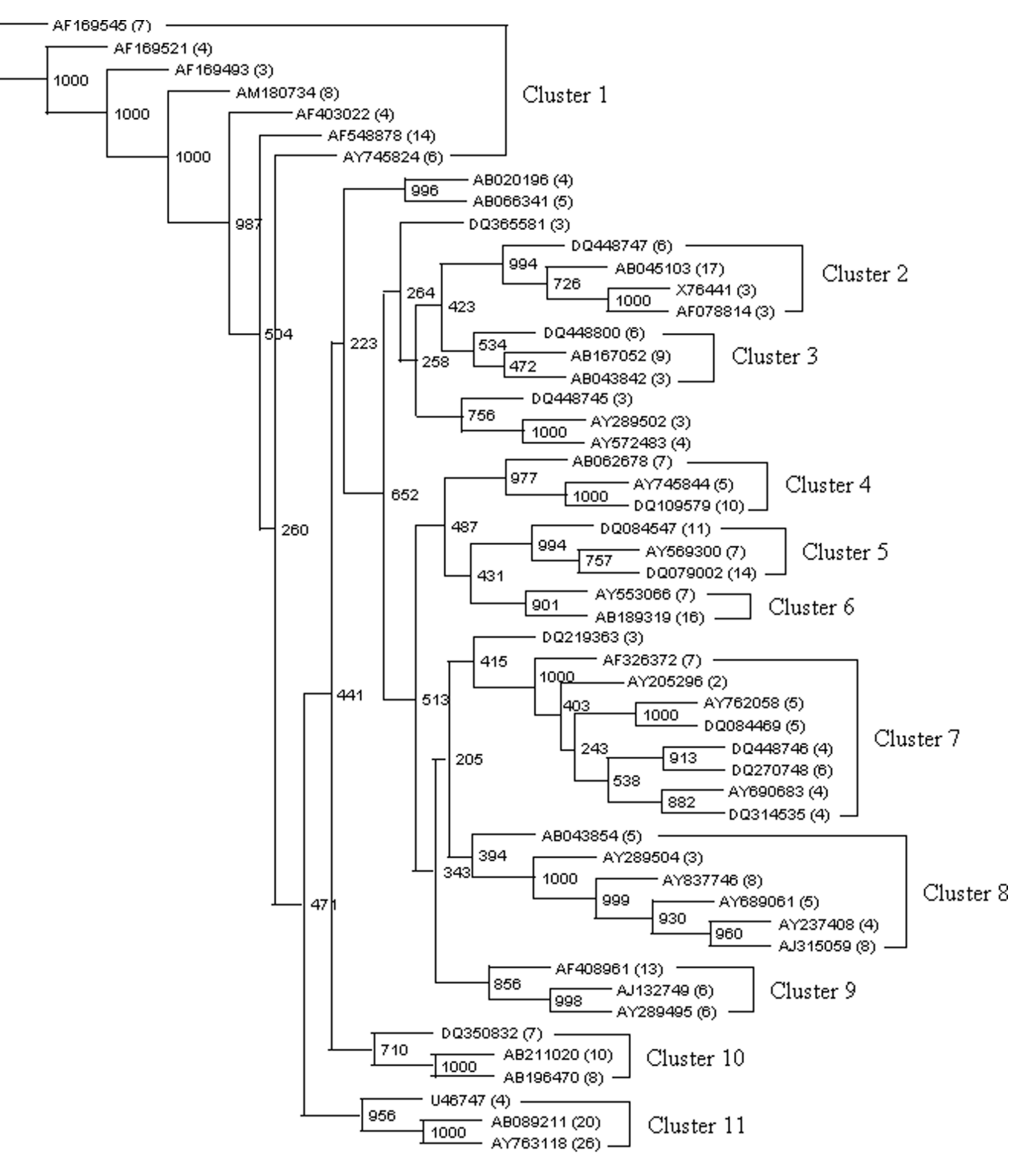
Phylogenetic tree of 52 representative sequences (appeared as 52 clusters {equivalent to 366 sequences} in Figures S11, S12, S13, S14, S15, S16, S17, S18, S19, S20, S21, S22, S23, S24, S25, S26, S27, S28, S29, S30, S31 to S32). These 52 representative sequences were observed to segregate in 11 clusters: cluster 1 to cluster 11. A neighbor-joining analysis with Jukes-Cantor correction and bootstrap support was performed on the gene sequences. Bootstrap values are given at nodes, 1000 bootstrap replicates were run. Accession numbers of the representative sequences are given and values in parentheses against these are equal to the number of sequences in that group within each cluster (http://rdp.cme.msu.edu/).

In case of 11 clusters (Figure 8), on the basis of the ClustalW alignment, the size of the conserved region varied from 1226 to 1547 nts. These 11 clusters consisting of 18 to 50 isolates each did not appear to fall within any of the aligned species and may represent sub-species or novel lineages. The sequences of these 11 clusters were groupwise submitted to MEME program (http://meme.nbcr.net/meme/meme.html). Ten signatures were identified for each cluster which was 25–30 nts long (Table S3). As seen from the Table, there were 1–2 regions in each of the cluster, which had the potential to be used as signature except in case of Cluster 2–4 and Cluster 8 (Table 2). The signatures identified through MEME program revealed that there are certain similarities and even overlapping sequence stretches. The usage of signature and the conserved sequence regions against all other known *Bacillus* spp. indicate that they were not significantly similar and are not homologous. The signature sequence - 5′TTTAATTCGAAGCAACGCGAAGAACCTTA3′ - shared by all the Clusters except 5 and 7 also indicates that these sequences may have a common origin similar to *Lactobacillus*, *Paenibacillus*, *Clostridium*, rumen bacterium, etc. [105]. Each of the clusters has certain unique signatures, which did not match with the 10 *Bacillus* spp. Typically, Clusters 2, 3, 4 and 8 did not show any unique signatures by this approach. However, in Cluster 2 – 5′AAATGATTGGGGTGAAGTCGTAACAAGGTA3′ was the only signature which showed remarkable closeness to *B. clausii* in 31 out of 39 sequences. Similarly, only one out of 10 signatures in Clusters 3 and 4 showed closest matches with *B. licheniformis* and *B. megaterium* at a frequency of 54/131 and 13/47 sequences, respectively. As far as Cluster 8 is concerned, one of the signatures could be traced among 18/37 sequences of Cluster 7 and with negligible frequency in other clusters.

The unique signatures of sequences present in Cluster 1 showed similarity to uncultured *Bacillus* sp. and uncultured *Macrococcus* sp. with a frequency of 28% among the top 50 hits (BLAST). Similarly, the signatures of Clusters 6, 9 and 11 shared similar sequences to *Virgibacillus*, *B. megaterium* and *Geobacillus*, respectively, at a frequency in the range of 16 to 74%. In spite of employing all approaches to assign the signatures and sequences to known organisms, the signatures of Clusters 5, 7 and 10 could not be properly categorized. In brief, all these 11 clusters consisting of 18 to 50 sequences each did not appear to fall within any of the aligned species and may represent sub-species or novel lineages. It is probable that the *Bacillus* community is more diverse than reported so far (http:// rdp.cme.msu.edu). This suggests that bacterial communities in a variety of soils, environmental habitats, etc., may be very similar when assessed by molecular methods for their 16S rDNA than for other metabolic genes.

### Restriction Enzyme Analysis

A total of 14 Type II restriction enzymes, which are independent of methylase and cleave at very specific sites within or close to the recognition sequence (4 to 6 mer) (Table 3), varied in their response to 632, 16S rDNA gene sequences: 344 belonging to 10 known species of *Bacillus* and 288 belonging to strains classified as *Bacillus* sp. Restriction enzymes (REs) sites for *Sma*I, *Eco*RI, *Dpn*II, *Rsa*I, *Bfa*I, *Hae*III, *Tru*9I and *Alu*I in 16S rDNA gene sequences occurred with a frequency of 1 to 7 resulting in 2 to 9 fragments of varied nucleotide lengths. On the other hand, enzymes *Bam*HI, *Not*I, *Sac*I, *Nru*I, *Hin*dIII and *Pst*I proved to be “non”-cutters in most of the cases. Of these later 6 REs, *Bam*HI, *Not*I, and *Sac*I could cut only 0.3 to 2.3% of the 344 sequences belonging to 10 known species of *Bacillus*: *B. cereus*, *B. anthracis*, *B. thuringiensis*, *B. subtilis*, *B. licheniformis*, *B. pumilus*, *B. sphaericus*, *B. halodurans*, *B. megaterium*, *B. clausii*. In spite of low frequency of RE sites for *Nru*I, *Hin*dIII and *Pst*I in 16S rDNA genes sequences, these can be still exploited for distinguishing different *Bacillus* spp. such that i) *Nru*I could cleave only *B. subtilis* and *B. sphaericus* at the rate of 12/30 and 41/42 sequences, respectively; ii) *Hin*dIII sites appeared in 43/47 sequences of *B. megaterium*; iii) *Pst*I sites occurred at high frequency of 35/42 in *B. sphaericus*; 28/30 in *B. pumilus*; 44/47 in *B. megaterium*; 29/30 in *B. licheniformis* and 5/30 in *B. subtilis* (Table S4). The sites for the two enzymes *Eco*RI and *Sma*I appeared in 96 to 97% of the sequences but due to the presence of only one site per sequence, these two enzymes could not serve any significant purpose at this stage.

**Table 3:** Restriction enzyme (Type II) used in the study with their cut sites (rebase.neb.com/rebase/rebase.html).

S. No.	Restriction Enzyme	Cut site
1.	*Alu*I	AG↓CT
2.	*Bam*HI	G↓GATCC
3.	*Bfa*I	C↓TAG
4.	*Dpn*II	↓GATC
5.	*Eco*RI	G↓AATTC
6.	*Hae*III	GG↓CC
7.	*Hin*dIII	A↓AGCTT
8.	*Not*I	GC↓GGCCGC
9.	*Nru*I	TCG↓CGA
10.	*Rsa*I	GT↓AC
11.	*Sac*I	GAGCT↓C
12.	*Sma*I	CCC↓GGG
13.	*Tru*9I	T↓TAA
14.	*Pst*I	CTGCA↓G

**Figure 9:**
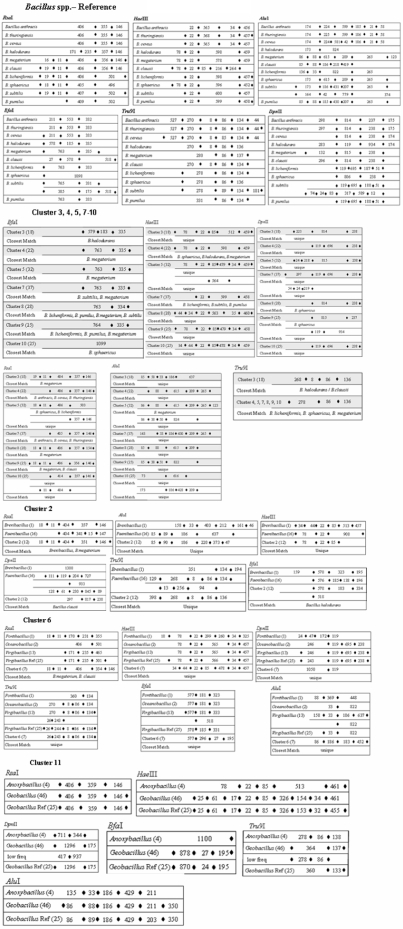
Nucleotide fragments generated as a result of *in silico* restriction enzyme (a). *Rsa*I (b). *Hae*III (c). *Alu*I (d). *Bfa*I (e). *Tru*9I (f). *Dpn*II, action on 16S rDNA gene sequences in 10 *Bacillus* spp. and 11 clusters (Figure 8). A solid black box represents the restriction enzyme cut site. At the terminal points, solid black box are shown only if highly variable fragments are observed beyond this cut site.

Six REs with more than 1 site for their action were compared for all the 344 sequences of 10 *Bacillus* species (Figure 9). The pattern of the number and lengths of the fragments served as reference standards for those strains which have been designated so far as *Bacillus* sp.

A comparative analysis of *in silico* cleavage of 344 sequences of 10 *Bacillus* spp. revealed very unique patterns. With *Rsa*I, 3 fragments of 406, 355 and 146 nts (in this order from 5′ end) were obtained for *B. cereus*, *B. anthracis*, *B. thuringiensis*. *B. megaterium* and *B. clausii* had two fragments of 16/19 and 11 nts in addition to the 3 recorded with the 3 members of *B. cereus* group. *B. subtilis*, *B. licheniformis* and *B. sphaericus* showed similar patterns of fragment size and order: 18/19 – 11 – 405/406/407 – 496/501/502. *B. pumilus* appeared to have lost two 5′ RE sites for *Rsa*I such that only two clear cut fragments could be recorded with high frequency. *B. halodurans* seems to have acquired an additional RE site and assumed a status intermediate to *B. cereus* group on one hand and the rest of the *Bacillus* spp. on the other.

A very distinct order and length of the fragments could be seen with the other 5 REs - *Dpn*II, *Bfa*I, *Hae*III, *Tru*9I and *Alu*I as well (Figure 9). A comparison of *in silico* digestions of the 16S rDNA gene sequences with all the 6 REs revealed two features. *B. subtilis* showed two distinct subgroups with *Dpn*II, *Bfa*I and *Alu*I. Here, no similarities in size of the fragments and the positions of the RE sites were evident within each of the three sets of subgroups. With *Dpn*II, *B. subtilis* revealed 4 fragments in one group: 119-196-87-51 (nts) and 6 fragments of 77-24-83-317-589-12 (nts) in the other. The three additional RE sites appeared on the 5′ end of the 16S rDNA genes. Similarly with *Alu*I, *B. subtilis* groups I and II differed not only in the number of RE sites but also in their positions. It may not be too premature to conclude that *B. subtilis* need to be subdivided in to at least two groups, since a similar situation was observed with respect to the signature analyses presented in the previous sections. Nakamura et al. [77] and Chun and Bae [10] divided *B. subtilis* in to two subspecies, namely *B. subtilis* subsp. *subtilis* and *B. subtilis* subsp. *spizizenii* on the basis of cell wall chemistry and DNA-DNA relatedness data.

**Figure 10:**
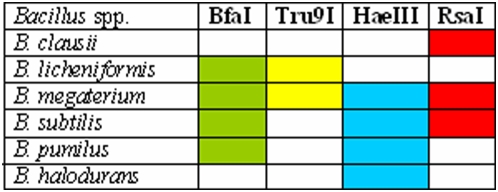
*Bacillus* species showing distinct patterns with different restriction enzymes.

The second distinct feature which emerged is with regards to the classification of these 10 *Bacillus* spp. on the basis of the 6 REs is with respect to members of the *B. cereus* group - *B. cereus*, *B. anthracis*, *B. thuringiensis*, which were indistinguishable within the group. However, they were very clearly distinguishable from all other *Bacillus* spp. with respect to the action of *Dpn*II and *Alu*I. *B. halodurans* could be distinguished from all other species on the basis of a combination of actions of *Tru*I and *Hae*III. *B. clausii* could be segregated from the others by using *Tru*9I and *Rsa*I. *B. megaterium* though quite close to *B. licheniformis* for *Bfa*I and *Tru*9I and *B. clausii* for *Rsa*I; was distinguishable by cutting its 16S rDNA gene sequences with a set of 4 REs: *Bfa*I, *Tru*9I, *Hae*IIII and *Rsa*I. *B. megaterium* appears to be the originator of *B. halodurans*, *B. clausii*, *B. licheniformis*, *B. subtilis* and *B. pumilus*, with whom it shares different RE sites and fragments lengths (Figure 10).

*B. sphaericus* shows similarity to *B. clausii* and *B. halodurans* with respect to *Tru*9I; to *B. licheniformis* and *B. subtilis* for *Hae*III and *Rsa*I and to *B. pumilus* for *Hae*III. Hence a combination of 3 enzymes *Tru*9I, *Hae*III and *Rsa*I will be necessary to distinguish it from its closely resembling species. *B. subtilis* is quite similar to *B. licheniformis* but can be distinguished on the basis of *Tru*9I. On the other hand *B. subtilis* and *B. pumilus*, which show similar RE patterns for *Bfa*I and *Hae*III, can be separated on the basis of *Rsa*I (Figure 10).

### Pattern of restriction enzyme sites in *Bacillus* spp

On the basis of sequence similarity (phylogenetic closeness), and signature patterns (previous sections), 288 *Bacillus* sp. and 47 sequences of *Jeotgalibacillus*, *Brevibacillus*, *Geobacillus*, *Marinibacillus*, *Paenibacillus*, *Pontibacillus* and *Virgibacillus*, etc. could be segregated in to 11 clusters. RE patterns of the 344 16S rDNA sequences of 10 *Bacillus* spp. served as references for the segregation of strains designated as *Bacillus* sp. (Figure 9).

Out of the 14 TypeII REs employed for *in silico* digestions, *Sma*I, *Eco*RI, *Dpn*II, *Rsa*I, *Bfa*I, *Hae*III, *Tru*9I and *Alu*I could cleave 16S rDNA gene sequences with a frequency of 1 to 7 as was observed with known *Bacillus* spp. RE sites for *Bam*HI, *Not*I, *Sac*I occurred with a very low frequency of 0.4 to 3.0% and thus proved to be “non”-cutters. On the other hand, the sites for enzymes *Nru*I, *Hin*dIII and *Pst*I were observed to occur with moderate frequency in some of the clusters and could not be detected in some others. *Sma*I and *Eco*RI sites were observed with high frequency but with only one site per sequence, strong conclusions were difficult to draw. Hence, once again, information based on 6 REs - *Dpn*II, *Rsa*I, *Bfa*I, *Hae*III, *Tru*9I and *Alu*I - generating more than 2 fragments proved useful for reaching meaningful conclusions.

On the basis of RE patterns (Figure 9), Cluster 1 comprising of 46 sequences (22 *Bacillus* sp. and 24 sequences of *Jeotgalibacillus*, *Marinibacillus*, *Ureibacillus* and *Sporosarcina*) showed similarity to 4 different *Bacillus* spp.: *B. subtilis*, *B. licheniformis*, *B. sphaericus* and *B. halodurans* (Table 4). Clusters numbered 4 and 7, showed similarity to 7 and 8 *Bacillus* spp., respectively. Incidentally, except for *Tru*9I, pattern of fragment length (nts) and order showed that Clusters 1, 4 and 8 resemble *B. licheniformis*, *B. sphaericus*, *B. megaterium*. However, there was quite a bit of variation among these clusters for the other 5 REs, which implies that the organisms in these clusters might have a common origin but are presently well distinguishable from each other. Clusters numbered 2, 3, 5, 6, 8, 9, and 10 showed RE fragment length and order pattern to be quite similar to 1 to 5 of the known *Bacillus* spp. but for certain REs there was no resemblance to known species. Clusters numbered 3, 6, 9 and 10 were quite distinct and hence categorized as “unique” on the basis of their response to *Alu*I, *Dpn*II and *Hae*III, The fragment lengths and order were unique to each of them. A comparison of the different Clusters numbered 3, 6, 9, 10 with respect to their similarity to known species revealed that no two clusters showed exact match. The only exception to this observation, were the clusters 6 and 7 with respect to the action of HaeIII, where the order and size of the fragments were quite similar. For *Bfa*I, *Rsa*I and *Tru*9I, the similarity to known *Bacillus* spp. varied from cluster to cluster in two respects: firstly, the species identification varied from RE to RE, and secondly, no two clusters showed similar results with any two REs (Table 4).

**Table 4:** Closest matches of Clusters of *Bacillus* sp. with known *Bacillus* sp. on the basis of restriction enzyme pattern analysis.

*Bacillus* sp.	Restriction enzyme						No. of Best matches	
	*Bfa*I	*Dpn*II	*Rsa*I	*Tru*9I	*Alu*I	*Hae*III	*Bacillus* spp.	Unique^a^
**Cluster 1 (22)** b	*B. sphaericus*	unique	*B. licheniformisB. subtilis*	*B. licheniformis*	unique	*B. sphaericusB. halodurans*	4	2
**Cluster 2 (12)**	*B. halodurans*	*B. clausii*	*B. megaterium*	unique	unique	Unique	3	3
**Cluster 3 (18)**	*B. halodurans*	unique	*B. megaterium*	*B. haloduransB. clausii*	unique	Unique	3	3
**Cluster 4 (22)**	*B. megaterium*	unique	*B. anthracisB. cereusB. thuringiensis*	*B. licheniformisB. megateriumB. licheniformis*	unique	*B. sphaericusB. haloduransB. megaterium*	7	2
**Cluster 5 (32)**	*B. megaterium*	unique	*B. sphaericusB. licheniformis*	*B. licheniformisB. megateriumB. sphaericus*	*B. megateriumunique*	Unique	3	3
**Cluster 6 (7)**	Unique	unique	*B. megateriumB. clausii*	unique	unique	Unique	2	5
**Cluster 7 (37)**	*B. subtilisB. megaterium*	unique	*B. anthracisB. cereusB. thuringiensis*	*B. licheniformisB. megateriumB. sphaericus*	unique	*B. licheniformisB. subtilisB. pumilus*	8	2
**Cluster 8 (28)**	*B. pumilusB. megateriumB. licheniformisB. subtilis*	*B. sphaericus*	*B. megaterium*	*B. licheniformis B. megateriumB. sphaericus*	unique	Unique	5	2
**Cluster 9 (25)**	*B. licheniformisB. pumilusB. megaterium*	*B. sphaericus*	*B. megateriumB. clausii*	*B. licheniformis B. megateriumB. sphaericus*	unique	unique	5	2
**Cluster 10 (25)**	*B. sphaericus*	unique	unique	*B. licheniformisB. sphaericusB. megaterium*	unique	unique	3	4
**Cluster 11**	All were designated^c^							

a Did not match with any of the known RE patterns of the *Bacillus* sp. used in this study.

b Number of 16S rDNA sequences of *Bacillus* sp.

c Cluster 11 constituted of members of known species such as: *Anoxybacillus* and *Geobacillus.*

Clusters 1, 2, 6 and 11 were composed of *Bacillus* sp. which could be categorized in to two groups: Group 1 consisted of *Bacillus* sp., which has now been reclassified as *Jeotgalibacillus*, *Marinibacillus*, *Ureibacillus*, *Sporosarcina* and Group 2 consisted of those which have been defined only up to genus level (*Bacillus* sp.). These served as controls for the observations made for *Bacillus* spp. with different REs. Cluster 1 showed closest matches with *Marinibacillus*, *B. subtilis* and *B. licheniformis* for *Rsa*I (Figure 9). Such a dual relationship was also recorded for Cluster 1 with *Bfa*I, *Dpn*II and *Tru*9I. In these cases, the closest matches were *Sporosarcina*, *Jeotgalibacillus*, *Marinibacillus*, *B. licheniformis* and *B. sphaericus*. The variability in the closest matches among different REs reflects that Cluster 1 is a “unique “ group intermediate to *Bacillus* and other closely related species such as *Jeotgalibacillus*, *Marinibacillus*, *Sporosarcina*, etc. Similarly, Clusters 2 and 6 showed uniqueness in their fragment order and length with 3 and 5 different REs, respectively. It implies that these groups of organisms need attention from taxonomists and may be classified as new organisms. Cluster 11 turned out to be primarily a set of 46 sequences of *Geobacillus* and 4 sequences of *Anoxybacillus*. These sequences largely resembled known *Geobacillus* sp. with respect to their RE activities. It thus served primarily as validation of the results recorded with *Bacillus* sp (Table 4).

## Discussion

Among the group of aerobic endospore-forming bacteria of 25 genera and over 200 species, *Bacillus* is the largest and most prominent. *Bacillus* is comprised by heterogeneous assembly of gram-positive, rod shaped, spore forming bacteria, which may grow aerobically or as facultative anaerobes. Further characterization and identification has been traditionally based on biochemical tests and fatty acid methylester (FAME) analysis [106], [107]. With further developments, API (Analytab Products, Inc) system of identification is quite reproducible and reliable [108]. Microbial genotype or DNA sequence based analytical approaches are highly reproducible and enable large number of samplings at a time [100]. 16S rDNA sequencing has proved to be one of the most powerful tools for the classification of microorganisms [109]–[111]. 16S rDNA sequencing technique has been used for the identification of *Bacillus* spp. such as *B. subtilis* [101]; *B. cereus* and *B. thuringiensis* [102]; *P. alvei* (formerly *B. alvei*) [103].

The genus *Bacillus* has under gone considerable taxonomic changes. The number of spp. within this genus was reduced from 146 in the 5^th^ Edition of Bergey's Manual of Determinative Bacteriology [112] to 22 [113]. In the Approved List of Bacterial Names [114], 31 of the 38 aerobic endospore formers were *Bacillus*. However, at present there are 175 *Bacillus* species (http://rdp.cme.msu.edu/). The two factors for the rapid increase are the application of more diverse and intelligent methods for enrichment and isolation and the development of new and ever more sophisticated methods of amplification and sequencing of genes [115].

The identification of *Bacillus* species on the basis of 16S rDNA gene sequence is done by blasting it against the available databases. The need for developing a tool for identifying *Bacillus* species arose due to two reasons: (i) more than 50% of the 16S rDNA sequences deposited in the databases, have been annotated/identified only as *Bacillus* sp., ii) *B. subtilis* strains were seen to cluster in different clades and widely placed clusters on the phylogenetic tree (Data not shown here) raising doubts about the sequencing quality. It gives an impression that *B. subtilis* perhaps defies the phylogenetic pattern. It also suggests that the genera *Bacillus* may be further divided into sub-species, particularly as far as *B. subtilis* is concerned. Our data imply that the 16S rDNA molecule of the *B. subtilis* may exist in two different states much similar to *B. cereus*. This may lead to a different structure of the 30S subunit since binding of primary binding proteins affects binding of the secondary and tertiary binding proteins [116]. In fact, Nakamura et al. [77] divided *B. subtilis* in to two subspecies, namely *B. subtilis* subsp. *subtilis* and *B. subtilis* subsp. *spizizenii* on the basis of cell wall chemistry and DNA-DNA relatedness data. These were therefore regarded as genomovars. In view of this scenario, we thought of developing a tool which will enable identification of new *Bacillus* isolates at species level. In fact, a few *Bacillus* species, such as *B. kaustophilus*, *B. sterothermophilus*, *B. thermoglucosidasius* and *B. thermoleovorans* have been transferred to the newly created genus *Geobacillus* [65]. Certain others such as *Bacillus globisporus*, *B. pasteurii* and *B. psychrophilus* have been reclassified to the genus *Sporosarcina* [66]. In the same manner, *B. marinus* has been reclassified as *M. marinus* [67].

### Problems with *Bacillus* Species

The members of *B. cereus* group show 99.5 to 100% similarity for their 16S and 23S rDNA sequences [117], [118]. The genetic diversity of *B. cereus* isolates is enhanced by extra chromosomal elements [92], [119]–[122]. The need to develop approaches that rapidly identify the “near neighbours” of *B. cereus* group are of great interest for the study of *B. anthracis* virulence mechanisms as well to prevent the use of such strains for *B. anthracis* based bioweapon development [123]. In this study all the three members of the *B. cereus* group have very large conserved regions and appear indistinguishable on the basis of 16S rDNA gene sequence. *B. thuringiensis* and *B. mycoide*s differ from *B. anthracis* and *B. cereus* by 0 to 9 nucleotides [47]. Even single strand conformation of polymorphism (SSCP) did not allow species discrimination within *B. cereus* group [124]. Variable region VI of 16S rDNAs of *B. cereus* and *B. thuringiensis* are useful for differentiation between these species [125]. However, our approaches have been able to distinguish *B. cereus* and *B. thuringiensis* from *B. anthracis*. With the first approach of using the representative sequences for each species, these three though distinguishable were always placed next to each other. On the other hand, with specific signature sequence, it was possible to distinguish *B. cereus* from other two. It was difficult to identify species specific signatures for *B. anthracis* and *B. thuringiensis*. A strategy has been proposed by Daffonchio et al. [123] for the identification of near neighbours of *B. anthracis* based on single nucleotide polymorphism (SNP) in the 16S–23S rDNA ITS containing tRNA genes, characteristic of *B. anthracis*. Two *B. cereus* strains and one *B. thuringiensis* strains showed RSI-PCR profiles identical to that of *B. anthracis*. The strict relationship with *B. anthracis* was confirmed by MLST of four independent loci: the 16S–23S rDNA long ITS, the SG-749 fragment that included a region homologous to *B. subtilis ypnA* gene; the AC-390 fragment that is homologous to the *B. subtilis ywfK* gene, encoding a hypothetical transcriptional regulator belonging to the LysR family; the *pleR* gene encoding for a pleiotropic regulator previously identified as one of the principal regulators of *B. cereus* virulence gene and the *cerA* gene that encodes the cereolysin A phospholipase. In yet another population genetic study among a strain collection of *B. cereus* group species, it was found by MLEE and MLST that the strains could be divided into two main groups [123]. The difficulty in distinguishing *B. cereus* group members on 16S rDNA based diagnosis however, correlated well with gyrase B (*gyr*B) as a molecular diagnostic marker [126].

Rep-PCR has been shown to be a useful technique in the subtyping of *Bacillus* species [37], [38]. However, protein coding genes such as *gyrA* and *rpoB* exhibit much higher genetic variation. These genes have been thus used for the classification of closely related taxa within the *B. subtilis* group [10], [39]. In spite of such difficulties our frame work is proving an efficient tool to handle such problems. With our approach two *B. subtilis* groups could be easily segregated on the basis of 16S rDNA gene sequence itself. These reference sequences in fact could segregate the two subspecies proposed by Nakamura et al. [77] namely *B. subtilis* subsp. *subtilis* and *B. subtilis* subsp. *spizizenii* on the basis of cell wall chemistry and DNA-DNA relatedness data. Of the two strains chosen within the *B. subtilis* Gr1, one of them was highly similar to *B. licheniformis* (Accession No. DQ504376), whereas the other strain was quite distinct (*B. subtilis* Accession No. AY631853).

The large degree of variation in the individual group fingerprints suggests that a substantial intra-species genetic diversity may exist and highlights the very high resolution of 16S rDNA gene sequencing. The data presented here is to our knowledge the first time, where molecular technique has been exploited and applied in this manner for assigning *Bacillus* isolates to different species. It would be difficult to extrapolate to define the potential limits of genetic variability within the *Bacillus*. However, such a strategy can be extended to other genera as well. It could form the basis for developing reference phylogenetic tree for various genera for which large number of 16S rDNA sequencing data is available.

The 16S rDNA sequences therefore give consistent separation of the strains into 9 major, non-overlapping clusters. The significance of this formal cluster assignment is made clear by the inclusion in our data set of sequences from independently reported strains. The signature nucleotide offers the opportunity for designing species-specific probes as primers for a rapid identification/segregation of new isolates.

Since pathogenic capacities of *Bacillus* species are often plasmid linked i.e. the pXO2 encoded capsule gene cluster, it may not necessarily be linked to internal genotypic grouping of taxa. The fact that plasmids can easily be transferred or lost makes these criteria unacceptable for typing purposes. Our reference phylogenetic tree may partially replace the use of schemes such as MLST.

### Signatures

16S rDNA sequences vary due to substitutions and not due to insertion or deletion of bases [127], [128]. Based on this they could identify repeating elements that are highly conserved across different species of *Pseudomonas* [128]. There are programs available for designing polymerase chain reaction (PCR) primer pairs as a means of rapid detection [129], [130]. These programs select primer pairs based on the user defined parameters. However, it becomes difficult to select the best primers since they do not provide information regarding the specificity of the oligonucleotides or patterns [131]–[134]. *B. cereus* has time and again been reported to be highly homogenous however, signature sequences of the 16S rDNA could distinguish the psychrotolerant and mesophilic strains. Single base pair substitutions were randomly distributed over the gene. The most obvious differences was one signature located at bp 180 to 192 (*E. coli* nomenclature) or bp 180 to 201 (*B. cereus* nomenclature) [127].

### Restriction Enzyme Analysis

The phenomenon of host specific restriction and modification of bacterial viruses stemmed from endonucleases within the cells that destroy foreign DNA molecules. REs cleave DNA at specific sites, generating discrete and gene-size fragments and have proved to be a remarkable tool for investigating gene organization, function and expression [135]–[137]. REs occur in combination with 1 or 2 modification enzymes (DNA methyl transferases) that protect the cell's own DNA from cleavage by the RE. Since ME methylates as the same site where RE cuts, R-M system perhaps ensures that 16S rDNA remains conserved in spite of the fact that a large number of sites for each RE are present. In other genes, presence of RE sites increases diversity by promoting recombination [138], [139]. PCR Restriction analysis (PRA) of 16S rDNA has been shown to contribute to rapidly and reliably identify newly isolated strains belonging to recognized species [140]. However as a result of analyzing a large set of data encompassing many species, we may extend the statement that this method can be applied for recognizing so far unrecognized species as well. In their study, they have applied four REs: *Hae*III, *Hin*fI, *Taq*I and *Rsa*I. However, *Hin*fI and *Rsa*I showed no distinctive patterns for the strains tested. In fact, *Taq*I proved instrumental in clearly distinguishing one of the groups. A wide range of REs such as *Hae*III, *Dpn*II, *Rsa*I, *Bfa*I and *Tru*9I were used for defining the genus *Virgibacillus* [141], [142]. *B. licheniformis* 16S rDNA sequence was spliced with *Alu*I into five bands −70; 140; 180; 200 and 800 nts, whereas *Rsa*I resulted in four bands −110; 400; 450; 500 nts [105].

An innovative approach for revealing intraspecific genomic variability of *B. cereus* and *B. licheniformis* was the PCR fingerprinting of the spaces between the 16S and 23S rRNA genes and of intergenic tRNA genes regions [60]. Although RAPD showed remarkable diversity among *B. cereus* strains however, it was realized that the genetic diversity can arise from plasmid wide variability in the plasmid profiles. *B. licheniformis* formed 2 groups with all the methods. Based on single strand conformation of polymorphism analysis after RE (*Alu*I and *Rsa*I) digestions of 16S rDNA, two different evolutionary schemes for the two *Bacillus* species, *B. cereus* and *B. licheniformis* were proposed [60]. In our analysis, only 6 out of 14 REs (*Dpn*II, *Rsa*I, *Bfa*I, *Hae*III, *Tru*9I and *Alu*I) - proved beneficial in easy distinction. The rest 8 REs (*Bam*HI, *Eco*RI, *Hin*dIII, *Not*I, *Nru*I, *Sac*I, *Sma*I, and *Pst*I) could not be exploited to significant extents to be useful for this purpose. However, in a different gene such as *gyr*B, digestion with *Eco*RI (and *Cla*I) could distinguish the four members of the *B. cereus* group but *Hin*dIII did not [38]. So the same set of REs may not hold good for different gene sequences. In fact *Hin*dIII gave poor results even while screening *Corynebacterium* spp., hence was discontinued and multiple RE usage was recommended [143]. *In silico* digestion with RE *Alu*I was found to be most discriminative [144] and generated 3 to 13 fragments depending on the *Mycoplasma* species. Although 73 *Mycoplasma* species could be differentiated using *Alu*I, other species gave undistinguishable patterns. For these, an additional restriction digestion typically with *Bfa*I (or hpyF10VI) was needed for a final identification [145]. This was confirmed by application of ARDRA on 27 species and subspecies. We also validated our findings by applying RE to species of *Virgibacillus*, *Gracibacillus* and *Geobacillus* and can be exploited for describing new species [146].

### Novel Lineages

Among the innovative strategies applied by various researchers, phylogenetic relationships between *Bacillus* species and related genera were inferred from comparison of 3′ end 16S rDNA and 5′ end 16S–23S ITS nucleotide sequences [82]. Among the 40 Bacillaceae species, *Bacillus circulans* remained ungrouped. Out of the ten groups, Group VI constituted of *B. licheniformis*, *B. subtilis*, *B. sphaericus* along with *B. amyloliquefaciens*, *B. atrophaecus*, *B. mojavensis*, *B. macroides* and *B. fusiformis*. Group X was placed independent of other 6 *Bacillus* groups and was comprised of *B. anthracis*, *B. cereus*, *B. thuringiensis*, *B. mycoides* and *B. lentus*. It indicates that *B. cereus* group is quite different from other *Bacillus* species. Separation of *Bacillus* species by *Paenibacillus*, *Brevibacillus*, *Geobacillus*, *Marinibacillus* and *Virgibacillus* species, indicates that in some cases, further divisions or conversely further grouping might be warranted. Our work has provided the tools for defining the thresholds of each species and enables us to pose the questions such as: Should current classifications be re-examined?

Lower levels of similarity were found with other alkalitolerant *Bacillus* strains particularly DSM8714 and DSM877, which still lack taxonomic standing. The results obtained confirm that the four *Eterogermina* strains belong to a unique genospecies which can be unequivocally identified as *B. clausii*. The finding is of intrinsic value, since some bacterial strains described as *B. clausii* strains have been reported to exhibit levels of DNA hybridization with the reference type strain of less than 61% [147]. Thus emphasizing the great genomic heterogeneity of the strains placed in the species, *B. clausii* [32].

As Ash et al., [42] predicted, their phylogenetic groups have been redefined as separate genera, and their outlying species have served as the basis for novel taxa as well. At the present pace of refined discoveries, we can expect new *Bacillus* - like genera to be redefined in the near future. In fact, Nazina et al., through physiological and genetic analysis submitted the validly described genus name of *Geobacillus* [65].

Heyndrickx et al., [142] undertook a polyphasic study, which revealed the presence within *Virgibacillus* of an as yet undescribed new species for which the name *Virgibacillus proomii* was proposed (*V. proomii* was distinguished from *V. pantothenticus* and members of *Bacillus sensu stricto* and from members of *Paenibacillus* and other aerobic endospore-forming bacteria by routine phenotypic tests). Comparisons of the 16S rDNA sequences of type strains of *Bacillus* and *Sporosarcina* species indicated that *Bacillus pantothenticus* lies at the periphery of rDNA gr1 (*Bacillus sensu stricto*) [42]. *Virgibacillus* was proposed to accommodate *B. pantothenticus* and two related organisms, which appeared to belong to an as yet (as on 1999) undescribed new species. It appears that the relationship between *Bacillus laevolecticus* and *V. pantothenticus* (Group III) and between *Bacillus badius* and *M. marinus* (Group IV) could still be open to debate. The robustness of this classification tool will be assessed by comparison with the current Bacillaceae classifications.

The various parameters like signatures (generated by MEME), restriction enzyme (RE) sites, nucleotide stretches “generated” by RE and the phylogenetic framework together can enable to generate a battery of markers. These are likely to define the variability between the species of a specific genus and the specificity of the genus. The use of these parameters is a simple, rapid approach, suitable to larger screening programs and easily accessible to most laboratories.

## Materials and Methods

### Sequence data

A total of 2146, 16S rDNA sequences belonging to the genus *Bacillus* (from RDP/NCBI sites: http://rdp.cme.msu.edu/; http://www.ncbi.nlm.nih.gov/) were analysed in the present study. These included 271 sequences belonging to isolates of *B. subtilis*, 211 to isolates of *B. cereus*, 153 to isolates of *B. anthracis*, 131 to isolates of *B. licheniformis*, 108 to isolates of *B. thuringiensis*, 83 to isolates of *B. pumilus*, 47 to isolates of *B. megaterium*, 42 to isolates of *B. sphaericus*, 39 to isolates of *B. clausii*, 36 to isolates of *B. halodurans* and 1025 to isolates of *Bacillus* species (Table 1). The first ten sets of *Bacillus* species were used as the master species set for this analysis for generating a phylogenetic framework while the 1025 *Bacillus* species were used as a data set for segregating these unclassified *Bacillus* species. The other *Bacillus* species, which were represented by relatively minor numbers, were not considered for the development of the identification tool (Table S5).

### Phylogenetic Analyses

For phylogenetic analyses of each of these 10 species data sets, the sequences from each of them were assembled and aligned using the multiple alignment program CLUSTALW version 1.82 [133]. To estimate evolutionary distance, pairwise distances between all taxa were calculated with the DNADIST of the PHYLIP 3.6 package. The resultant distance matrix was then used to draw a neighbor joining tree with the program NEIGHBOR. The program SEQBOOT [148] was used for statistical testing of the trees by resampling the dataset 1000 times. The trees were viewed through HyperTree Version 1.0.0 [133] and TreeView Version 1.6.6 [149] (Figures S1, S2, S3, S4, S5, S6, S7, S8, S9, S10).

For each of these 10 data sets, sequences which fell in the same clade were grouped together. Candidate sequences of these individual groups were aligned and a consensus was obtained by removing ambiguous parts using JALVIEW sequence editor. Consensus from each group was chosen as a representative for the particular group. A phylogenetic tree was drawn on the basis of these representative sequences of the 16S rDNA gene. Members from each of the clusters of the tree were selected to define the range of each of the *Bacillus* species. Thus a reference set of 34 sequences was selected that contained two to five from each represented topology (Table 5) and regarded these as likely candidates that could give information about the organismal phylogeny.

**Table 5:** Accession numbers of 16S rDNA sequences of *Bacillus* species used for generating phylogenetic framework (http://rdp cme.msu.edu/).

Organism	Reference sequence(s)
*Bacillus thuringiensis*	DQ286308(T)^a^, DQ286339, DQ328630, AE017355, DQ286329
*B. anthracis* b	AB190218, AE017334, AE017225
*B. cereus* b	DQ372919, DQ289988
*B. subtilis*	AB042061(T), DQ420172, AY995569, DQ504376, AY583216, AY881635, AY631853
*B. licheniformis*	AB039328(T), CP000002, AF234855
*B. pumilus*	AY260861(T), AY876289, DQ523500
*B. megaterium*	AJ717381(T), AY373358, AY505510, AY373360
*B. sphaericus*	AJ310084(T), DQ286309
*B. clausii*	X76440(T), AB201793, AY960116
*B. halodurans*	AY423275(T), AY856452
**Total**	**34 strains**

a Type strain.

b For *B. cereus* group only one type strain was used.

### *Bacillus* Species-Specific Signature

MEME (Multiple EM for Motif Elicitation) is used for searching for novel motifs or signatures in sets of biological sequences. MEME works by searching for repeated, ungapped sequence patterns that occur in the DNA or protein sequences [150], [151]. MEME searches can be performed via the web server (http://meme.nbcr.net) and its mirror sites [151]. The same web server also allows access to motif alignment and search tool to search sequence databases for matches to motifs. To successfully discover motifs with MEME, it is necessary to choose and prepare the input sequences carefully. Ideally, the sequences should be <1000 base pairs long [152]. In our analysis, sequences of 10 data sets in FASTA format were submitted group wise in MEME program Version 4.0.0 (http://meme.nbcr.net/meme4/cgi-bin/meme.cgi). In order to obtain maximum number of motifs in our sequences, we modified default settings from 3 motifs to 10 motifs. MEME search stops when this number of motifs has been found, or when none can be found with E-value less than 10000 (http://meme.nbcr.net/meme4/meme-input.html#width). We used default setting zero or one motif per sequence to get the occurrence of single motif which is distributed among the sequences. The default value of motif widths, set between 6 (minimum) and 50 (maximum) were modified and re-set between 25 and 30, respectively. Each of the 10 signatures (25 to 30 nucleotides long) (Table S2) was checked for its frequency of occurrence among all the sequences of a particular *Bacillus* sp. and the ones with highest frequency and did not appear in other *Bacillus* spp. were considered as unique to this species. These unique motifs were used as query sequence to BLAST against the sequenced microbial genomes available in NCBI database (http://www.ncbi.nlm.nih.gov/), to validate the results.

### Restriction Enzyme Analysis

A total of 14 Type II Restriction enzymes (Table 3) were considered for these analyses. The criteria for selecting type II RE are independence of methylase and occurrence of cleavage(s) at very specific sites that are within or close to the recognition sequence.

All the 10 *Bacillus* species considered were checked for all the 14 Type II RE using the online software: Restriction Mapper Version 3 (http://restrictionmapper.org/index.htm). Sequences (one at a time) were entered in the restriction mapper site, results obtained were analyzed and consensus pattern was determined for each species depending upon its frequency of occurrence in the sequences. Ten known *Bacillus* spp. and 11 clusters belonging to *Bacillus* sp. were used as data sets. For *B. megaterium*, *B. sphaericus*, *B. clausii*, *B. halodurans* all the sequences were analysed and for *B. cereus*, *B. anthracis*, *B. licheniformis*, *B. thuringiensis*, *B. pumilus* 30 sequences were taken into consideration but for *B. subtilis* since no conclusive pattern could be made using 30 sequences, so 150 sequences were taken. The patterns developed for each *Bacillus* species were considered as a representative for that specific species.

### Identifying *Bacillus* Species

The reference set of 34 sequences has been used to segregate unclassified *Bacillus* species. A total of 1025 16S rDNA sequences from *Bacillus* sp. were screened at the rate of 52 *Bacillus* species with 34 reference sequences by generating 22 phylogenetic trees (Figures S11, S12, S13, S14, S15, S16, S17, S18, S19, S20, S21, S22, S23, S24, S25, S26, S27, S28, S29, S30, S31, S32). In each of the phylogenetic tree, bootstrap percentage values that showed a stability of at least 80% were considered. These *Bacillus* species were then taken to reconstruct final phylogenetic tree with their respective species. These strains were also checked for the *Bacillus* specifies-specific signatures (similarly as done for 10 data sets) and restriction enzyme analysis as benchmark for concluding that the strain belonged to the specific *Bacillus* species.

### Clusters/Potential Novel Lineages

In addition to the identification of some *Bacillus* spp. up to species level, [366 sequences were aligned {according to clusters made in 22 trees of *Bacillus* sp.} and 52 representatives were chosen and a phylogenetic tree was made] 335 isolates were found to cluster in to 11 groups {44 representatives out of 52} (Figure 8). These 11 clusters consisting of 18 to 50 isolates each did not appear to fall within any of the aligned species and so may represent sub-species or novel lineages. These were also checked for signatures identified through MEME program to reveal that if there are certain similarities. Each of the 11 clusters was also checked for the 14 type II restriction enzyme digestion. The pattern so obtained was checked against those of the representative *Bacillus* species for a match so that a conclusion could be drawn that these might belong to some *Bacillus* species.

## Supporting Information

Table S1 Accession numbers of 16S rDNA sequences of Bacillus sp. identified up to species
(0.05 MB DOC)

Table S2 Signatures of Bacillus species obtained through MEME software (http://meme.sdsc.edu/meme/meme.html)
(0.10 MB DOC)

Table S3 Characteristics of nucleotide signatures for 16S rDNA gene of clusters of Bacillus sp.
(0.05 MB DOC)

Table S4 Occurrence of restriction endonuclease digestion sites in 16S rDNA sequence(s) of Bacillus spp. and clusters of Bacillus sp. with low frequency or limited RE sites.
(0.03 MB DOC)

Table S5 List of Bacillus species available at http://rdp.cme.msu.edu/.
(0.19 MB DOC)

Figure S1 Phylogenetic tree based on 153, 16S rRNA gene sequences of Bacillus anthracis. A neighbor-joining analysis with Jukes-Cantor correction and bootstrap support was performed on the gene sequences. Bootstrap values are given at nodes. Bold sequences are the ones considered as framework in the study. Values in parentheses are accession numbers (http://rdp.cme.msu.edu/).
(7.75 MB TIF)

Figure S2 Phylogenetic tree based on 211, 16S rRNA gene sequences of Bacillus cereus. A neighbor-joining analysis with Jukes-Cantor correction and bootstrap support was performed on the gene sequences. Bootstrap values are given at nodes. Bold sequences are the ones considered as framework in the study. Values in parentheses are accession numbers (http://rdp.cme.msu.edu/).
(2.16 MB TIF)

Figure S3 Phylogenetic tree based on 108, 16S rRNA gene sequences of Bacillus thuringiensis. A neighbor-joining analysis with Jukes-Cantor correction and bootstrap support was performed on the gene sequences. Bootstrap values are given at nodes. Bold sequences are the ones considered as framework in the study. Values in parentheses are accession numbers (http://rdp.cme.msu.edu/).
(1.52 MB TIF)

Figure S4 Phylogenetic tree based on 271, 16S rRNA gene sequences of Bacillus subtilis. A neighbor-joining analysis with Jukes-Cantor correction and bootstrap support was performed on the gene sequences. Bootstrap values are given at nodes. Bold sequences are the ones considered as framework in the study. Values in parentheses are accession numbers (http://rdp.cme.msu.edu/).
(10.12 MB TIF)

Figure S5 Phylogenetic tree based on 131, 16S rRNA gene sequences of Bacillus licheniformis. A neighbor-joining analysis with Jukes-Cantor correction and bootstrap support was performed on the gene sequences. Bootstrap values are given at nodes. Bold sequences are the ones considered as framework in the study. Values in parentheses are accession numbers (http://rdp.cme.msu.edu/).
(1.63 MB TIF)

Figure S6 Phylogenetic tree based on 83, 16S rRNA gene sequences of Bacillus pumilus. A neighbor-joining analysis with Jukes-Cantor correction and bootstrap support was performed on the gene sequences. Bootstrap values are given at nodes. Bold sequences are the ones considered as framework in the study. Values in parentheses are accession numbers (http://rdp.cme.msu.edu/).
(7.14 MB TIF)

Figure S7 Phylogenetic tree based on 42, 16S rRNA gene sequences of Bacillus sphaericus. A neighbor-joining analysis with Jukes-Cantor correction and bootstrap support was performed on the gene sequences. Bootstrap values are given at nodes. Bold sequences are the ones considered as framework in the study. Values in parentheses are accession numbers (http://rdp.cme.msu.edu/).
(0.75 MB TIF)

Figure S8 Phylogenetic tree based on 36, 16S rRNA gene sequences of Bacillus halodurans. A neighbor-joining analysis with Jukes-Cantor correction and bootstrap support was performed on the gene sequences. Bootstrap values are given at nodes. Bold sequences are the ones considered as framework in the study. Values in parentheses are accession numbers (http://rdp.cme.msu.edu/).
(0.87 MB TIF)

Figure S9 Phylogenetic tree based on 39, 16S rRNA gene sequences of Bacillus clausii. A neighbor-joining analysis with Jukes-Cantor correction and bootstrap support was performed on the gene sequences. Bootstrap values are given at nodes. Bold sequences are the ones considered as framework in the study. Values in parentheses are accession numbers (http://rdp.cme.msu.edu/).
(0.89 MB TIF)

Figure S10 Phylogenetic tree based on 47, 16S rRNA gene sequences of Bacillus megaterium. A neighbor-joining analysis with Jukes-Cantor correction and bootstrap support was performed on the gene sequences. Bootstrap values are given at nodes. Bold sequences are the ones considered as framework in the study. Values in parentheses are accession numbers (http://rdp.cme.msu.edu/).
(0.88 MB TIF)

Figure S11 Phylogenetic tree of 34 framework sequences (bold values) and Bacillus sp. at the rate of 52 sequences (1–1136 {including 1025 Bacillus sp. and rest other species to add authenticity to the results}) which could be designated as 10 known Bacillus spp. used in the study. A neighbor-joining analysis with Jukes-Cantor correction and bootstrap support was performed on the gene sequences. Bootstrap values are given at nodes. Values in parentheses are accession numbers (http://rdp.cme.msu.edu/).
(3.62 MB TIF)

Figure S12 Phylogenetic tree of 34 framework sequences (bold values) and Bacillus sp. at the rate of 52 sequences (1–1136 {including 1025 Bacillus sp. and rest other species to add authenticity to the results}) which could be designated as 10 known Bacillus spp. used in the study. A neighbor-joining analysis with Jukes-Cantor correction and bootstrap support was performed on the gene sequences. Bootstrap values are given at nodes. Values in parentheses are accession numbers (http://rdp.cme.msu.edu/).
(0.74 MB TIF)

Figure S13 Phylogenetic tree of 34 framework sequences (bold values) and Bacillus sp. at the rate of 52 sequences (1–1136 {including 1025 Bacillus sp. and rest other species to add authenticity to the results}) which could be designated as 10 known Bacillus spp. used in the study. A neighbor-joining analysis with Jukes-Cantor correction and bootstrap support was performed on the gene sequences. Bootstrap values are given at nodes. Values in parentheses are accession numbers (http://rdp.cme.msu.edu/).
(0.37 MB TIF)

Figure S14 Phylogenetic tree of 34 framework sequences (bold values) and Bacillus sp. at the rate of 52 sequences (1–1136 {including 1025 Bacillus sp. and rest other species to add authenticity to the results}) which could be designated as 10 known Bacillus spp. used in the study. A neighbor-joining analysis with Jukes-Cantor correction and bootstrap support was performed on the gene sequences. Bootstrap values are given at nodes. Values in parentheses are accession numbers (http://rdp.cme.msu.edu/).
(0.26 MB TIF)

Figure S15 Phylogenetic tree of 34 framework sequences (bold values) and Bacillus sp. at the rate of 52 sequences (1–1136 {including 1025 Bacillus sp. and rest other species to add authenticity to the results}) which could be designated as 10 known Bacillus spp. used in the study. A neighbor-joining analysis with Jukes-Cantor correction and bootstrap support was performed on the gene sequences. Bootstrap values are given at nodes. Values in parentheses are accession numbers (http://rdp.cme.msu.edu/).
(0.28 MB TIF)

Figure S16 Phylogenetic tree of 34 framework sequences (bold values) and Bacillus sp. at the rate of 52 sequences (1–1136 {including 1025 Bacillus sp. and rest other species to add authenticity to the results}) which could be designated as 10 known Bacillus spp. used in the study. A neighbor-joining analysis with Jukes-Cantor correction and bootstrap support was performed on the gene sequences. Bootstrap values are given at nodes. Values in parentheses are accession numbers (http://rdp.cme.msu.edu/).
(0.29 MB TIF)

Figure S17 Phylogenetic tree of 34 framework sequences (bold values) and Bacillus sp. at the rate of 52 sequences (1–1136 {including 1025 Bacillus sp. and rest other species to add authenticity to the results}) which could be designated as 10 known Bacillus spp. used in the study. A neighbor-joining analysis with Jukes-Cantor correction and bootstrap support was performed on the gene sequences. Bootstrap values are given at nodes. Values in parentheses are accession numbers (http://rdp.cme.msu.edu/).
(0.22 MB TIF)

Figure S18 Phylogenetic tree of 34 framework sequences (bold values) and Bacillus sp. at the rate of 52 sequences (1–1136 {including 1025 Bacillus sp. and rest other species to add authenticity to the results}) which could be designated as 10 known Bacillus spp. used in the study. A neighbor-joining analysis with Jukes-Cantor correction and bootstrap support was performed on the gene sequences. Bootstrap values are given at nodes. Values in parentheses are accession numbers (http://rdp.cme.msu.edu/).
(0.25 MB TIF)

Figure S19 Phylogenetic tree of 34 framework sequences (bold values) and Bacillus sp. at the rate of 52 sequences (1–1136 {including 1025 Bacillus sp. and rest other species to add authenticity to the results}) which could be designated as 10 known Bacillus spp. used in the study. A neighbor-joining analysis with Jukes-Cantor correction and bootstrap support was performed on the gene sequences. Bootstrap values are given at nodes. Values in parentheses are accession numbers (http://rdp.cme.msu.edu/).
(0.67 MB TIF)

Figure S20 Phylogenetic tree of 34 framework sequences (bold values) and Bacillus sp. at the rate of 52 sequences (1–1136 {including 1025 Bacillus sp. and rest other species to add authenticity to the results}) which could be designated as 10 known Bacillus spp. used in the study. A neighbor-joining analysis with Jukes-Cantor correction and bootstrap support was performed on the gene sequences. Bootstrap values are given at nodes. Values in parentheses are accession numbers (http://rdp.cme.msu.edu/).
(0.26 MB TIF)

Figure S21 Phylogenetic tree of 34 framework sequences (bold values) and Bacillus sp. at the rate of 52 sequences (1–1136 {including 1025 Bacillus sp. and rest other species to add authenticity to the results}) which could be designated as 10 known Bacillus spp. used in the study. A neighbor-joining analysis with Jukes-Cantor correction and bootstrap support was performed on the gene sequences. Bootstrap values are given at nodes. Values in parentheses are accession numbers (http://rdp.cme.msu.edu/).
(0.25 MB TIF)

Figure S22 Phylogenetic tree of 34 framework sequences (bold values) and Bacillus sp. at the rate of 52 sequences (1–1136 {including 1025 Bacillus sp. and rest other species to add authenticity to the results}) which could be designated as 10 known Bacillus spp. used in the study. A neighbor-joining analysis with Jukes-Cantor correction and bootstrap support was performed on the gene sequences. Bootstrap values are given at nodes. Values in parentheses are accession numbers (http://rdp.cme.msu.edu/).
(0.24 MB TIF)

Figure S23 Phylogenetic tree of 34 framework sequences (bold values) and Bacillus sp. at the rate of 52 sequences (1–1136 {including 1025 Bacillus sp. and rest other species to add authenticity to the results}) which could be designated as 10 known Bacillus spp. used in the study. A neighbor-joining analysis with Jukes-Cantor correction and bootstrap support was performed on the gene sequences. Bootstrap values are given at nodes. Values in parentheses are accession numbers (http://rdp.cme.msu.edu/).
(0.24 MB TIF)

Figure S24 Phylogenetic tree of 34 framework sequences (bold values) and Bacillus sp. at the rate of 52 sequences (1–1136 {including 1025 Bacillus sp. and rest other species to add authenticity to the results}) which could be designated as 10 known Bacillus spp. used in the study. A neighbor-joining analysis with Jukes-Cantor correction and bootstrap support was performed on the gene sequences. Bootstrap values are given at nodes. Values in parentheses are accession numbers (http://rdp.cme.msu.edu/).
(0.25 MB TIF)

Figure S25 Phylogenetic tree of 34 framework sequences (bold values) and Bacillus sp. at the rate of 52 sequences (1–1136 {including 1025 Bacillus sp. and rest other species to add authenticity to the results}) which could be designated as 10 known Bacillus spp. used in the study. A neighbor-joining analysis with Jukes-Cantor correction and bootstrap support was performed on the gene sequences. Bootstrap values are given at nodes. Values in parentheses are accession numbers (http://rdp.cme.msu.edu/).
(0.25 MB TIF)

Figure S26 Phylogenetic tree of 34 framework sequences (bold values) and Bacillus sp. at the rate of 52 sequences (1–1136 {including 1025 Bacillus sp. and rest other species to add authenticity to the results}) which could be designated as 10 known Bacillus spp. used in the study. A neighbor-joining analysis with Jukes-Cantor correction and bootstrap support was performed on the gene sequences. Bootstrap values are given at nodes. Values in parentheses are accession numbers (http://rdp.cme.msu.edu/).
(0.26 MB TIF)

Figure S27 Phylogenetic tree of 34 framework sequences (bold values) and Bacillus sp. at the rate of 52 sequences (1–1136 {including 1025 Bacillus sp. and rest other species to add authenticity to the results}) which could be designated as 10 known Bacillus spp. used in the study. A neighbor-joining analysis with Jukes-Cantor correction and bootstrap support was performed on the gene sequences. Bootstrap values are given at nodes. Values in parentheses are accession numbers (http://rdp.cme.msu.edu/).
(0.21 MB TIF)

Figure S28 Phylogenetic tree of 34 framework sequences (bold values) and Bacillus sp. at the rate of 52 sequences (1–1136 {including 1025 Bacillus sp. and rest other species to add authenticity to the results}) which could be designated as 10 known Bacillus spp. used in the study. A neighbor-joining analysis with Jukes-Cantor correction and bootstrap support was performed on the gene sequences. Bootstrap values are given at nodes. Values in parentheses are accession numbers (http://rdp.cme.msu.edu/).
(0.21 MB TIF)

Figure S29 Phylogenetic tree of 34 framework sequences (bold values) and Bacillus sp. at the rate of 52 sequences (1–1136 {including 1025 Bacillus sp. and rest other species to add authenticity to the results}) which could be designated as 10 known Bacillus spp. used in the study. A neighbor-joining analysis with Jukes-Cantor correction and bootstrap support was performed on the gene sequences. Bootstrap values are given at nodes. Values in parentheses are accession numbers (http://rdp.cme.msu.edu/).
(0.63 MB TIF)

Figure S30 Phylogenetic tree of 34 framework sequences (bold values) and Bacillus sp. at the rate of 52 sequences (1–1136 {including 1025 Bacillus sp. and rest other species to add authenticity to the results}) which could be designated as 10 known Bacillus spp. used in the study. A neighbor-joining analysis with Jukes-Cantor correction and bootstrap support was performed on the gene sequences. Bootstrap values are given at nodes. Values in parentheses are accession numbers (http://rdp.cme.msu.edu/).
(0.26 MB TIF)

Figure S31 Phylogenetic tree of 34 framework sequences (bold values) and Bacillus sp. at the rate of 52 sequences (1–1136 {including 1025 Bacillus sp. and rest other species to add authenticity to the results}) which could be designated as 10 known Bacillus spp. used in the study. A neighbor-joining analysis with Jukes-Cantor correction and bootstrap support was performed on the gene sequences. Bootstrap values are given at nodes. Values in parentheses are accession numbers (http://rdp.cme.msu.edu/).
(0.22 MB TIF)

Figure S32 Phylogenetic tree of 34 framework sequences (bold values) and Bacillus sp. at the rate of 52 sequences (1–1136 {including 1025 Bacillus sp. and rest other species to add authenticity to the results}) which could be designated as 10 known Bacillus spp. used in the study. A neighbor-joining analysis with Jukes-Cantor correction and bootstrap support was performed on the gene sequences. Bootstrap values are given at nodes. Values in parentheses are accession numbers (http://rdp.cme.msu.edu/).
(0.73 MB TIF)
