# Supplementary material for: Phylogeny in Aid of the Present and Novel Microbial Lineages: Diversity in Bacillus
Source: PLoS One. 2009 Feb 12;4(2):e4438. doi: 10.1371/journal.pone.0004438 (PMC2639701; doi:10.1371/journal.pone.0004438)
Supplement: Table S1 — Accession numbers of 16S rDNA sequences of Bacillus sp. identified up to species (0.05 MB DOC) [file pone.0004438.s001.doc]

**Table S1.** Accession numbers of 16S rDNA sequences of *Bacillus* sp. identified up to species level. (http://rdp.cme.msu.edu/)

| ***Bacillus sp.*** | | | | | | | |
| --- | --- | --- | --- | --- | --- | --- | --- |
| ***B. cereus*** | | | ***B. thuringiensis*** | ***B. anthracis*** | ***B. sphaericus*** | ***B. licheniformis*** | ***B. halodurans*** |
| AF350926  AF346493  Y15466  AF290562  AY189746  AB159768  AB159770  AY626835  AB114266  AF417874  AY669167  AY689058 AY689066 AY689080  AY690707  AY695834  AF227848  AF385082  AF441728  AF441732  AY387585  AY461742  AY461743  AY461744 AY461745 | AY461746  AY461747  AY461748  AY461749  AY461750  AY461752  AY461753  AY461754  AY461755  AY461756  AY461757  AY461758 AY461759  AY461790  AJ842963  AY756511  AY048782  AY048851  AY822614  AY572483  AY862988  AJ746156  AY954371  AY964603 AJ971887 | AJ971893  AY131217  DQ079058  DQ079060  AJ868359  DQ104987  DQ104988  DQ104990  DQ196480  DQ227781  DQ268872  DQ270682 DQ278903  DQ279393 DQ279399 DQ284450  DQ294345  DQ302160 DQ302161 DQ314542  AY853168 DQ374438  DQ376913 DQ376939 DQ376940 | AJ604543  DQ444982 | AF390088 | AB106346  AY082370  AF169492  AF169496  AF169497  AF169498  AF169500  AF169502  AF169503  AF169504  AF169505  AF169506  AF169510  AF169512  AY043358  AY787805  AY822613  DQ084534  AJ919999  AJ920000  DQ102370  DQ232748  DQ248040 | AB020199  AB020201  AB020195  X81131  AJ000648  AY553104  AY553105  AY553105  AB189316  AB189317  AJ831843  AJ831844  AY618580  AJ971851  AJ971873  DQ084465  DQ238044  DQ305285  DQ305286  DQ318779  DQ408588 | AB002661  DQ406675  AB043847  AB043844  AB043856  AY291122  X76442 |
| **75** | | | **02** | **01** | **23** | **21** | **07** |
| ***Bacillus sp.*** | | | | | | | |
| ***B. megaterium*** | | | ***B. pumilus*** | ***B. subtilis*** | | ***B. clausii*** | |
| AB066338  AB066347  AB066344  AB066339  AB066345  AJ236890  AY307367  AY621379  AY626828  AB114265  AB178889  AB118223  AJ748259  AJ784846  AJ784847  AY654897  AY654898  AY660697  AY660698  AY689061  AY690689  AF286480  AF332385 | AF427153  AY376876  AY289503  AY289508  AY764132  AY764133  AY785743  AJ315065  AJ315066  AJ316310  AJ316310  AY572486  AY788910  AY864631  AY864632  AY939830  AJ971877  AJ717382  AY131222  AB189702  AY965249  AY965250  AY965251 | AY965252  DQ105972  DQ166809  DQ180960  DQ192209  DQ270722  DQ270752  AY372926  AY822760  AY822763  DQ302416  DQ308409  DQ308410  DQ323079  DQ412062  AM237091  DQ448758  DQ453811  DQ485414  DQ444976  DQ497761  DQ512741  DQ523735 | DQ299293  AY505514  AY690700  AY690701  AB167393  AF142575  AF286483  AY177362  AY188840  D55731  AJ831842  AJ842964  AY723697  AJ315067  AJ867390  AY599743  AJ971852  AJ971880  DQ180946  DQ180947  DQ180948  DQ314544  DQ347558  DQ357789  DQ357791  DQ341425  DQ436343  DQ445268  DQ448760  DQ463425  DQ497762  DQ223659 | AM050346  AY188839  DQ451099  AY189745  AY189749  DQ451100  DQ243813  AB193437  AJ971872  AY741506  AF500205  AB159767  AB159769  AB055846  AB055848  AB188212  AY160223  AB053351  AY962472  AY601724  DQ278906  DQ278908 | DQ376935  AY319259  AY590138  AY485275  AY822759  DQ365582  DQ459329  AB050667  DQ377150  AB020193  DQ237947  DQ343614  AB098574  DQ394935  DQ314533  AY307364  DQ092500  AB017587  AF346494  DQ196479  DQ323077  DQ323078 | AJ507321  AB111932  AB111934  AY553128  AB035091  AB043839  AB043840  AB043841  AB043843  AB043848  AB043850  AB043853  AB043855  AB043859  AB098576  AF137020  AF142576  AY347311  AY751538  AY754340  AJ316316  AY829449  AY912087  AY914066  AY914067  AB120000  AB128830  AY793550  AJ717379  DQ298146  DQ363561 | |
| **69** | | | **32** | **44** | | **31** | |
| **Total 305** | | | | | | | |
