# Supplementary material for: Phylogeny in Aid of the Present and Novel Microbial Lineages: Diversity in Bacillus
Source: PLoS One. 2009 Feb 12;4(2):e4438. doi: 10.1371/journal.pone.0004438 (PMC2639701; doi:10.1371/journal.pone.0004438)
Supplement: Table S2 — Signatures of Bacillus species obtained through MEME software (http://meme.sdsc.edu/meme/meme.html) (0.10 MB DOC) [file pone.0004438.s002.doc]

**Table S2.** Signatures of *Bacillus* species obtained through MEME software (<http://meme.sdsc.edu/meme/meme.html>)

| *Bacillus spp.* | Signature (Nucleotides) | Signature number | Length (nts)^a^ | Freq.^b^ |
| --- | --- | --- | --- | --- |
| *B. anthracis* | TAATACCGGATAACATTTTGA  TTTCTTAAGTCTGATGTGAAA  TTCCACTTTCCTCTTCTGCA  ATGGATTAAGAGCTTGCTCTTATGAAGTTA  TCGTAAAACTCTGTTGTTAGGGAAGAACAA TTTAGTGCTGAAGTTAACGCATTAAGCACT TAATCTCATAAAACCGTTCTCAGTTCGGAT TAAACGATGAGTGCTAAGTGTTAGAGGGTT ATGTGGTTTAATTCGAAGCAACGCGAAGAA CAAGCGTTATCCGGAATTATTGGGCGTAAA | 1  2  3  4  5  6  7  8  9  10 | 21  21  20  30  30  30  30  30  30  30 | 107/153  149/153  00/153  37/153  148/153  49/153  107/153  116/153  14/153  41/153 |
| *B. cereus* | TAATACCGGATAACATTTTGA  TTTCTTAAGTCTGATGTGAAA  ATTAAGAGCTTGCTCTTATGA  TCGTAAAACTCTGTTGTTAGGGAAGAACAA TTTAGTGCTGAAGTTAACGCATTAAGCACT AACCCTTGATCTTAGTTGCCATCATTAAGT TAATCTCATAAAACCGTTCTCAGTTCGGAT AAAGTGGAATTCCATGTGTAGCGGTGAAAT TAAACGATGAGTGCTAAGTGTTAGAGGGTT ATGTGGTTTAATTCGAAGCAACGCGAAGAA | 1  2  3  4  5  6  7  8  9  10 | 21  21  21  30  30  30  30  30  30  30 | 106/211  164/211  116/211  150/211  136/211  50/211  98/211  151/211  120/211  67/211 |
| *B. thuringiensis* | ATAACATTTTGAACCGCATGGTTCGAAATT  ATAACATTTTGAACTGCATGGTTCGAAATT  AATGGATTAAGAGCTTGCTCTTATGAAGTT  TCGTAAAACTCTGTTGTTAGGGAAGAACAA  TTTAGTGCTGAAGTTAACGCATTAAGCACT  CTTTAGTGCTGAAGTTAACGCATTAAGCA  AACCCTTGATCTTAGTTGCCATCATTAAGT  TAATCTCATAAAACCGTTCTCAGTTCGGAT | 1  2  3  4, 5  6  7  8, 9  10 | 30  30  30  30  30  29  30  30 | 23/108  43/108  50/108  38/108  63/108  63/108  65/108  81/108 |
| *B. clausii* | ATGTGGTTTAATTCGAAGCAACGCGAAGAA AATCCCATAAAGCCATTCTCAGTTCGGATT AAATGATTGGGGTGAAGTCGTAACAAGGTA AAACCGGAGCTAATACCGGATAATCCCTTT GCATTAGCTAGTTGGTAAGGTAACGGCTTA GTAGTGCCGAAGTTAACACATTAAGCACT ATGACGTCAAATCATCATGCCCCTTATGA TACACACGTGCTACAATGGATGGTACAAA AAGCGTTGTCCGGAATTATTGGGCGTAAA AGGCGGCTTCTTAAGTCTGATGTGAAATCT | 1  2  3  4  5  6  7  8  9  10 | 30  30  30  30  30  29  29  29  29  30 | 04/39  14/39  31/39  14/39  15/39  23/39  17/39  37/39  18/39  33/39 |
| *B. halodurans* | ATAATAAAAAGAACTGCATGGTTCTTTTTT ACAATGGATGGTACAAAGGGTTGCGAAGCC CGAAGTTAACACATTAAGCACTCCGCCTG GGGAGCAAACAGGATTAGATACCCTGGTA GTGAGTGATGAAGGTTTTCGGATCGTAAA CCAGCATTCAGTTGGGCACTCTAAGGTGA ACCAAAGGGAGCTTGCTCCTAGAGGTTAGC TGTGGTTTAATTCGAAGCAACGCGAAGAA ACCGGGGAGGGTCATTGGAAACTGGGAGAC TCCGCAATGGACGAAAGTCTGACGGAGCAA | 1  2  3  4  5  6  7  8  9  10 | 30  30  29  29  29  29  30  29  30  30 | 21/36  34/36  15/36  15/36 22/36  21/36  01/36  15/36  16/36  15/36 |
| *B. licheniformis* | GTGCTTAATGCGTTTGCTGCAGCACTAAA TTGATTGAACCGCATGGTTCAATTATAAAA TAAAACTCTGTTGTTAGGGAAGAACAAGTA GCATGGTTCAATCATAAAAGGTGGCTTTTA TAAACGATGAGTGCTAAGTGTTAGAGGGTT ATGTGGTTTAATTCGAAGCAACGCGAAGAA TAGCGGTGAAATGCGTAGAGATGTGGAGGA ATGTGGTTTAATTCGAAGCAACGCGAAGAA | 1  2  3, 4  5  6, 7  8, 10  9  10 | 29  30  30  30  30  30  30  30 | 00/131  16/131  94/131  29/131  57/131  74/131  99/131  74/131 |
| *B. megaterium* | TAAAACTCTGTTGTTAGGGAAGAACAAGTA ATGATTGAAAGATGGTTTCGGCTATCACTT AATCCCATAAAACCATTCTCAGTTCGGATT AACTGATTAGAAGCTTGCTTCTATGACGTT AACTTGAGTGCAGAAGAGAAAAGCGGAATT TAAACGATGAGTGCTAAGTGTTAGAGGGTT ATGTGGTTTAATTCGAAGCAACGCGAAGAA AACCCTTGATCTTAGTTGCCAGCATTTAGT TCTTGACATCCTCTGACAACTCTAGAGATA TGGGATAACTTCGGGAAACCGAAGCTAATA | 1  2  3  4  5  6  7  8  9  10 | 30  30  30  30  30  30  30  30  30  30 | 37/47  36/47  19/47  28/47  22/47  26/47  22/47  10/47  33/47  34/47 |
| *B. sphaericus* | TAAAACTCTGTTGTAAGGGAAGAACAAGTA AACCCTTGATCTTAGTTGCCATCATTTAGT TTTAATTCGAAGCAACGCGAAGAACCTTA ATAGTGGAATTCCAAGTGTAGCGGTGAAAT TAATCCGATAAAGTCGTTCTCAGTTCGGAT AGTAACACGTGGGCAACCTACCTTATAGTT TAACTGGCTGTACCTTGACGGTACCTTATT CGCAGGTGGTTTCTTAAGTCTGATGTGAAA TAAACGATGAGTGCTAAGTGTTAGGGGGTT TACGGTCGCAAGACTGAAACTCAAAGGAAT | 1  2  3  4  5  6  7  8  9  10 | 30  30  29  30  30  30  30  30  30  30 | 23/42  25/42  24/42  14/42  23/42  10/42  27/42  19/42  29/42  29/42 |
| *B. subtilis* | TTGTTTGAACCGCATGGTTCAAACATAAAA  TTGTTTGAACCGCATGGTTCAGACATAAAA TAAAGCTCTGTTGTTAGGGAAGAACAAGTA  TAAAGCTCTGTTGTTAGGGAAGAACAAGT  TTGCTCCCTGATGTTAGCGGCGGACGGGTG | 1,2,3,5  4  6,7 ,9  8  10 | 30  30  30  29  30 | 70/211  19/211  122/211  179/211  86/211 |
| *B. pumilus* | TAAAGCTCTGTTGTTAGGGAAGAACAAGT  AAGGTTTAGCCAATCCCACAAATCTGTTCT  AAGGTTTAGCCAATCCCATAAATCTGTTCT  ATGAAGGTTTTCGGATCGTAAAGCTCTGTT  ATGTGGTTTAATTCGAAGCAACGCGAAGAA  TAGCGGTGAAATGCGTAGAGATGTGGAGGA  ATTGGAAACTGGGAAACTTGAGTGCAGAA AACCCTTGATCTTAGTTGCCAGCATTTAGT ATAGTTCCTTGAACCGCATGGTTCAAGGA | 1  2  3  4  5, 7  6  8  9  10 | 29  30  30  30  30  30  29  30  30 | 49/83  14/83  36/83  47/83  50/83  53/83  34/83  22/83  43/83 |

a: Nucleotides

b: Frequency of occurrence of the signature out of the total sequences screened.

A total of 10 signatures were deduced for each B*acillus* spp.

**Table S4.** Characteristics of nucleotide signatures for 16S rDNA gene of clusters of *Bacillus* sp.

| *Bacillus* spp. Cluster^a^ | Signature | Sign. No. | Length  (nts) ^b^ | Freq.^c^ |
| --- | --- | --- | --- | --- |
| Cluster 1  (46)^d^ | TAAAACTCTGTTGTAAGGGAAGAACAAGTA AATCCCATAAAACCGTTCCCAGTTCGGAT  AACCCTTGATCTTAGTTGCCATCATTTAGT  ATGTGGTTTAATTCGAAGCAACGCGAAGAA | 1,2,3,4  5  6,7,8  9,10 | 30  29  30  30 | 19  05  03  25 |
| Cluster 2  (29) | TAAAGCTCTGTTGTGAGGGAAGAACAAGTA ATGTGGTTTAATTCGAAGCAACGCGAAGAA  TAGCGGTGAAATGCGTAGATATGTGGAGGA TTTAATTCGAAGCAACGCGAAGAACCTTA AACCCTTGATCTTAGTTGCCAGCATTTAGT AATCCCATAAAGCCATTCTCAGTTCGGATT AAATGATTGGGGTGAAGTCGTAACAAGGTA | 1  2,3,4,6  5  7  8  9  10 | 30  30  30  29  30  30  30 | 06  09  11  07  01  02  04 |
| Cluster 3  (18) | TTTAATTCGAAGCAACGCGAAGAACCTTA TAAAGCTCTGTTGTTAGGGAAGAACAAGT  TAAAGCTCTGTTGTTAGGGAAGAACAAGTA AATGTTGAAAGTTGGCTTTCTGAGCTAACA  AACCCTTGATCTTAGTTGCCAGCATTCAGT AACCCTTGATCTTAGTTGCCAGCATTTAGT | 1,2,3  4  5,6  7  8,9  10 | 29  29  30  30  30  30 | 07  13  08  03  05  03 |
| Cluster 4  (22) | ATGAAGGCCTTCGGGTCGTAAAGTTCTGTT ATGAAGGTTTTCGGATCGTAAAACTCTGTT TCAAGCCAATCCCATAAAACCATTCTCAGT TTTAATTCGAAGCAACGCGAAGAACCTTA TTTAGCCAATCCCATAAAACCATTCTCAGT | 1  2,3,4  5  6,7,8  9,10 | 30  30  30  29  30 | 03  06  01  08  04 |
| Cluster 5  (32) | GATCTTCATTAGCTTGCTTTTGAAGATCA ATGAAGGCCTTCGGGTCGTAAAGCTCTGTT TGAGCGATGAAGGCCTTCGGGTCGTAAAG TAAGTGTTAGAGGGTTTCCGCCCTTTAGT ATGAAGGTTTTCGGATCGTAAAACTCTGTT AATCCCATAAATCTATTCTCAGTTCGGATT AATCCCATAAAACCATTCTCAGTTCGGATT | 1,2,3  4,5  6  7  8  9  10 | 29  30  29  29  30  30  30 | 15  07  06  21  04  05  07 |
| Cluster 6  (48) | ATAATACTTTTCATCACCTGATGAGAAGT GATAGTATTTCCTTTCTCCTGATTGGAAAT TAAGCAAATCCCATAAAACCATTCTCAGTT AAGCAAATCCCATAAAACCATTCTCAGT TCAAGCAAATCCCATAAAACCATTCTCAGT ATGAAGGCCTTCGGGTCGTAAAGCTCTGTT ATGAAGGTTTTCGGATCGTAAAACTCTGTT TTTAATTCGAAGCAACGCGAAGAACCTTA | 1  2  3  4  5,6  7  8  9,10 | 29  30  30  28  30  30  30  29 | 02  06  00  09  07  06  03  10 |
| Cluster 7  (37) | ATAACTCATTTCCTCGCATGAGGAAATGTT TTTAGCCAATCCCATAAAACCGTTCTCAGT TAAAACTCTGTTGTTAGGGAAGAACAAGT TAAAGCTCTGTTGTTAGGGAAGAACAAGT | 1  2,3,4  5,6,7  8,9,10 | 30  30  29  29 | 10  10  10  11 |
| Cluster 8  (28) | TAAAGTTCTGTTGTTAGGGAAGAACAAGTATAAAGCTCTGTTGTTAGGGAAGAACAAGTA  TAAAACTCTGTTGTTAGGGAAGAACAAGTATTTAATTCGAAGCAACGCGAAGAACCTTA  AACTTGAGTGCAGAAGAGGAAAGTGGAATT | 1,4  2  3  5,6,7,8  9,10 | 30  30  30  29  30 | 14  01  04  12  14 |
| Cluster 9  (25) | AAAGCTCTGTTGTTAGGGAAGAACAAGTA AATCCCATAAAACCATTCTCAGTTCGGATT TTTAATTCGAAGCAACGCGAAGAACCTTA AATCCCATAAAACCACTCTCAGTTCGGATT AATCCCATAAAGCCATTCTCAGTTCGGATT TAAAACTCTGTTGTTAGGGAAGAACAAGTA | 1  2,6  3,7,8,9  4  5  10 | 30  30  29  30  30  30 | 08  04  06  01  01  04 |
| Cluster 10 (25) | TTTAATTCGAAGCAACGCGAAGAACCTTA AATCCCACAAAACCGTTCCCAGTTCGGATT AATCCCACAAAACCATTCTCAGTTCGGATT AATCCCATAAAACCATTCTCAGTTCGGATT ATGACGTCAAATCATCATGCCCCTTATGA | 1,2,3,4  5  6  7  8,9,10 | 29  30  30  30  29 | 10  03  01  04  13 |
| Cluster 11 (50) | TAAACGATGAGTGCTAAGTGTTAGAGGGGT GTCGTAAAGCTCTGTTGTGAGGGACGAAGG ATGACGTCAAATCATCATGCCCCTTATGA | 1,2,7  3,4,5,6  8,9,10 | 30  30  29 | 23  25  13 |

a: Cluster represent isolates defined only up to genus level *Bacillus* sp.

b: Nucleotides

c: Frequency of occurrence of the signature out of the total 16S rDNA sequences screened.

d: Total number of 16S rDNA sequences screened.

**Table S5.** Occurrence of Restriction Endonuclease digestion sites in 16S rDNA Sequence(s) of *Bacillus* spp. and Clusters of *Bacillus* sp. with low frequency or limited RE sites.

|  | **Restriction Endonuclease** | | | | | | | | |
| --- | --- | --- | --- | --- | --- | --- | --- | --- | --- |
|  | NotI | SacI | BamHI | NruI | HindIII | | PstI | SmaI | EcoRI |
|  | ***Bacillus* spp.** | | | | | | | | |
| 16S rDNA Sequence(s) with RE site(s) (%)^a^ | 0.29 | 0.87 | 2.32 | 15.40 | 16.00 | | 41.00 | 95.9 | 97.38 |
| Organisms showing RE sites ( %)^b^ | NS^c^ | NS | NS | *B. subtilis* (23)  *B. sphaericus* (77) | *B. megaterium*  (78) | | *B. megaterium* (31)  *B. sphaericus* (25)  *B. lichemiformis* (21)  *B. pumilus* (20)  *B. subtilis* (3) | All species^d^ | All species |
|  | ***Bacillus* sp. Clusters 1 to 10** | | | | | | | | |
| 16S rDNA Sequence(s) with RE site(s) (%)^e^ | 0.88 | 0.44 | 3.07 | 14.47 | | 13.15 | 83.77 | 96.05 | 97.80 |
| No. of *Bacillus* sp. Clusters with RE sites | 2 | 1 | 3 | 4 | | 6 | 8 | 10 | 10 |

a: Based on a total of 344 sequences

b: Contribution of individual *Bacillus* spp. with respect to values in the previous row as 100%

c: Non Significant

d: Only one site per sequence was present.

e: Based on a total of 335 sequences
