# Supplementary material for: Phylogeny in Aid of the Present and Novel Microbial Lineages: Diversity in Bacillus
Source: PLoS One. 2009 Feb 12;4(2):e4438. doi: 10.1371/journal.pone.0004438 (PMC2639701; doi:10.1371/journal.pone.0004438)
Supplement: Table S3 — Characteristics of nucleotide signatures for 16S rDNA gene of clusters of Bacillus sp. (0.05 MB DOC) [file pone.0004438.s003.doc]

**Table S3.** Characteristics of nucleotide signatures for 16S rDNA gene of clusters of *Bacillus* sp.

| *Bacillus* spp. Cluster^a^ | Signature | Sign. No. | Length  (nts) ^b^ | Freq.^c^ |
| --- | --- | --- | --- | --- |
| Cluster 1  (46)^d^ | TAAAACTCTGTTGTAAGGGAAGAACAAGTA AATCCCATAAAACCGTTCCCAGTTCGGAT  AACCCTTGATCTTAGTTGCCATCATTTAGT  ATGTGGTTTAATTCGAAGCAACGCGAAGAA | 1,2,3,4  5  6,7,8  9,10 | 30  29  30  30 | 19  05  03  25 |
| Cluster 2  (29) | TAAAGCTCTGTTGTGAGGGAAGAACAAGTA ATGTGGTTTAATTCGAAGCAACGCGAAGAA  TAGCGGTGAAATGCGTAGATATGTGGAGGA TTTAATTCGAAGCAACGCGAAGAACCTTA AACCCTTGATCTTAGTTGCCAGCATTTAGT AATCCCATAAAGCCATTCTCAGTTCGGATT AAATGATTGGGGTGAAGTCGTAACAAGGTA | 1  2,3,4,6  5  7  8  9  10 | 30  30  30  29  30  30  30 | 06  09  11  07  01  02  04 |
| Cluster 3  (18) | TTTAATTCGAAGCAACGCGAAGAACCTTA TAAAGCTCTGTTGTTAGGGAAGAACAAGT  TAAAGCTCTGTTGTTAGGGAAGAACAAGTA AATGTTGAAAGTTGGCTTTCTGAGCTAACA  AACCCTTGATCTTAGTTGCCAGCATTCAGT AACCCTTGATCTTAGTTGCCAGCATTTAGT | 1,2,3  4  5,6  7  8,9  10 | 29  29  30  30  30  30 | 07  13  08  03  05  03 |
| Cluster 4  (22) | ATGAAGGCCTTCGGGTCGTAAAGTTCTGTT ATGAAGGTTTTCGGATCGTAAAACTCTGTT TCAAGCCAATCCCATAAAACCATTCTCAGT TTTAATTCGAAGCAACGCGAAGAACCTTA TTTAGCCAATCCCATAAAACCATTCTCAGT | 1  2,3,4  5  6,7,8  9,10 | 30  30  30  29  30 | 03  06  01  08  04 |
| Cluster 5  (32) | GATCTTCATTAGCTTGCTTTTGAAGATCA ATGAAGGCCTTCGGGTCGTAAAGCTCTGTT TGAGCGATGAAGGCCTTCGGGTCGTAAAG TAAGTGTTAGAGGGTTTCCGCCCTTTAGT ATGAAGGTTTTCGGATCGTAAAACTCTGTT AATCCCATAAATCTATTCTCAGTTCGGATT AATCCCATAAAACCATTCTCAGTTCGGATT | 1,2,3  4,5  6  7  8  9  10 | 29  30  29  29  30  30  30 | 15  07  06  21  04  05  07 |
| Cluster 6  (48) | ATAATACTTTTCATCACCTGATGAGAAGT GATAGTATTTCCTTTCTCCTGATTGGAAAT TAAGCAAATCCCATAAAACCATTCTCAGTT AAGCAAATCCCATAAAACCATTCTCAGT TCAAGCAAATCCCATAAAACCATTCTCAGT ATGAAGGCCTTCGGGTCGTAAAGCTCTGTT ATGAAGGTTTTCGGATCGTAAAACTCTGTT TTTAATTCGAAGCAACGCGAAGAACCTTA | 1  2  3  4  5,6  7  8  9,10 | 29  30  30  28  30  30  30  29 | 02  06  00  09  07  06  03  10 |
| Cluster 7  (37) | ATAACTCATTTCCTCGCATGAGGAAATGTT TTTAGCCAATCCCATAAAACCGTTCTCAGT TAAAACTCTGTTGTTAGGGAAGAACAAGT TAAAGCTCTGTTGTTAGGGAAGAACAAGT | 1  2,3,4  5,6,7  8,9,10 | 30  30  29  29 | 10  10  10  11 |
| Cluster 8  (28) | TAAAGTTCTGTTGTTAGGGAAGAACAAGTATAAAGCTCTGTTGTTAGGGAAGAACAAGTA  TAAAACTCTGTTGTTAGGGAAGAACAAGTATTTAATTCGAAGCAACGCGAAGAACCTTA  AACTTGAGTGCAGAAGAGGAAAGTGGAATT | 1,4  2  3  5,6,7,8  9,10 | 30  30  30  29  30 | 14  01  04  12  14 |
| Cluster 9  (25) | AAAGCTCTGTTGTTAGGGAAGAACAAGTA AATCCCATAAAACCATTCTCAGTTCGGATT TTTAATTCGAAGCAACGCGAAGAACCTTA AATCCCATAAAACCACTCTCAGTTCGGATT AATCCCATAAAGCCATTCTCAGTTCGGATT TAAAACTCTGTTGTTAGGGAAGAACAAGTA | 1  2,6  3,7,8,9  4  5  10 | 30  30  29  30  30  30 | 08  04  06  01  01  04 |
| Cluster 10 (25) | TTTAATTCGAAGCAACGCGAAGAACCTTA AATCCCACAAAACCGTTCCCAGTTCGGATT AATCCCACAAAACCATTCTCAGTTCGGATT AATCCCATAAAACCATTCTCAGTTCGGATT ATGACGTCAAATCATCATGCCCCTTATGA | 1,2,3,4  5  6  7  8,9,10 | 29  30  30  30  29 | 10  03  01  04  13 |
| Cluster 11 (50) | TAAACGATGAGTGCTAAGTGTTAGAGGGGT GTCGTAAAGCTCTGTTGTGAGGGACGAAGG ATGACGTCAAATCATCATGCCCCTTATGA | 1,2,7  3,4,5,6  8,9,10 | 30  30  29 | 23  25  13 |

a: Cluster represent isolates defined only up to genus level *Bacillus* sp.

b: Nucleotides

c: Frequency of occurrence of the signature out of the total 16S rDNA sequences screened.

d: Total number of 16S rDNA sequences screened.
