# Supplementary material for: Phylogeny in Aid of the Present and Novel Microbial Lineages: Diversity in Bacillus
Source: PLoS One. 2009 Feb 12;4(2):e4438. doi: 10.1371/journal.pone.0004438 (PMC2639701; doi:10.1371/journal.pone.0004438)
Supplement: Table S4 — Occurrence of restriction endonuclease digestion sites in 16S rDNA sequence(s) of Bacillus spp. and clusters of Bacillus sp. with low frequency or limited RE sites. (0.03 MB DOC) [file pone.0004438.s004.doc]

**Table S4.** Occurrence of Restriction Endonuclease digestion sites in 16S rDNA Sequence(s) of *Bacillus* spp. and Clusters of *Bacillus* sp. with low frequency or limited RE sites.

|  | **Restriction Endonuclease** | | | | | | | | |
| --- | --- | --- | --- | --- | --- | --- | --- | --- | --- |
|  | NotI | SacI | BamHI | NruI | HindIII | | PstI | SmaI | EcoRI |
|  | ***Bacillus* spp.** | | | | | | | | |
| 16S rDNA Sequence(s) with RE site(s) (%)^a^ | 0.29 | 0.87 | 2.32 | 15.40 | 16.00 | | 41.00 | 95.9 | 97.38 |
| Organisms showing RE sites ( %)^b^ | NS^c^ | NS | NS | *B. subtilis* (23)  *B. sphaericus* (77) | *B. megaterium*  (78) | | *B. megaterium* (31)  *B. sphaericus* (25)  *B. lichemiformis* (21)  *B. pumilus* (20)  *B. subtilis* (3) | All species^d^ | All species |
|  | ***Bacillus* sp. Clusters 1 to 10** | | | | | | | | |
| 16S rDNA Sequence(s) with RE site(s) (%)^e^ | 0.88 | 0.44 | 3.07 | 14.47 | | 13.15 | 83.77 | 96.05 | 97.80 |
| No. of *Bacillus* sp. Clusters with RE sites | 2 | 1 | 3 | 4 | | 6 | 8 | 10 | 10 |

a: Based on a total of 344 sequences

b: Contribution of individual *Bacillus* spp. with respect to values in the previous row as 100%

c: Non Significant

d: Only one site per sequence was present.

e: Based on a total of 335 sequences
