# Supplementary material for: Phylogeny in Aid of the Present and Novel Microbial Lineages: Diversity in Bacillus
Source: PLoS One. 2009 Feb 12;4(2):e4438. doi: 10.1371/journal.pone.0004438 (PMC2639701; doi:10.1371/journal.pone.0004438)
Supplement: Table S5 — List of Bacillus species available at http://rdp.cme.msu.edu/. (0.19 MB DOC) [file pone.0004438.s005.doc]

**Table S5.** List of *Bacillus* species available at http://rdp.cme.msu.edu/.

| S. No. | Organism | Number of sequences^a^ |
| --- | --- | --- |
|  | *Bacillus* sp*.* | 1025 |
|  | *B. subtilis* | 271 |
|  | *B. cereus* | 211 |
|  | *B. anthracis* str*.* | 153 |
|  | *B. licheniformis* | 131 |
|  | *B. thuringiensis* | 108 |
|  | *B. pumilus* | 83 |
|  | *B. megaterium* | 47 |
|  | *B. sphaericus* | 42 |
|  | *B. clausii* | 39 |
|  | *B. halodurans* | 36 |
|  | uncultured *Bacillus* sp*.* | 24 |
|  | *B. fusiformis* | 18 |
|  | *B. coagulans* | 17 |
|  | *B. firmus* | 14 |
|  | *B. simplex* | 14 |
|  | *B. mycoides* | 12 |
|  | *B. circulans* | 11 |
|  | *B. baekryungensis* | 10 |
|  | *B. macroides* | 10 |
|  | *B. sonorensis* | 9 |
|  | *B. flexus* | 8 |
|  | *B. fumarioli* | 7 |
|  | *B. niacini* | 7 |
|  | *B. amyloliquefaciens* | 6 |
|  | *B. aquimaris* | 6 |
|  | *B. benzoevorans* | 6 |
|  | *B. horikoshii* | 6 |
|  | *B. mojavensis* | 6 |
|  | *B. mucilaginosus* | 6 |
|  | *B. weihenstephanensis* | 6 |
|  | *B. badius* | 5 |
|  | *B. gelatini* | 5 |
|  | *B. gibsonii* | 5 |
|  | *B. niabensis* | 5 |
|  | *B. pseudofirmus* | 5 |
|  | *B. sporothermodurans* | 5 |
|  | *B. alcalophilus* | 4 |
|  | *B. bataviensis* | 4 |
|  | *B. borotolerans* | 4 |
|  | *B. caldotenax* | 4 |
|  | *B. cohnii* | 4 |
|  | *B. drentensis* | 4 |
|  | *B. farraginis* | 4 |
|  | *B. funiculus* | 4 |
|  | *B. lentus* | 4 |
|  | *B. methanolicus* | 4 |
|  | *B. pycnus* | 4 |
|  | *B. smithii* | 4 |
|  | *B. thermoamylovorans* | 4 |
|  | *B. agaradhaerens* | 3 |
|  | *B. algicola* | 3 |
|  | *B. atrophaeus* | 3 |
|  | *B. borophilicus* | 3 |
|  | *B. caldovelox* | 3 |
|  | *B. laevolacticus* | 3 |
|  | *B. litoralis* | 3 |
|  | *B. marisflavi* | 3 |
|  | *B. massiliensis* | 3 |
|  | *B. neidei* | 3 |
|  | *B. oleronius* | 3 |
|  | *B. pichinotyi* | 3 |
|  | *B. psychrosaccharolyticus* | 3 |
|  | *B. racemilacticus* | 3 |
|  | *B. senegalensis* | 3 |
|  | *B. soli* | 3 |
|  | *B. velezensis* | 3 |
|  | *B. acidicola* | 2 |
|  | *B. alveayuensis* | 2 |
|  | *B. arseniciselenatis* | 2 |
|  | *B. azotoformans* | 2 |
|  | *B. caldolyticus* | 2 |
|  | *B. carboniphilus* | 2 |
|  | *B. edaphicus* | 2 |
|  | *B. endophyticus* | 2 |
|  | *B. halmapalus* | 2 |
|  | *B. halophilus* | 2 |
|  | *B. horti* | 2 |
|  | *B. humi* | 2 |
|  | *B. infernus* | 2 |
|  | *B. jeotgali* | 2 |
|  | *B. novalis* | 2 |
|  | *B. okuhidensis* | 2 |
|  | *B. polyfermenticus* | 2 |
|  | *B. psychrodurans* | 2 |
|  | *B. schlegelii* | 2 |
|  | *B. silvestris* | 2 |
|  | *B. thermoterrestris* | 2 |
|  | *B. acidovorans* | 1 |
|  | *B. aeolius* | 1 |
|  | *B. aestuarii* | 1 |
|  | *B. akibai* | 1 |
|  | *B. alcaliinulinus* | 1 |
|  | *B. alkalitolerans* | 1 |
|  | *B. alkalogaya* | 1 |
|  | *B. amiliensis* | 1 |
|  | *B. aminovorans* | 1 |
|  | *B. arbutinivorans* | 1 |
|  | *B. arenosi* | 1 |
|  | *B. arsenicus* | 1 |
|  | *B. arvi* | 1 |
|  | *B. asahii* | 1 |
|  | *B. axarquiensis* | 1 |
|  | *B. barbaricus* | 1 |
|  | *B. bogoriensis* | 1 |
|  | *B. catenulatus* | 1 |
|  | *B. cellulosilyticus* | 1 |
|  | *B. cibi* | 1 |
|  | *B. clarkii* | 1 |
|  | *B. decolorationis* | 1 |
|  | *B. djibelorensis* | 1 |
|  | *B. fastidiosus* | 1 |
|  | *B. fordii* | 1 |
|  | *B. fortis* | 1 |
|  | *B. fucosivorans* | 1 |
|  | *B. galactosidilyticus* | 1 |
|  | *B. ginsenggisoli* | 1 |
|  | *B. ginsengihumi* | 1 |
|  | *B. granadensis* | 1 |
|  | *B. hackensackii* | 1 |
|  | *B. hemicellulosilyticus* | 1 |
|  | *B. herbersteinensis* | 1 |
|  | *B. hwajinpoensis* | 1 |
|  | *B. indicus* | 1 |
|  | *B. insolitus* | 1 |
|  | *B. koguryoae* | 1 |
|  | *B. koreensis* | 1 |
|  | *B. krulwichiae* | 1 |
|  | *B. longisporus* | 1 |
|  | *B. luciferensis* | 1 |
|  | *B. macauensis* | 1 |
|  | *B. macyae* | 1 |
|  | *B. malacitensis* | 1 |
|  | *B. mangrovensis* | 1 |
|  | *B. mannanilyticus* | 1 |
|  | *B. maroccanus* | 1 |
|  | *B. muralis* | 1 |
|  | *B. naganoensis* | 1 |
|  | *B. nealsonii* | 1 |
|  | *B. nematotocita* | 1 |
|  | *B. neonatiensis* | 1 |
|  | *B. nitritophilus* | 1 |
|  | *B. novalis* | 1 |
|  | *B. odysseyi* | 1 |
|  | *B. okhensis* | 1 |
|  | *B. olivae* | 1 |
|  | *B. oshimensis* | 1 |
|  | *B. panaciterrae* | 1 |
|  | *B. patagoniensis* | 1 |
|  | *B. pocheonensis* | 1 |
|  | *B. pseudalcaliphilus* | 1 |
|  | *B. pseudomegaterium* | 1 |
|  | *B. pseudomycoides* | 1 |
|  | *B. psychrotolerans* | 1 |
|  | *B. psychroviridis* | 1 |
|  | *B. ruris; type strain* | 1 |
|  | *B. salarius* | 1 |
|  | *B. saliphilus* | 1 |
|  | *B. selenatarsenatis* | 1 |
|  | *B. selenitireducens* | 1 |
|  | *B. shackletonii* | 1 |
|  | *B. siralis* | 1 |
|  | *B. solfatarensis* | 1 |
|  | *B. subterraneus* | 1 |
|  | *B. thermoalkalophilus* | 1 |
|  | *B. thermocloaceae* | 1 |
|  | *B. thermozeamaize* | 1 |
|  | *B. tipchiralis* | 1 |
|  | *B. vallismortis* | 1 |
|  | *B. vedderi* | 1 |
|  | *B. vietnamensis* | 1 |
|  | *B. vireti* | 1 |
|  | *B. viscosus* | 1 |
|  | *B. vortex* | 1 |
|  | *B. wakoensis* | 1 |
|  | **Total** | **2611** |

a: Collected from <http://rdp.cme.msu.edu/> .

Organisms are arranged in decreasing order of number of sequences present.
