# Supplementary figures and images for: Phylogeny in Aid of the Present and Novel Microbial Lineages: Diversity in Bacillus
Source: PLoS One. 2009 Feb 12;4(2):e4438. doi: 10.1371/journal.pone.0004438 (PMC2639701; doi:10.1371/journal.pone.0004438)

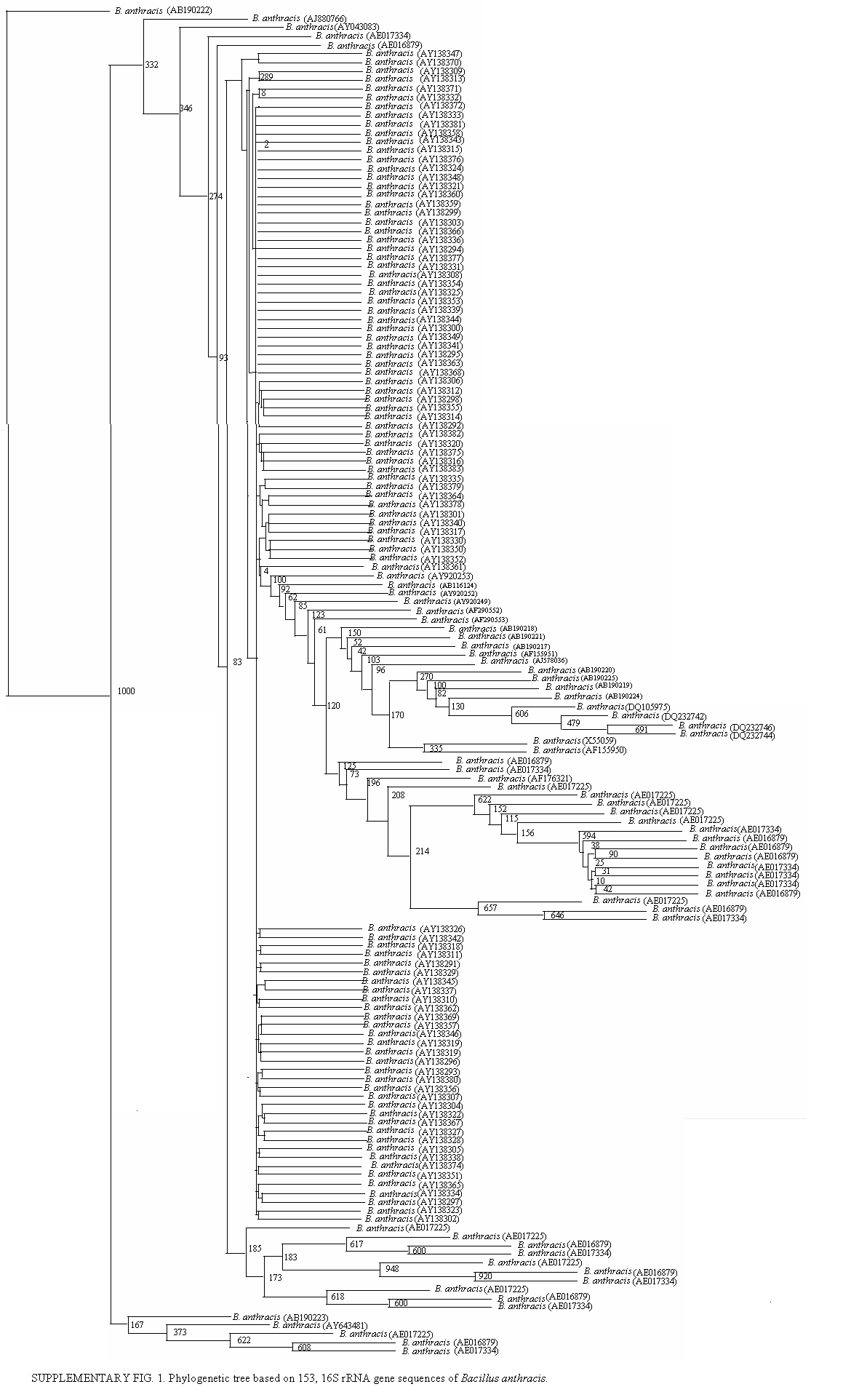

Supplement: Figure S1 — Phylogenetic tree based on 153, 16S rRNA gene sequences of Bacillus anthracis. A neighbor-joining analysis with Jukes-Cantor correction and bootstrap support was performed on the gene sequences. Bootstrap values are given at nodes. Bold sequences are the ones considered as framework in the study. Values in parentheses are accession numbers (http://rdp.cme.msu.edu/). (7.75 MB TIF) [file pone.0004438.s006.tif]

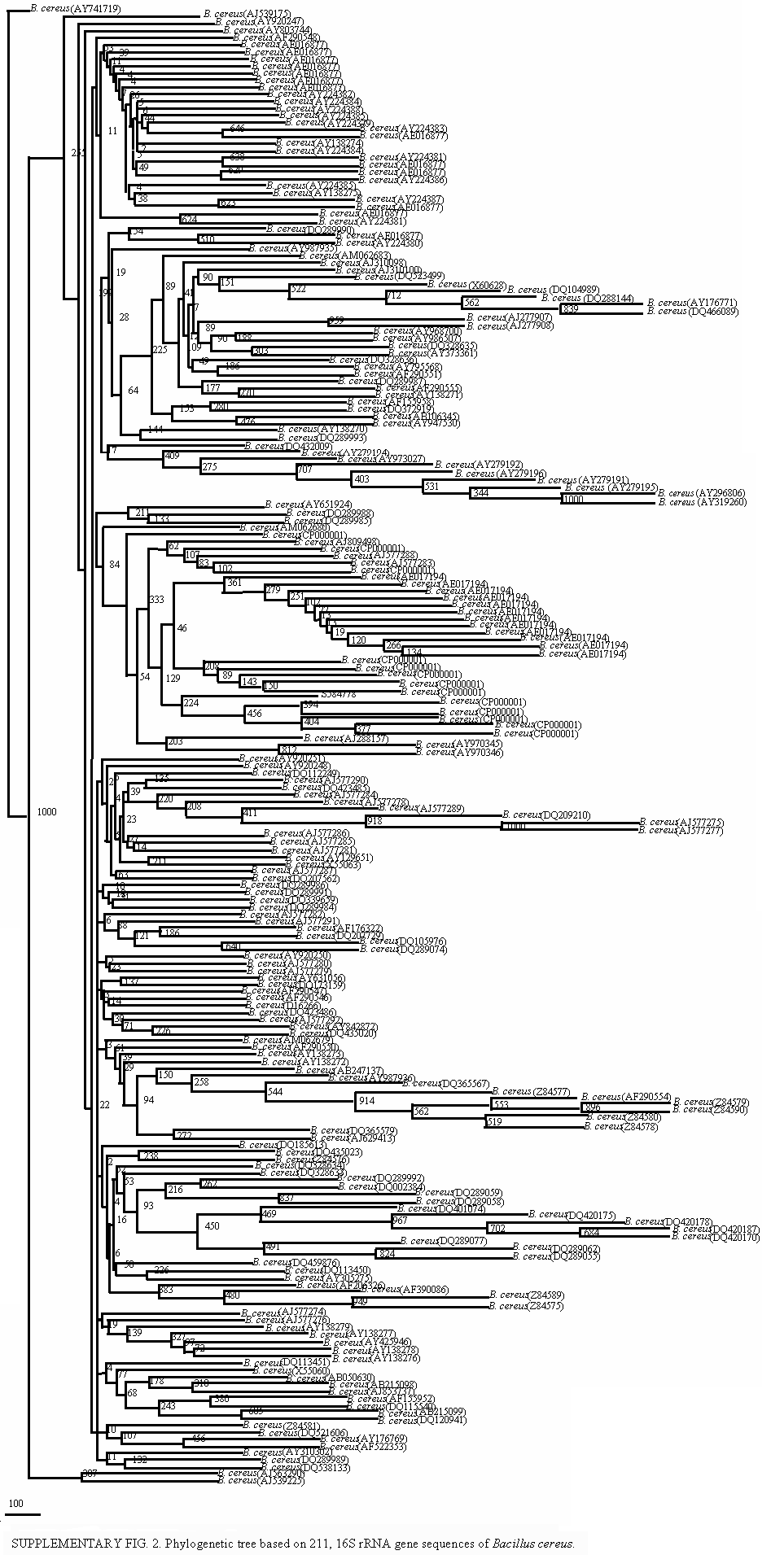

Supplement: Figure S2 — Phylogenetic tree based on 211, 16S rRNA gene sequences of Bacillus cereus. A neighbor-joining analysis with Jukes-Cantor correction and bootstrap support was performed on the gene sequences. Bootstrap values are given at nodes. Bold sequences are the ones considered as framework in the study. Values in parentheses are accession numbers (http://rdp.cme.msu.edu/). (2.16 MB TIF) [file pone.0004438.s007.tif]

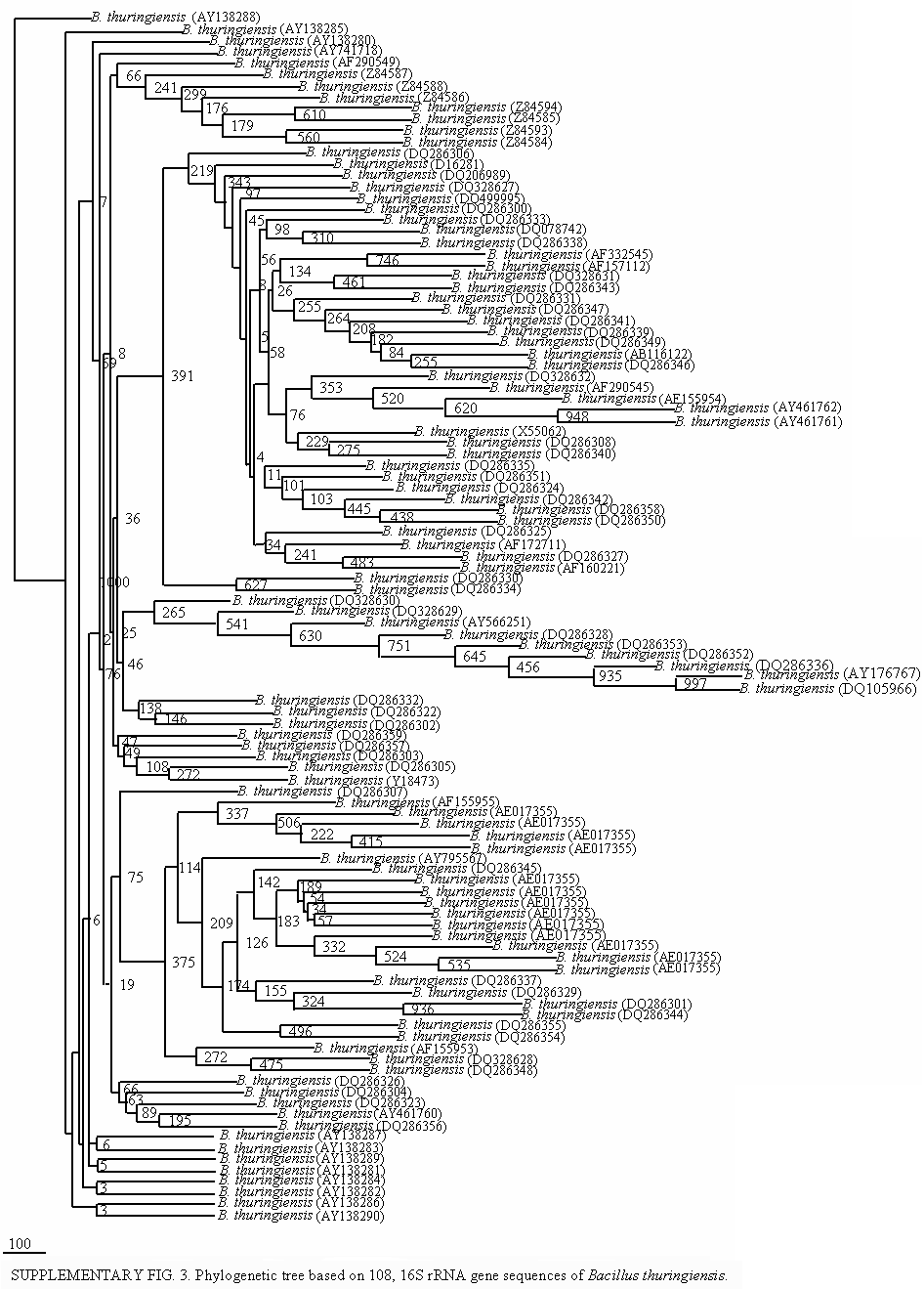

Supplement: Figure S3 — Phylogenetic tree based on 108, 16S rRNA gene sequences of Bacillus thuringiensis. A neighbor-joining analysis with Jukes-Cantor correction and bootstrap support was performed on the gene sequences. Bootstrap values are given at nodes. Bold sequences are the ones considered as framework in the study. Values in parentheses are accession numbers (http://rdp.cme.msu.edu/). (1.52 MB TIF) [file pone.0004438.s008.tif]

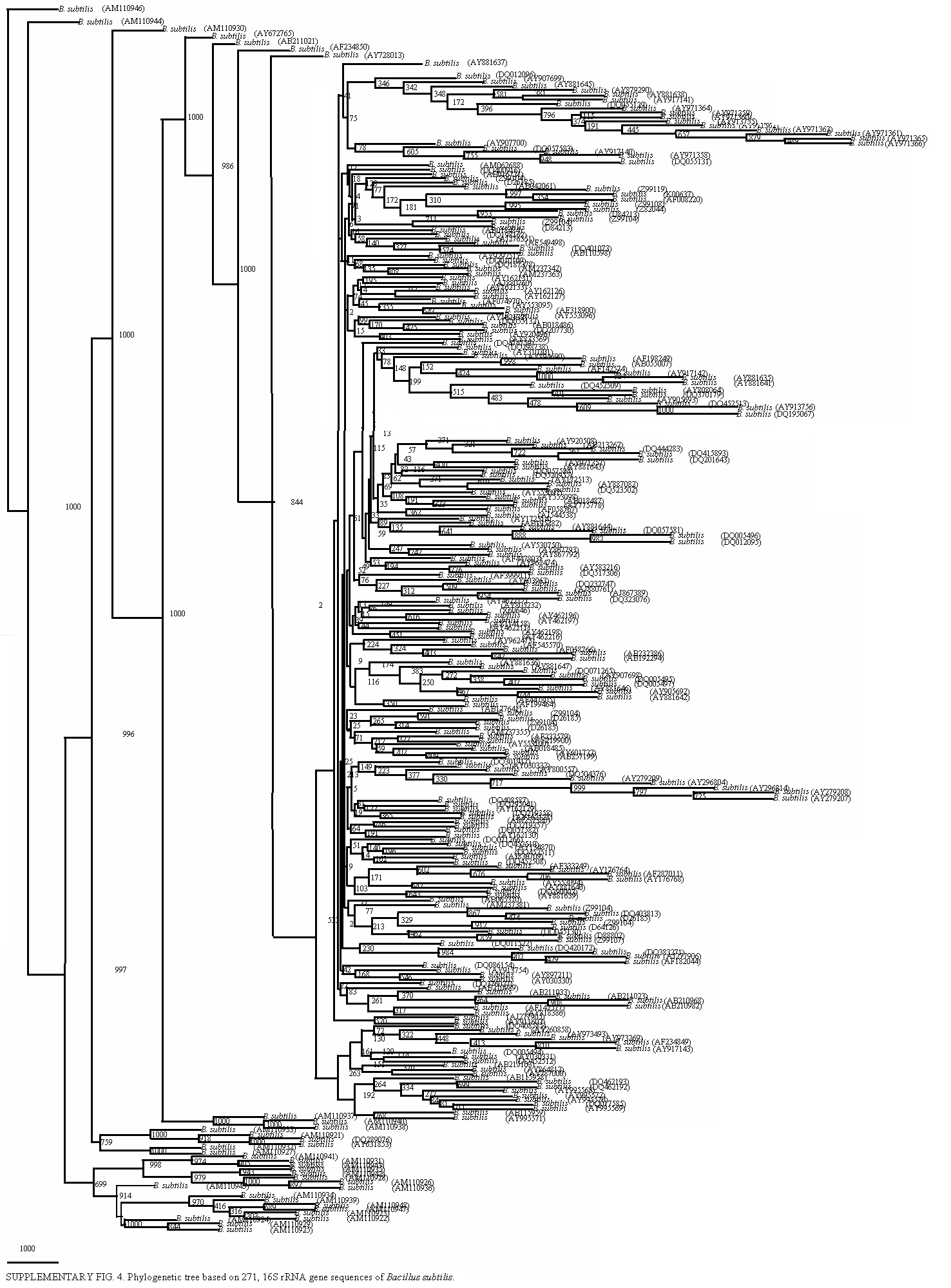

Supplement: Figure S4 — Phylogenetic tree based on 271, 16S rRNA gene sequences of Bacillus subtilis. A neighbor-joining analysis with Jukes-Cantor correction and bootstrap support was performed on the gene sequences. Bootstrap values are given at nodes. Bold sequences are the ones considered as framework in the study. Values in parentheses are accession numbers (http://rdp.cme.msu.edu/). (10.12 MB TIF) [file pone.0004438.s009.tif]

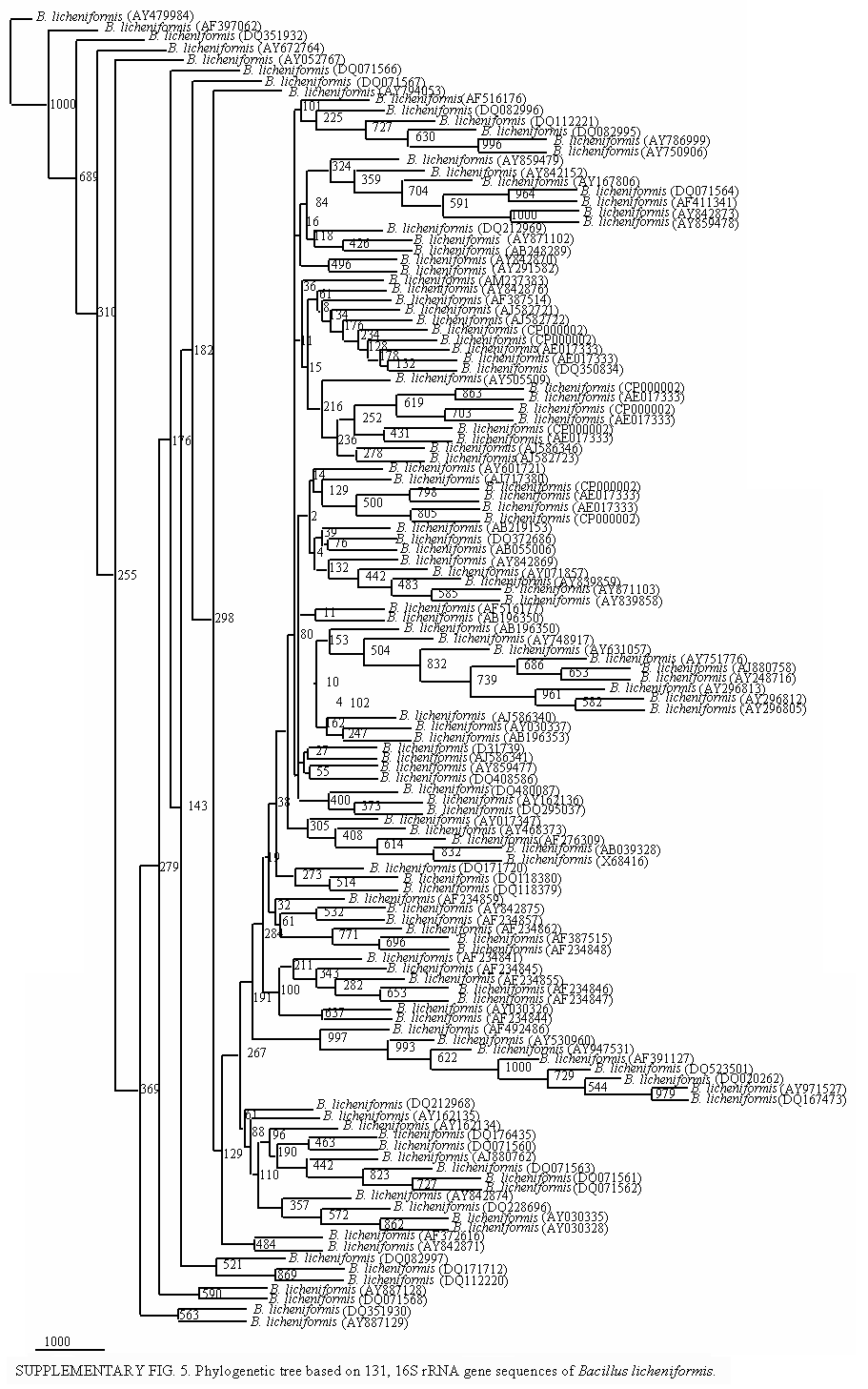

Supplement: Figure S5 — Phylogenetic tree based on 131, 16S rRNA gene sequences of Bacillus licheniformis. A neighbor-joining analysis with Jukes-Cantor correction and bootstrap support was performed on the gene sequences. Bootstrap values are given at nodes. Bold sequences are the ones considered as framework in the study. Values in parentheses are accession numbers (http://rdp.cme.msu.edu/). (1.63 MB TIF) [file pone.0004438.s010.tif]

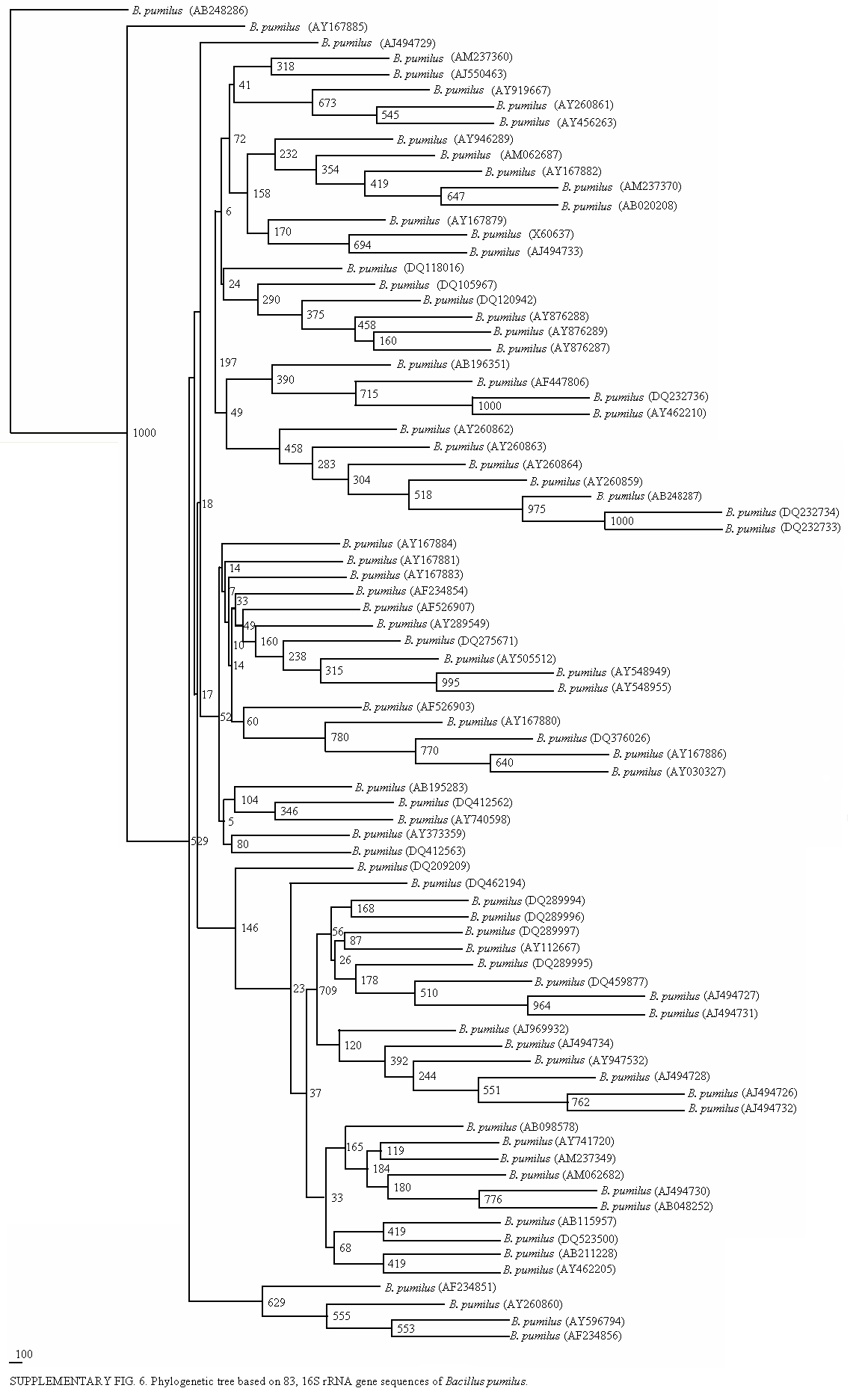

Supplement: Figure S6 — Phylogenetic tree based on 83, 16S rRNA gene sequences of Bacillus pumilus. A neighbor-joining analysis with Jukes-Cantor correction and bootstrap support was performed on the gene sequences. Bootstrap values are given at nodes. Bold sequences are the ones considered as framework in the study. Values in parentheses are accession numbers (http://rdp.cme.msu.edu/). (7.14 MB TIF) [file pone.0004438.s011.tif]

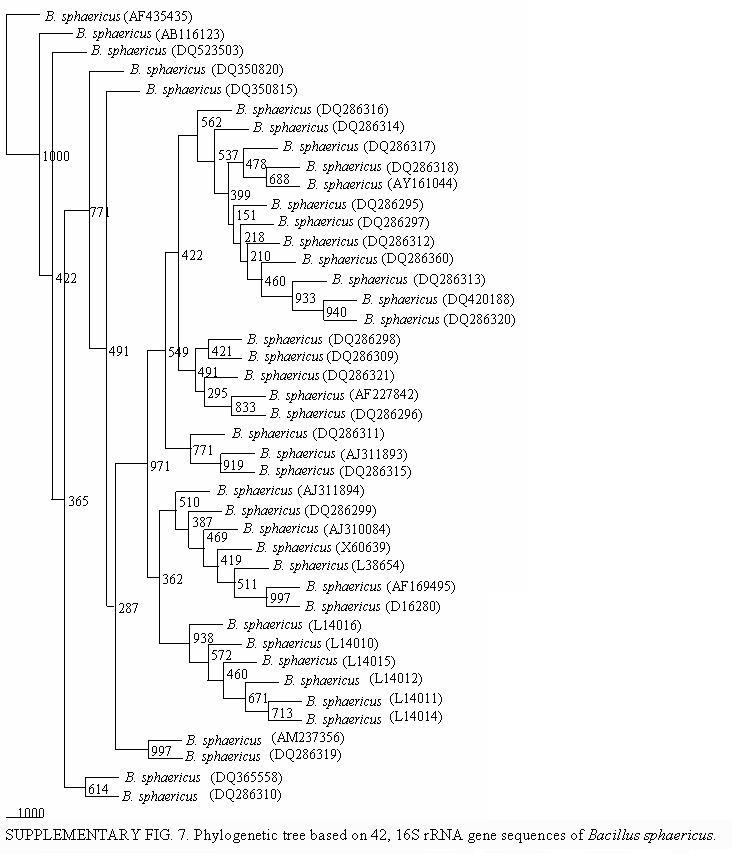

Supplement: Figure S7 — Phylogenetic tree based on 42, 16S rRNA gene sequences of Bacillus sphaericus. A neighbor-joining analysis with Jukes-Cantor correction and bootstrap support was performed on the gene sequences. Bootstrap values are given at nodes. Bold sequences are the ones considered as framework in the study. Values in parentheses are accession numbers (http://rdp.cme.msu.edu/). (0.75 MB TIF) [file pone.0004438.s012.tif]

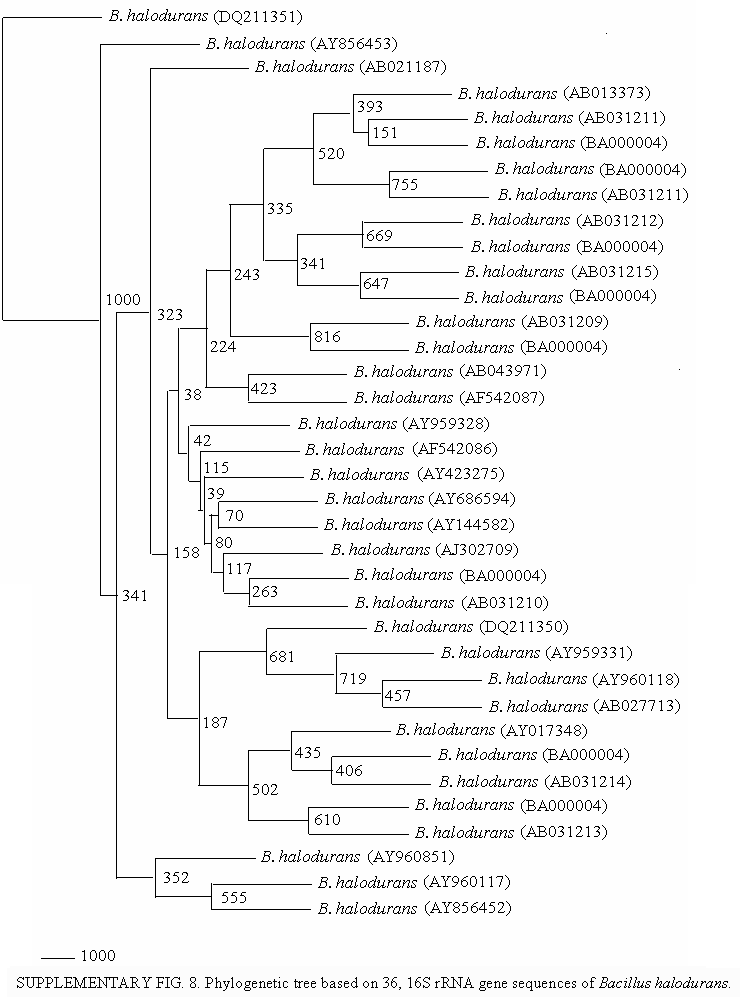

Supplement: Figure S8 — Phylogenetic tree based on 36, 16S rRNA gene sequences of Bacillus halodurans. A neighbor-joining analysis with Jukes-Cantor correction and bootstrap support was performed on the gene sequences. Bootstrap values are given at nodes. Bold sequences are the ones considered as framework in the study. Values in parentheses are accession numbers (http://rdp.cme.msu.edu/). (0.87 MB TIF) [file pone.0004438.s013.tif]

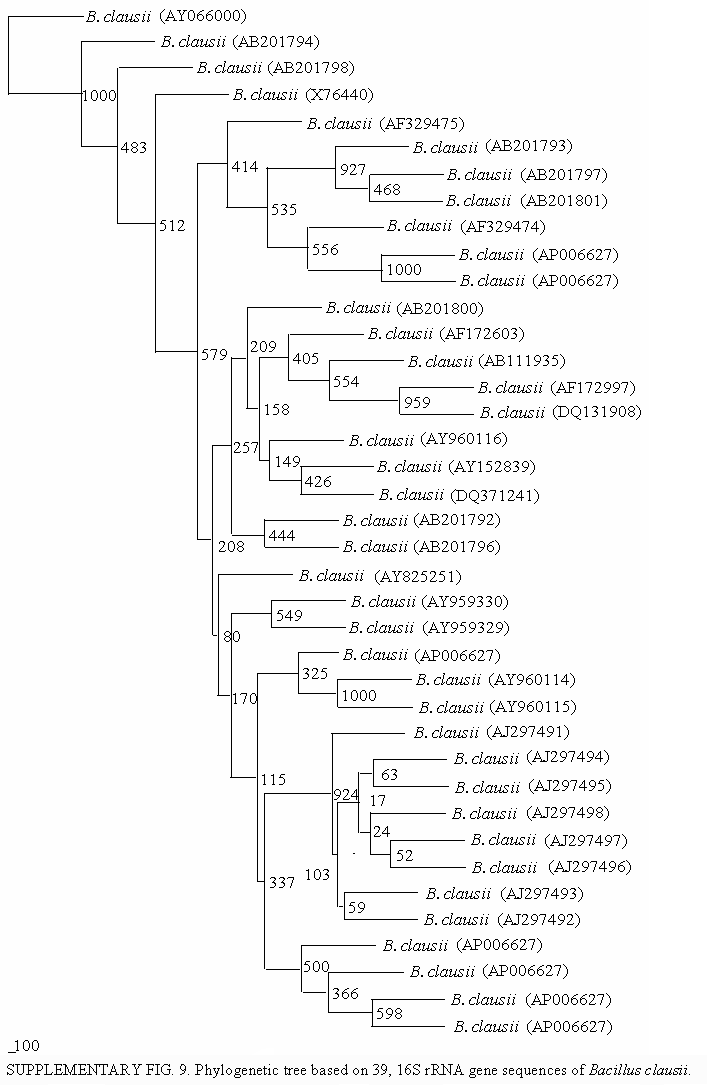

Supplement: Figure S9 — Phylogenetic tree based on 39, 16S rRNA gene sequences of Bacillus clausii. A neighbor-joining analysis with Jukes-Cantor correction and bootstrap support was performed on the gene sequences. Bootstrap values are given at nodes. Bold sequences are the ones considered as framework in the study. Values in parentheses are accession numbers (http://rdp.cme.msu.edu/). (0.89 MB TIF) [file pone.0004438.s014.tif]

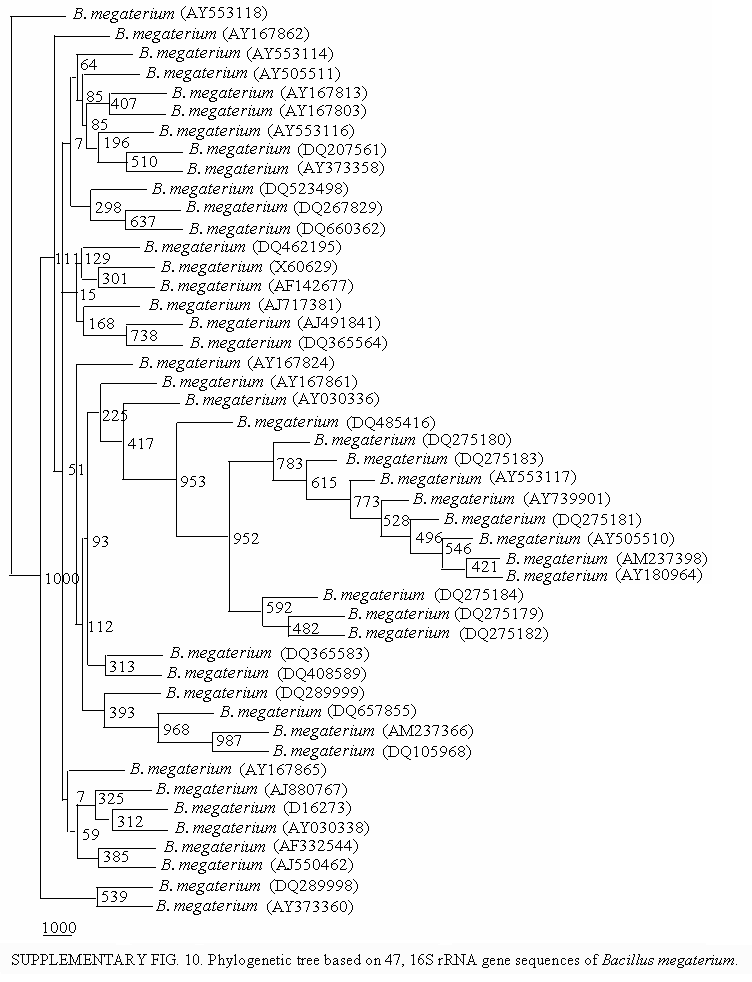

Supplement: Figure S10 — Phylogenetic tree based on 47, 16S rRNA gene sequences of Bacillus megaterium. A neighbor-joining analysis with Jukes-Cantor correction and bootstrap support was performed on the gene sequences. Bootstrap values are given at nodes. Bold sequences are the ones considered as framework in the study. Values in parentheses are accession numbers (http://rdp.cme.msu.edu/). (0.88 MB TIF) [file pone.0004438.s015.tif]

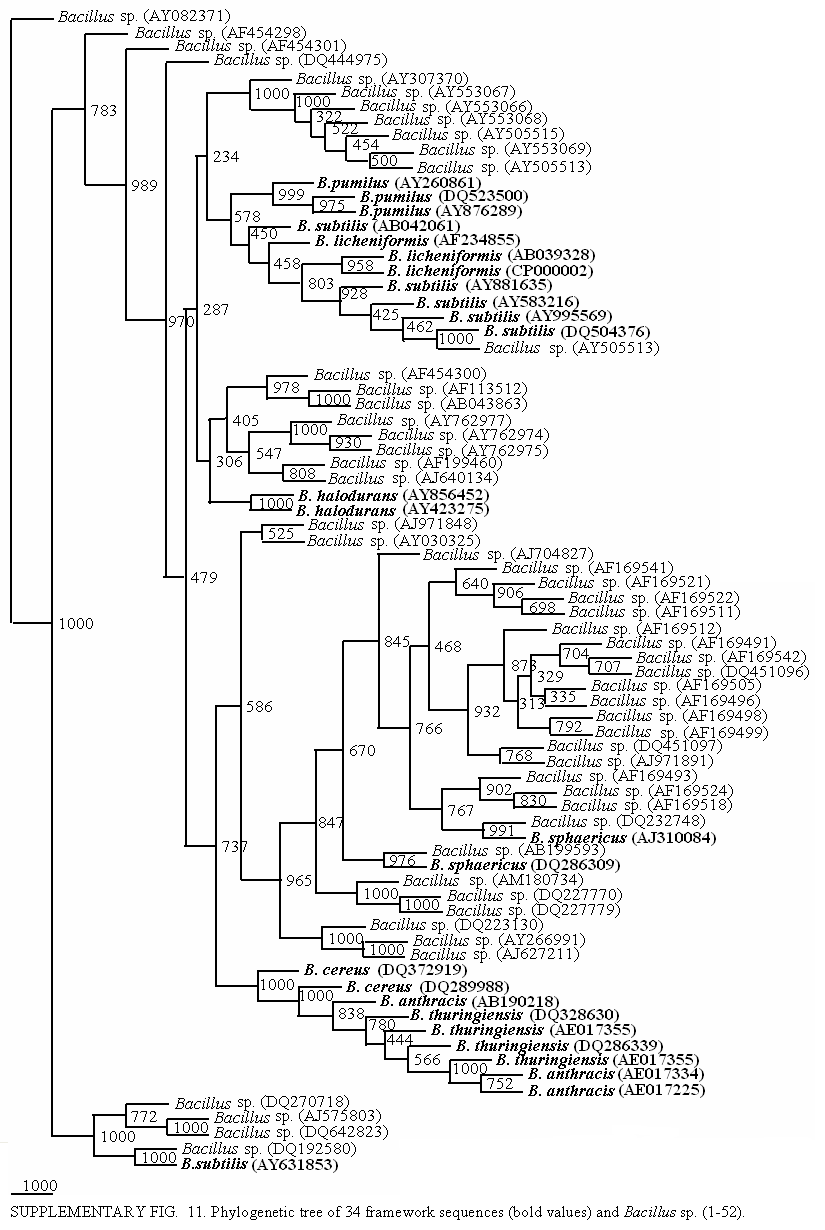

Supplement: Figure S11 — Phylogenetic tree of 34 framework sequences (bold values) and Bacillus sp. at the rate of 52 sequences (1–1136 {including 1025 Bacillus sp. and rest other species to add authenticity to the results}) which could be designated as 10 known Bacillus spp. used in the study. A neighbor-joining analysis with Jukes-Cantor correction and bootstrap support was performed on the gene sequences. Bootstrap values are given at nodes. Values in parentheses are accession numbers (http://rdp.cme.msu.edu/). (3.62 MB TIF) [file pone.0004438.s016.tif]

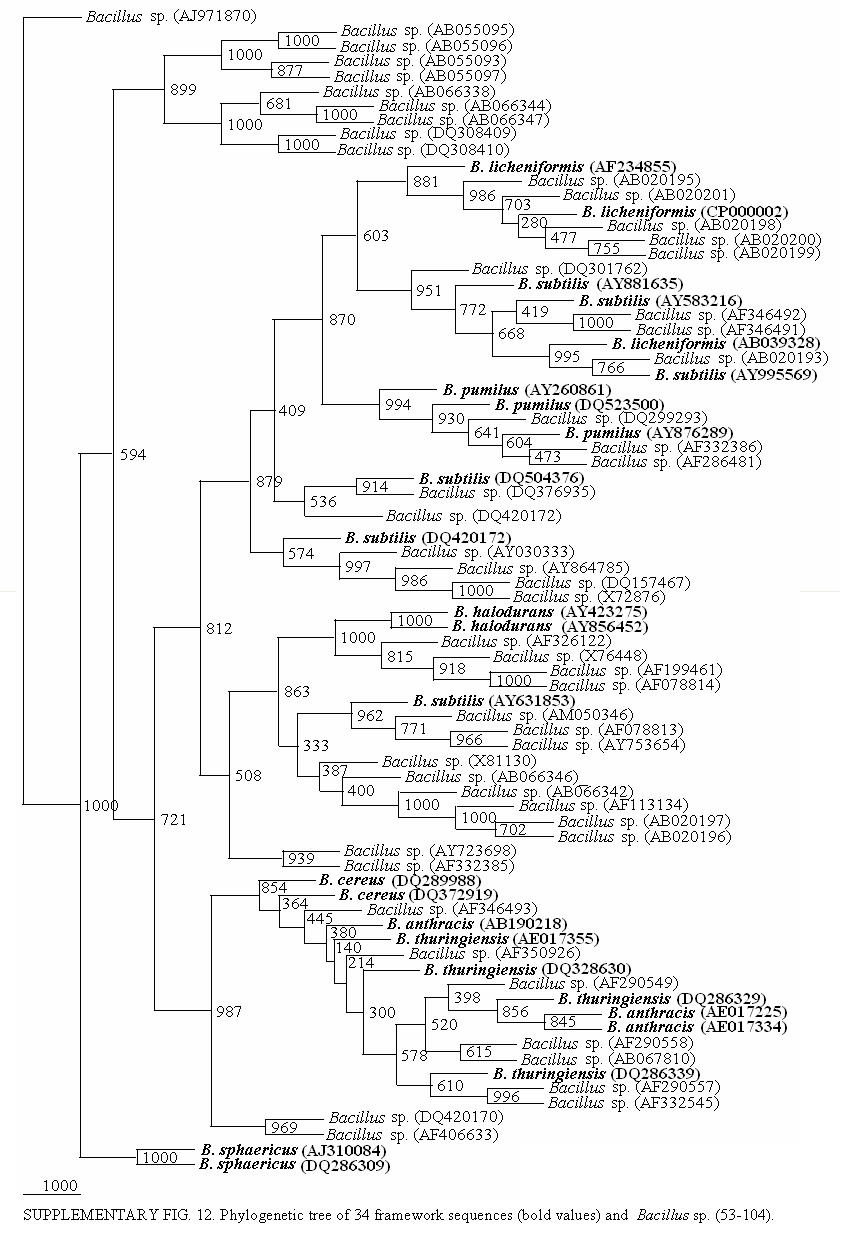

Supplement: Figure S12 — Phylogenetic tree of 34 framework sequences (bold values) and Bacillus sp. at the rate of 52 sequences (1–1136 {including 1025 Bacillus sp. and rest other species to add authenticity to the results}) which could be designated as 10 known Bacillus spp. used in the study. A neighbor-joining analysis with Jukes-Cantor correction and bootstrap support was performed on the gene sequences. Bootstrap values are given at nodes. Values in parentheses are accession numbers (http://rdp.cme.msu.edu/). (0.74 MB TIF) [file pone.0004438.s017.tif]

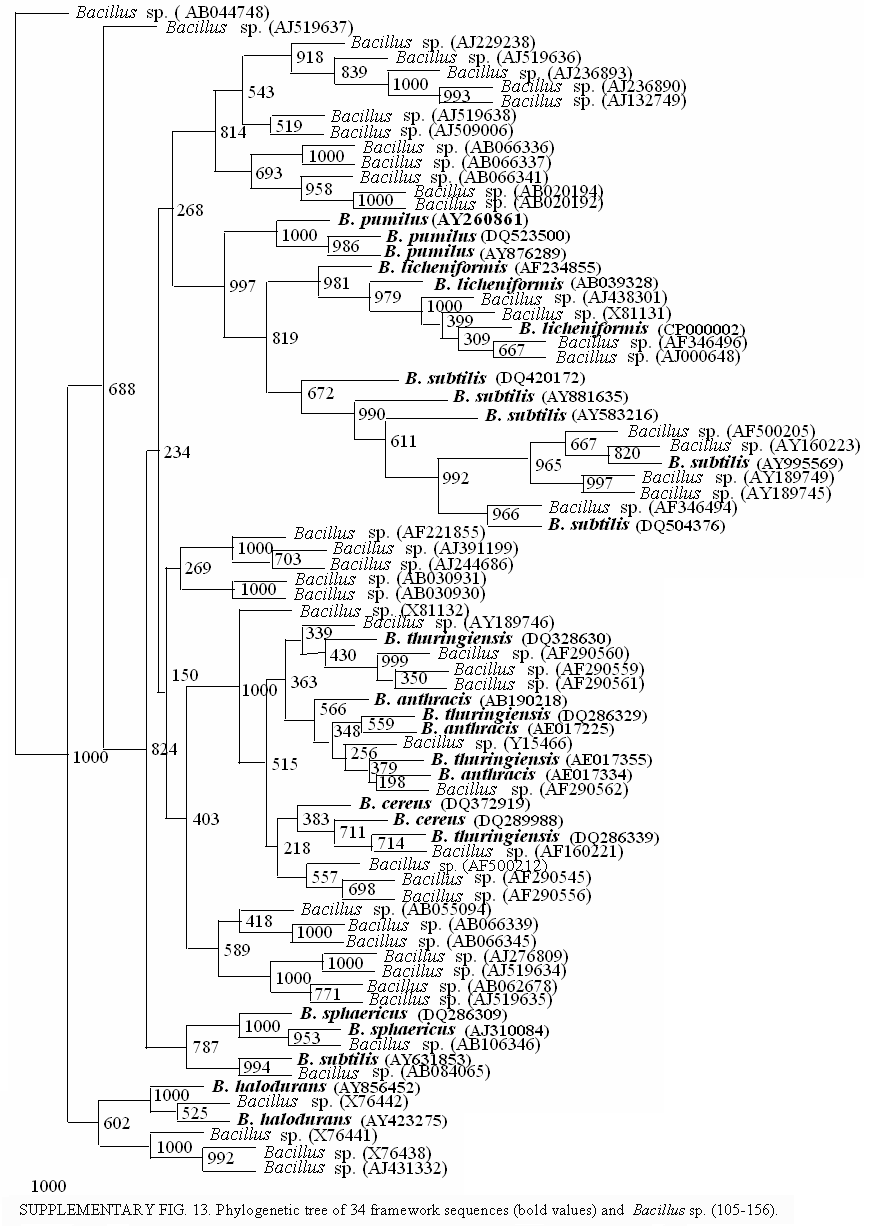

Supplement: Figure S13 — Phylogenetic tree of 34 framework sequences (bold values) and Bacillus sp. at the rate of 52 sequences (1–1136 {including 1025 Bacillus sp. and rest other species to add authenticity to the results}) which could be designated as 10 known Bacillus spp. used in the study. A neighbor-joining analysis with Jukes-Cantor correction and bootstrap support was performed on the gene sequences. Bootstrap values are given at nodes. Values in parentheses are accession numbers (http://rdp.cme.msu.edu/). (0.37 MB TIF) [file pone.0004438.s018.tif]

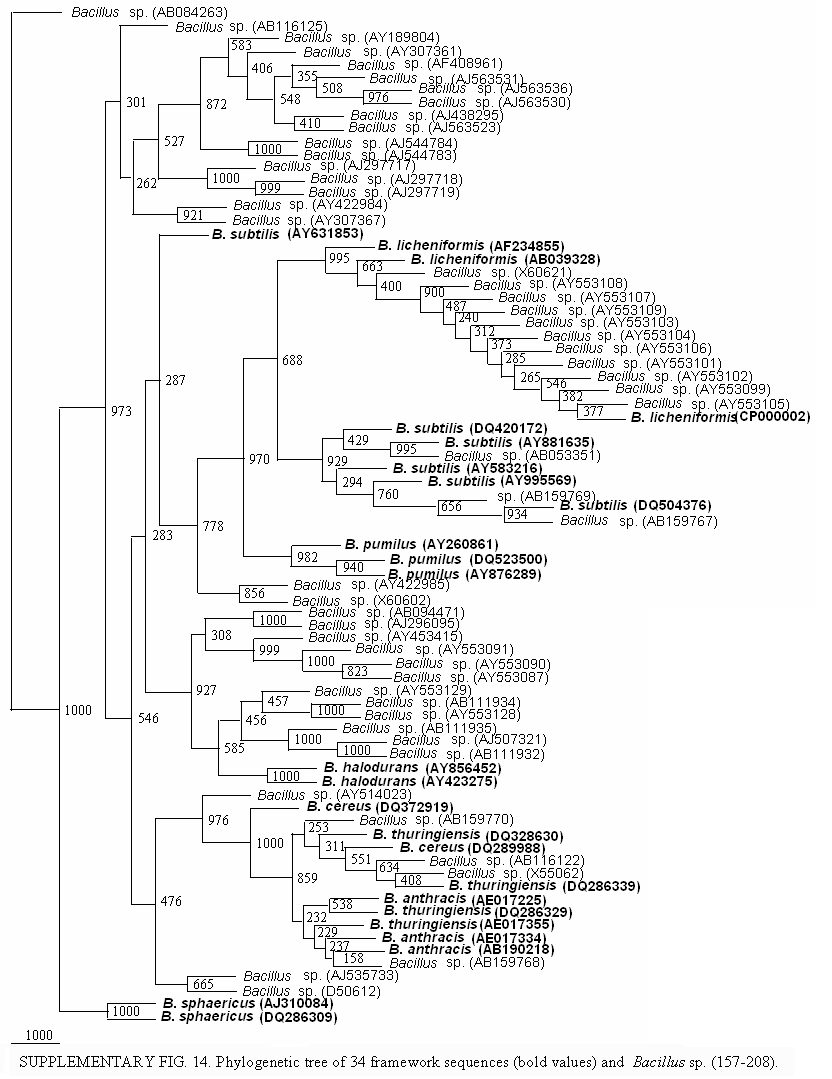

Supplement: Figure S14 — Phylogenetic tree of 34 framework sequences (bold values) and Bacillus sp. at the rate of 52 sequences (1–1136 {including 1025 Bacillus sp. and rest other species to add authenticity to the results}) which could be designated as 10 known Bacillus spp. used in the study. A neighbor-joining analysis with Jukes-Cantor correction and bootstrap support was performed on the gene sequences. Bootstrap values are given at nodes. Values in parentheses are accession numbers (http://rdp.cme.msu.edu/). (0.26 MB TIF) [file pone.0004438.s019.tif]

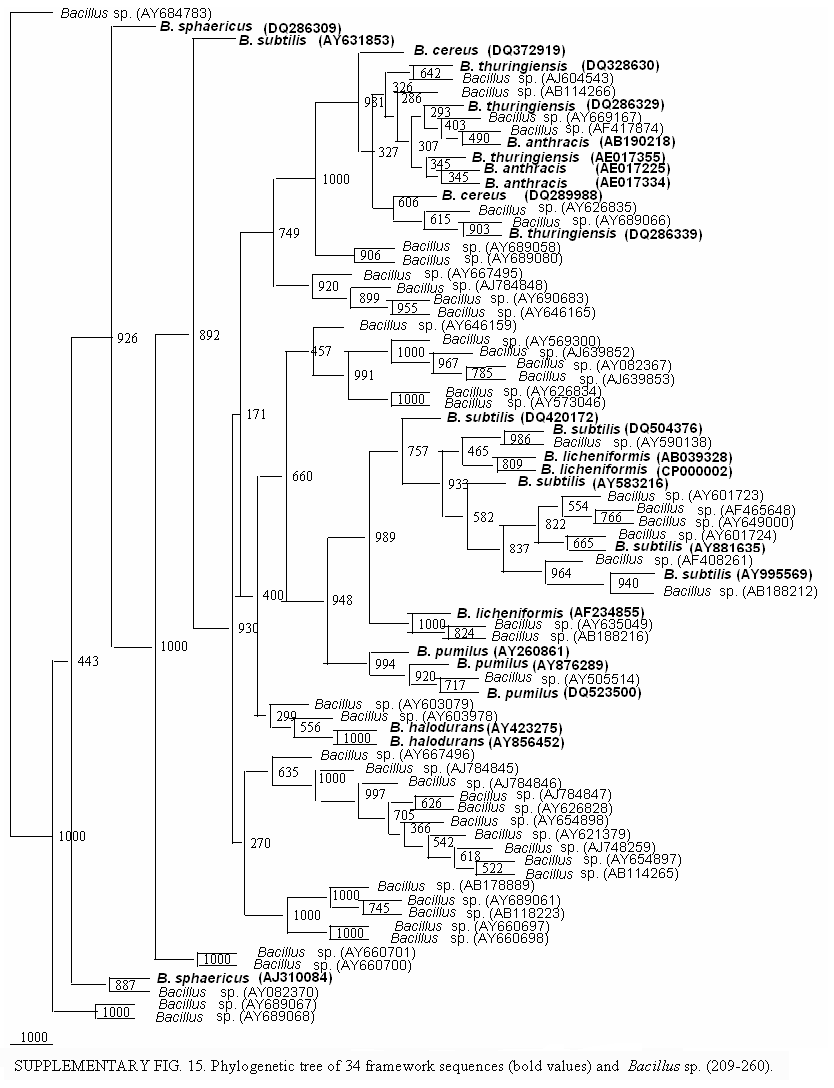

Supplement: Figure S15 — Phylogenetic tree of 34 framework sequences (bold values) and Bacillus sp. at the rate of 52 sequences (1–1136 {including 1025 Bacillus sp. and rest other species to add authenticity to the results}) which could be designated as 10 known Bacillus spp. used in the study. A neighbor-joining analysis with Jukes-Cantor correction and bootstrap support was performed on the gene sequences. Bootstrap values are given at nodes. Values in parentheses are accession numbers (http://rdp.cme.msu.edu/). (0.28 MB TIF) [file pone.0004438.s020.tif]

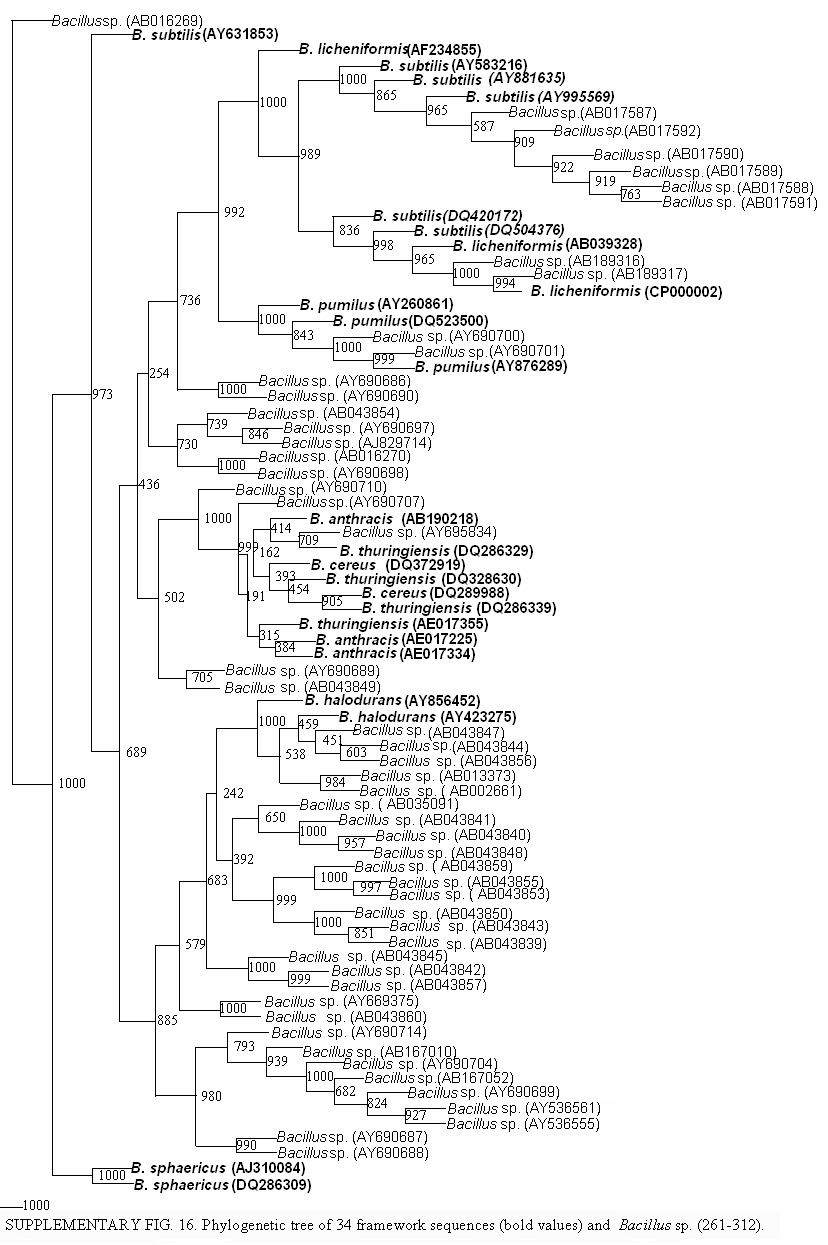

Supplement: Figure S16 — Phylogenetic tree of 34 framework sequences (bold values) and Bacillus sp. at the rate of 52 sequences (1–1136 {including 1025 Bacillus sp. and rest other species to add authenticity to the results}) which could be designated as 10 known Bacillus spp. used in the study. A neighbor-joining analysis with Jukes-Cantor correction and bootstrap support was performed on the gene sequences. Bootstrap values are given at nodes. Values in parentheses are accession numbers (http://rdp.cme.msu.edu/). (0.29 MB TIF) [file pone.0004438.s021.tif]

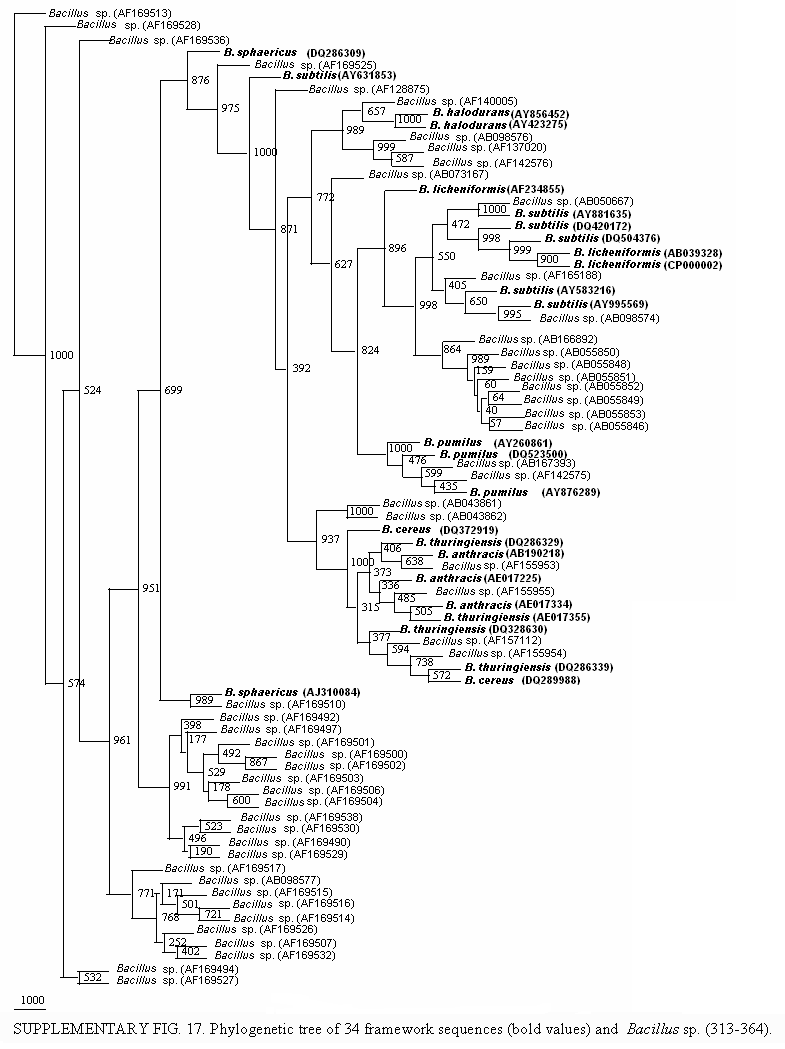

Supplement: Figure S17 — Phylogenetic tree of 34 framework sequences (bold values) and Bacillus sp. at the rate of 52 sequences (1–1136 {including 1025 Bacillus sp. and rest other species to add authenticity to the results}) which could be designated as 10 known Bacillus spp. used in the study. A neighbor-joining analysis with Jukes-Cantor correction and bootstrap support was performed on the gene sequences. Bootstrap values are given at nodes. Values in parentheses are accession numbers (http://rdp.cme.msu.edu/). (0.22 MB TIF) [file pone.0004438.s022.tif]

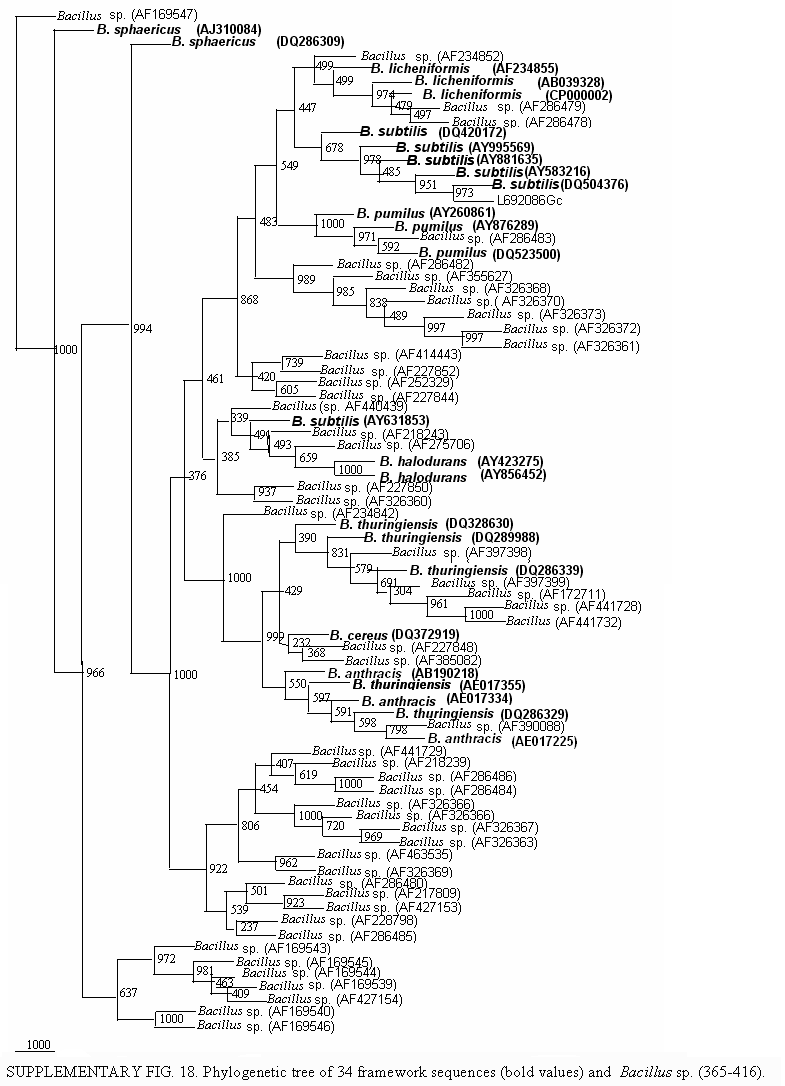

Supplement: Figure S18 — Phylogenetic tree of 34 framework sequences (bold values) and Bacillus sp. at the rate of 52 sequences (1–1136 {including 1025 Bacillus sp. and rest other species to add authenticity to the results}) which could be designated as 10 known Bacillus spp. used in the study. A neighbor-joining analysis with Jukes-Cantor correction and bootstrap support was performed on the gene sequences. Bootstrap values are given at nodes. Values in parentheses are accession numbers (http://rdp.cme.msu.edu/). (0.25 MB TIF) [file pone.0004438.s023.tif]

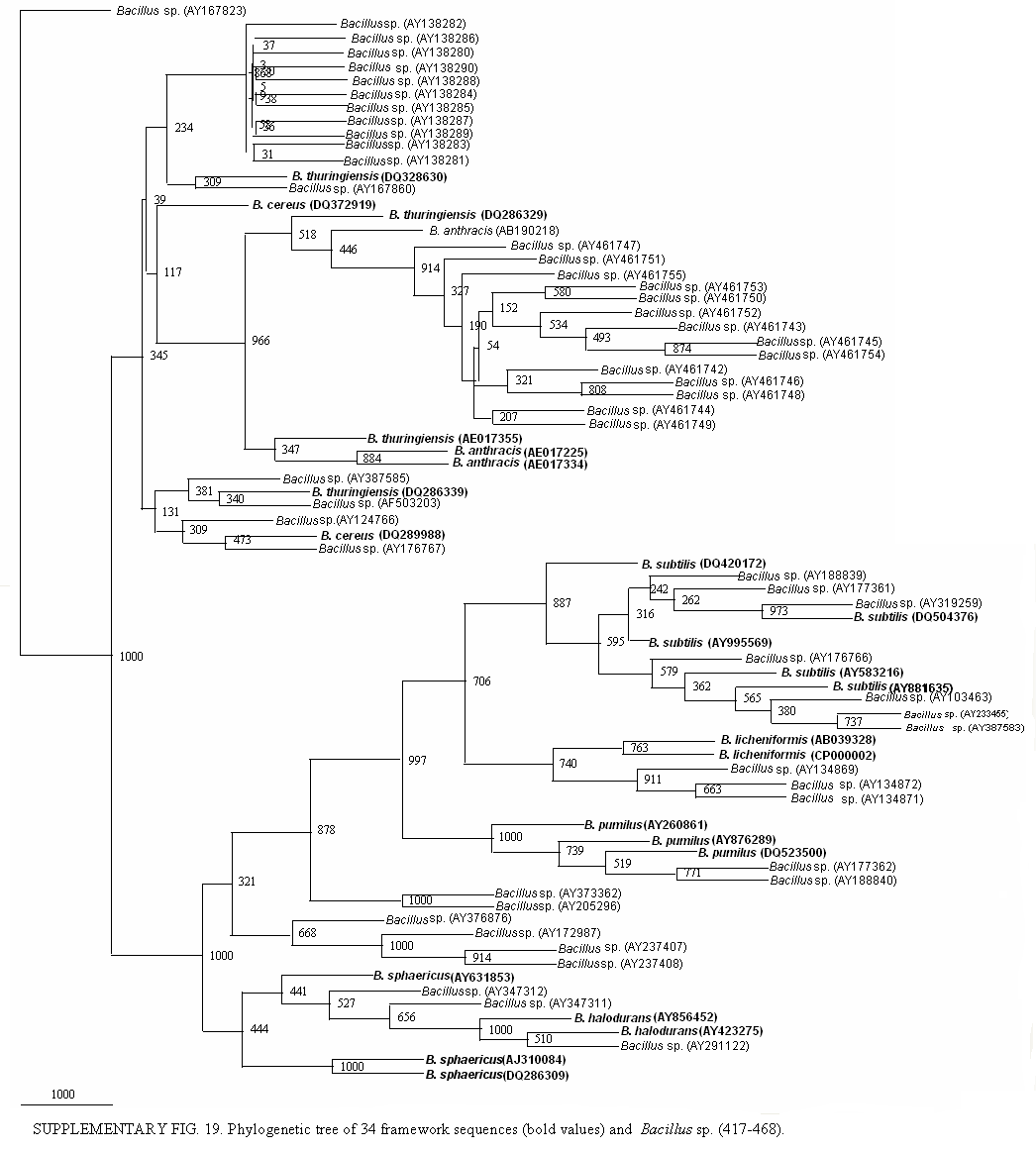

Supplement: Figure S19 — Phylogenetic tree of 34 framework sequences (bold values) and Bacillus sp. at the rate of 52 sequences (1–1136 {including 1025 Bacillus sp. and rest other species to add authenticity to the results}) which could be designated as 10 known Bacillus spp. used in the study. A neighbor-joining analysis with Jukes-Cantor correction and bootstrap support was performed on the gene sequences. Bootstrap values are given at nodes. Values in parentheses are accession numbers (http://rdp.cme.msu.edu/). (0.67 MB TIF) [file pone.0004438.s024.tif]

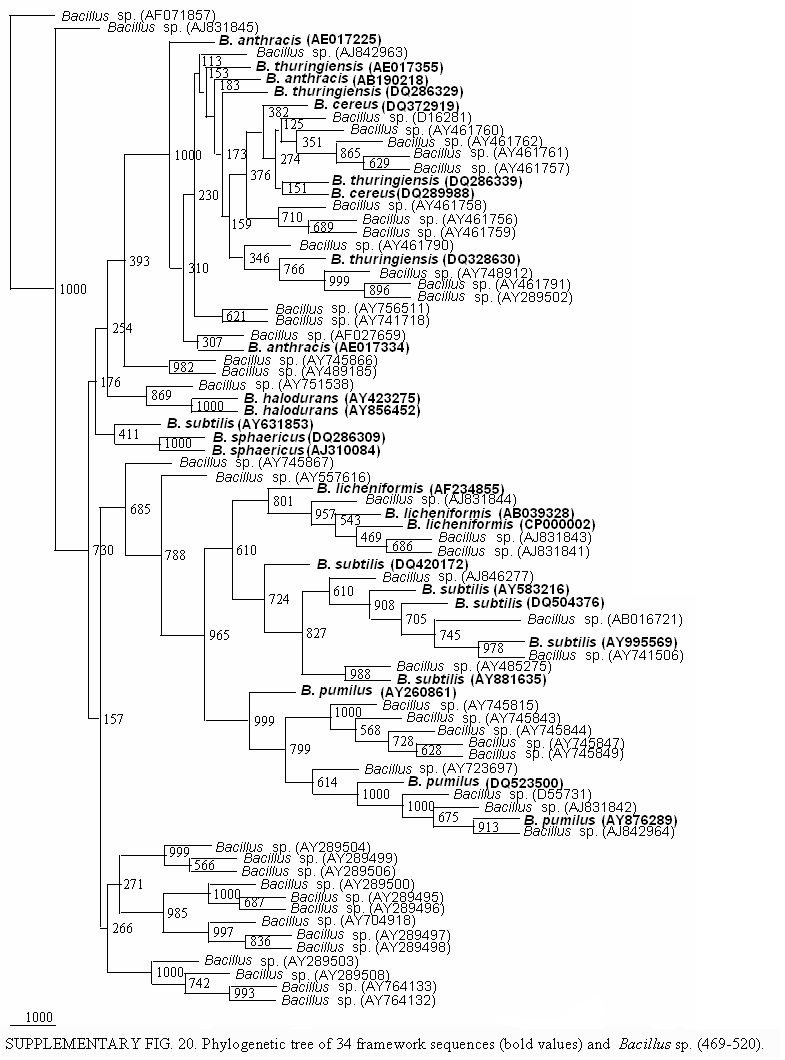

Supplement: Figure S20 — Phylogenetic tree of 34 framework sequences (bold values) and Bacillus sp. at the rate of 52 sequences (1–1136 {including 1025 Bacillus sp. and rest other species to add authenticity to the results}) which could be designated as 10 known Bacillus spp. used in the study. A neighbor-joining analysis with Jukes-Cantor correction and bootstrap support was performed on the gene sequences. Bootstrap values are given at nodes. Values in parentheses are accession numbers (http://rdp.cme.msu.edu/). (0.26 MB TIF) [file pone.0004438.s025.tif]

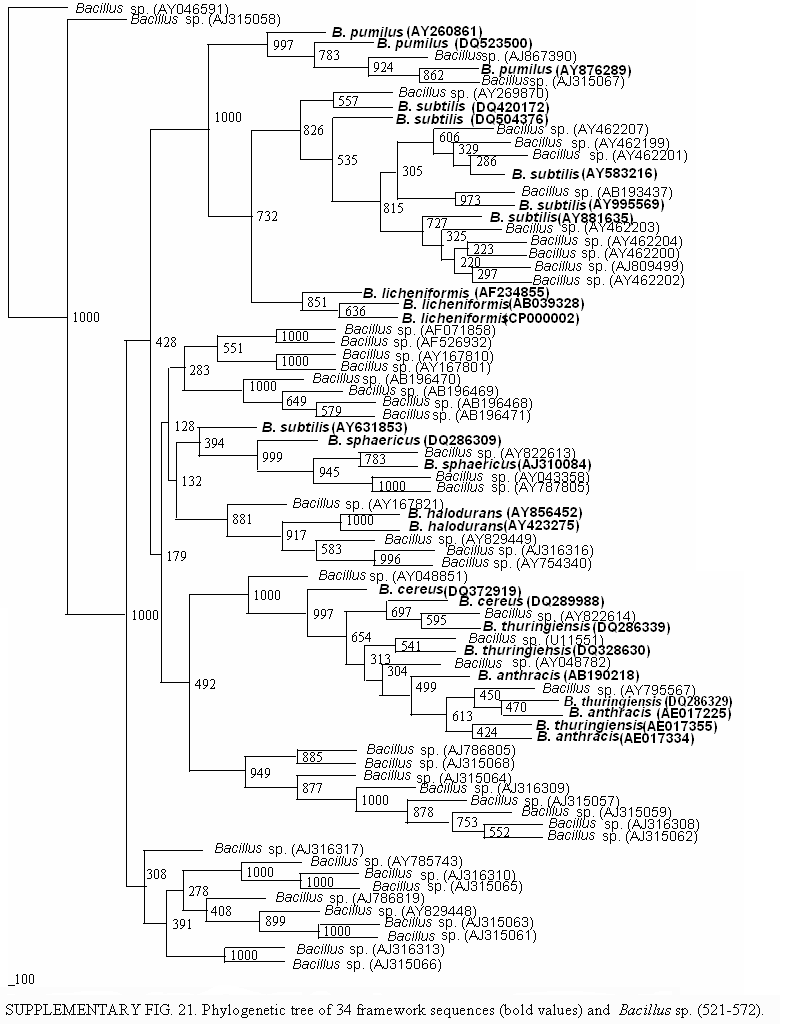

Supplement: Figure S21 — Phylogenetic tree of 34 framework sequences (bold values) and Bacillus sp. at the rate of 52 sequences (1–1136 {including 1025 Bacillus sp. and rest other species to add authenticity to the results}) which could be designated as 10 known Bacillus spp. used in the study. A neighbor-joining analysis with Jukes-Cantor correction and bootstrap support was performed on the gene sequences. Bootstrap values are given at nodes. Values in parentheses are accession numbers (http://rdp.cme.msu.edu/). (0.25 MB TIF) [file pone.0004438.s026.tif]

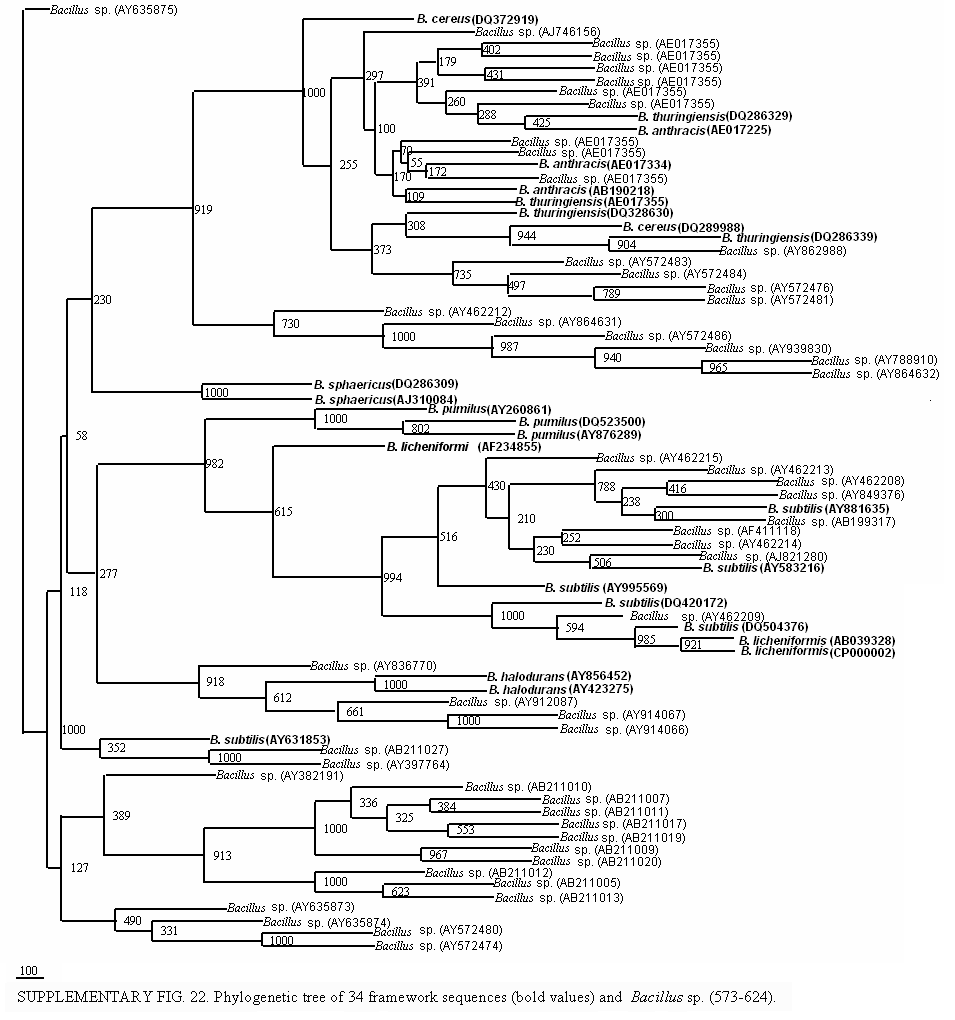

Supplement: Figure S22 — Phylogenetic tree of 34 framework sequences (bold values) and Bacillus sp. at the rate of 52 sequences (1–1136 {including 1025 Bacillus sp. and rest other species to add authenticity to the results}) which could be designated as 10 known Bacillus spp. used in the study. A neighbor-joining analysis with Jukes-Cantor correction and bootstrap support was performed on the gene sequences. Bootstrap values are given at nodes. Values in parentheses are accession numbers (http://rdp.cme.msu.edu/). (0.24 MB TIF) [file pone.0004438.s027.tif]

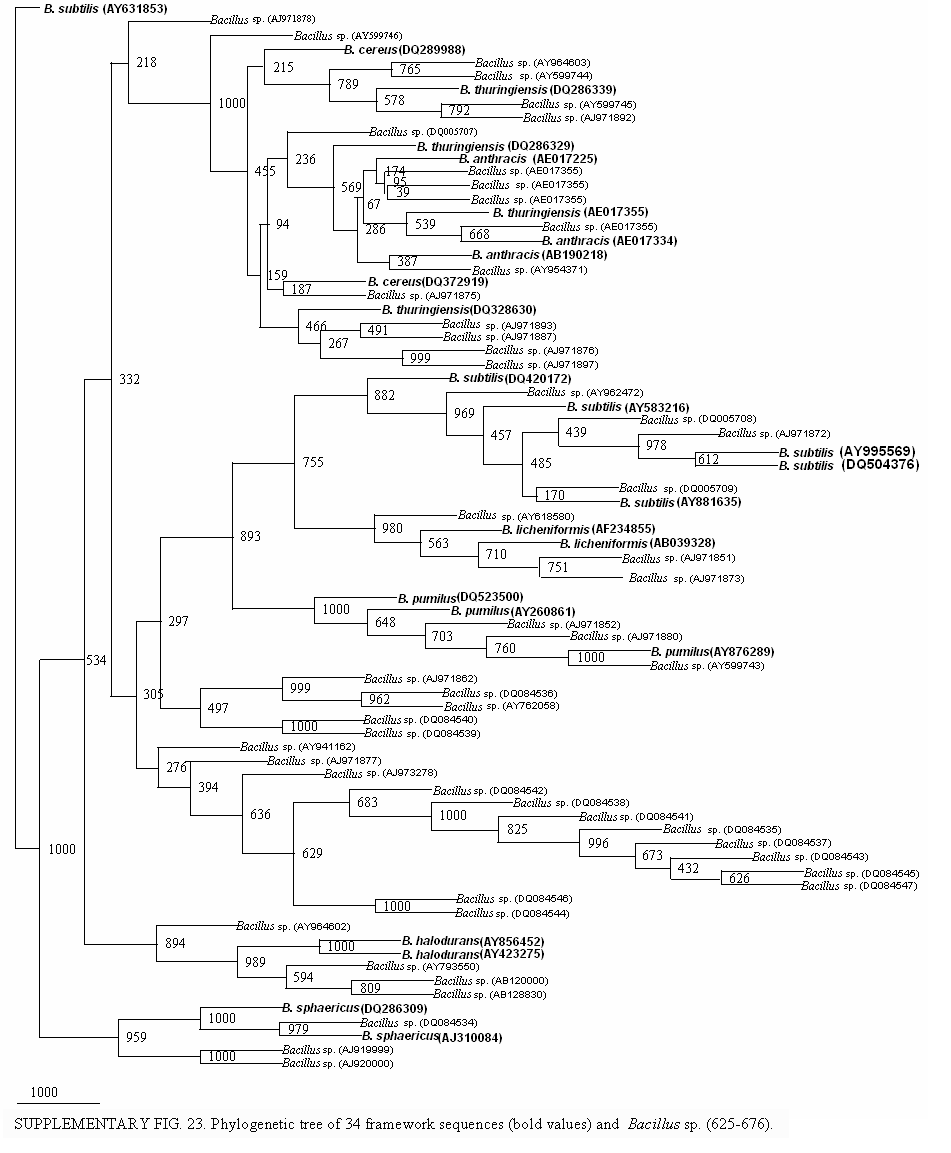

Supplement: Figure S23 — Phylogenetic tree of 34 framework sequences (bold values) and Bacillus sp. at the rate of 52 sequences (1–1136 {including 1025 Bacillus sp. and rest other species to add authenticity to the results}) which could be designated as 10 known Bacillus spp. used in the study. A neighbor-joining analysis with Jukes-Cantor correction and bootstrap support was performed on the gene sequences. Bootstrap values are given at nodes. Values in parentheses are accession numbers (http://rdp.cme.msu.edu/). (0.24 MB TIF) [file pone.0004438.s028.tif]

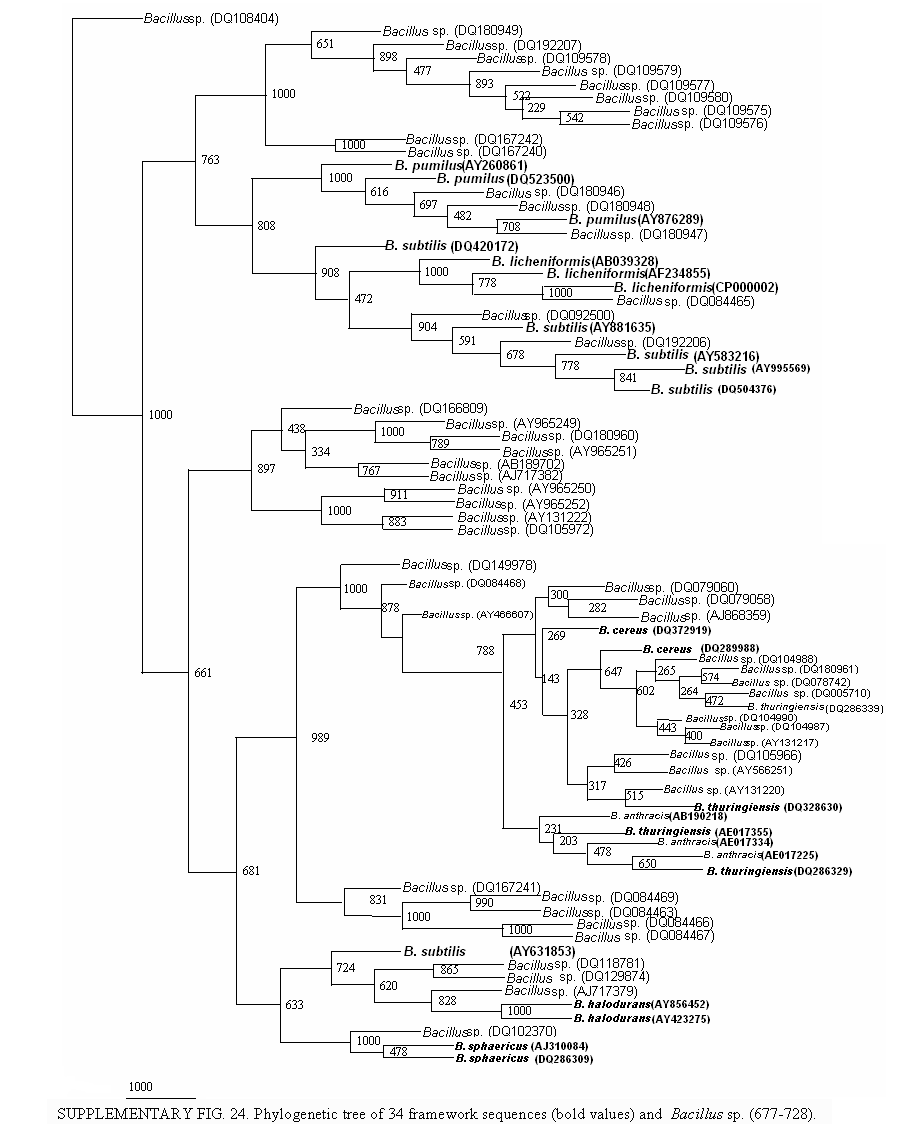

Supplement: Figure S24 — Phylogenetic tree of 34 framework sequences (bold values) and Bacillus sp. at the rate of 52 sequences (1–1136 {including 1025 Bacillus sp. and rest other species to add authenticity to the results}) which could be designated as 10 known Bacillus spp. used in the study. A neighbor-joining analysis with Jukes-Cantor correction and bootstrap support was performed on the gene sequences. Bootstrap values are given at nodes. Values in parentheses are accession numbers (http://rdp.cme.msu.edu/). (0.25 MB TIF) [file pone.0004438.s029.tif]

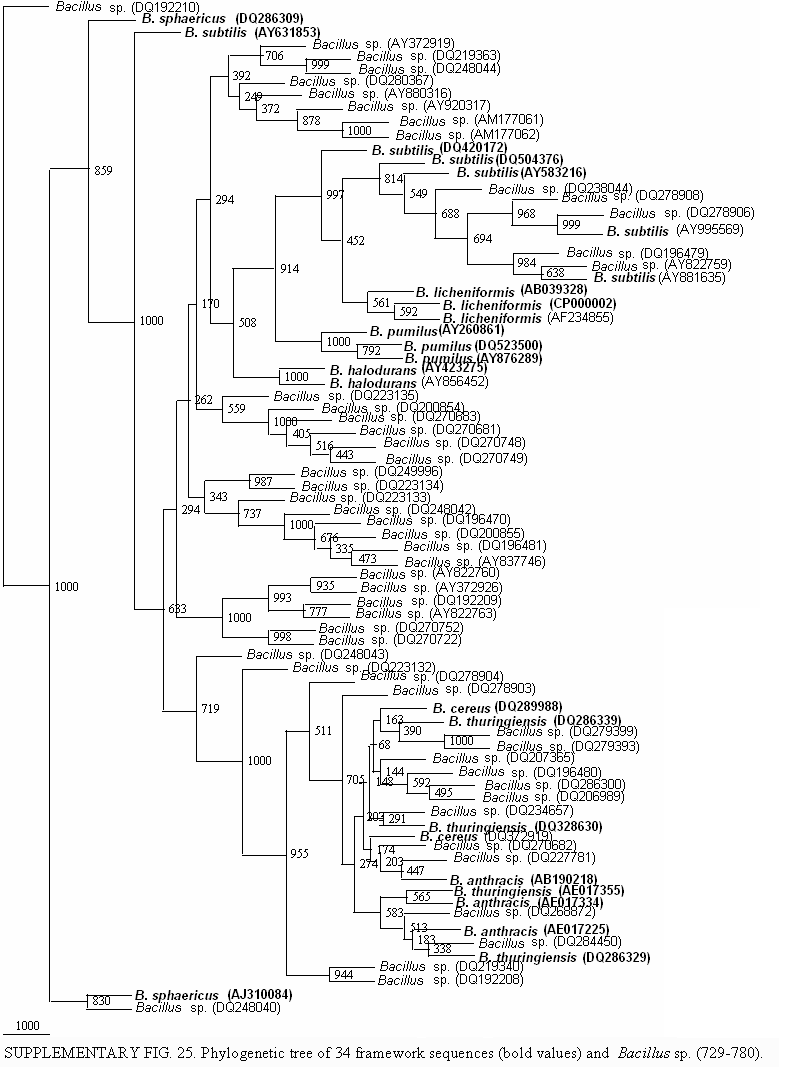

Supplement: Figure S25 — Phylogenetic tree of 34 framework sequences (bold values) and Bacillus sp. at the rate of 52 sequences (1–1136 {including 1025 Bacillus sp. and rest other species to add authenticity to the results}) which could be designated as 10 known Bacillus spp. used in the study. A neighbor-joining analysis with Jukes-Cantor correction and bootstrap support was performed on the gene sequences. Bootstrap values are given at nodes. Values in parentheses are accession numbers (http://rdp.cme.msu.edu/). (0.25 MB TIF) [file pone.0004438.s030.tif]

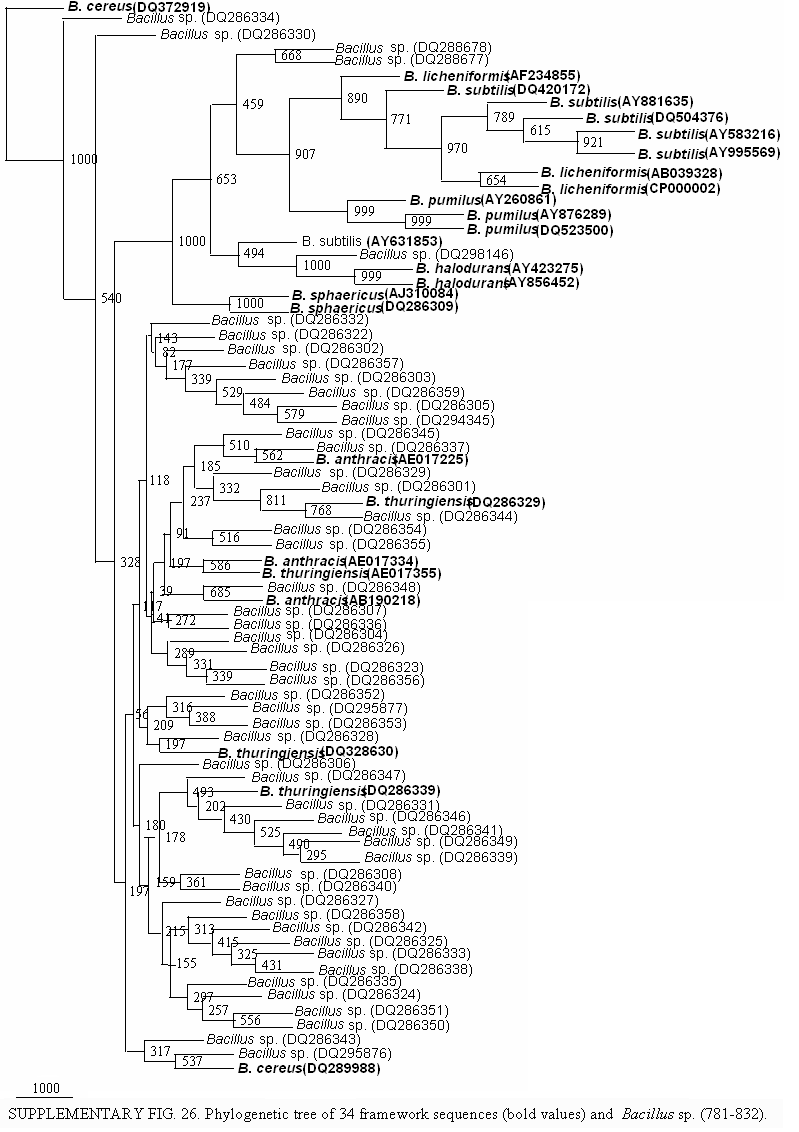

Supplement: Figure S26 — Phylogenetic tree of 34 framework sequences (bold values) and Bacillus sp. at the rate of 52 sequences (1–1136 {including 1025 Bacillus sp. and rest other species to add authenticity to the results}) which could be designated as 10 known Bacillus spp. used in the study. A neighbor-joining analysis with Jukes-Cantor correction and bootstrap support was performed on the gene sequences. Bootstrap values are given at nodes. Values in parentheses are accession numbers (http://rdp.cme.msu.edu/). (0.26 MB TIF) [file pone.0004438.s031.tif]

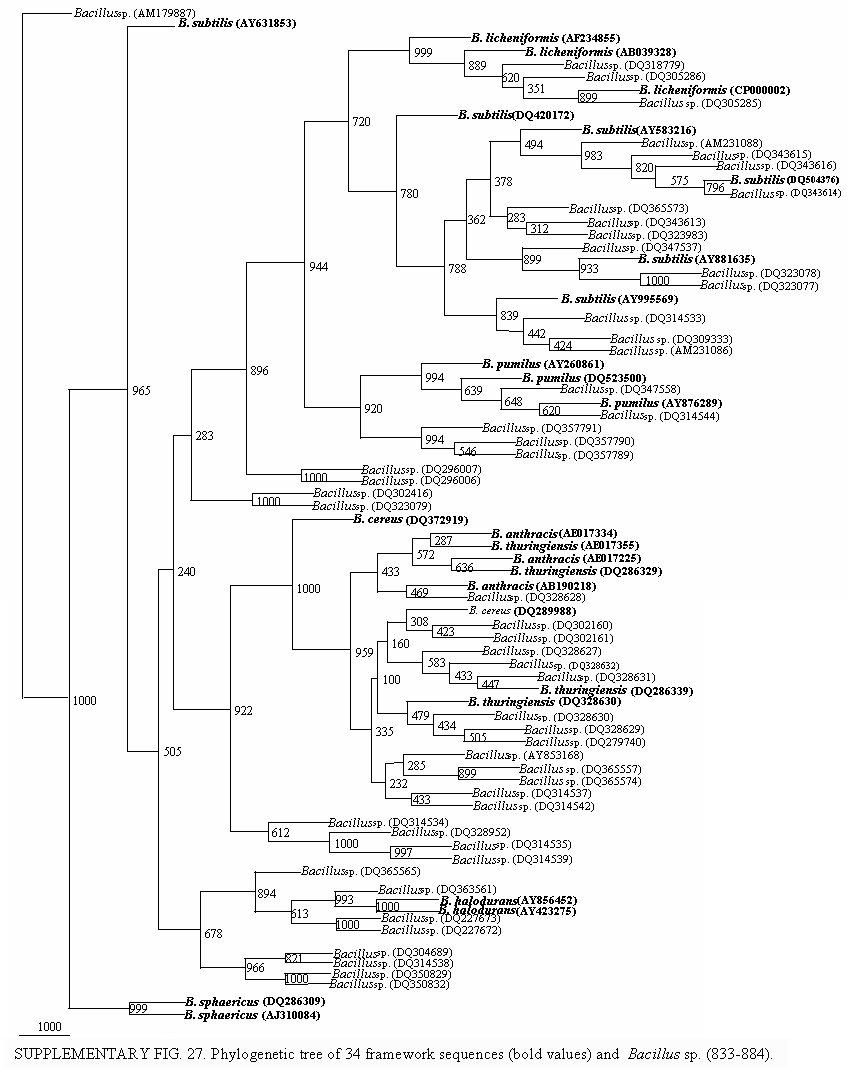

Supplement: Figure S27 — Phylogenetic tree of 34 framework sequences (bold values) and Bacillus sp. at the rate of 52 sequences (1–1136 {including 1025 Bacillus sp. and rest other species to add authenticity to the results}) which could be designated as 10 known Bacillus spp. used in the study. A neighbor-joining analysis with Jukes-Cantor correction and bootstrap support was performed on the gene sequences. Bootstrap values are given at nodes. Values in parentheses are accession numbers (http://rdp.cme.msu.edu/). (0.21 MB TIF) [file pone.0004438.s032.tif]

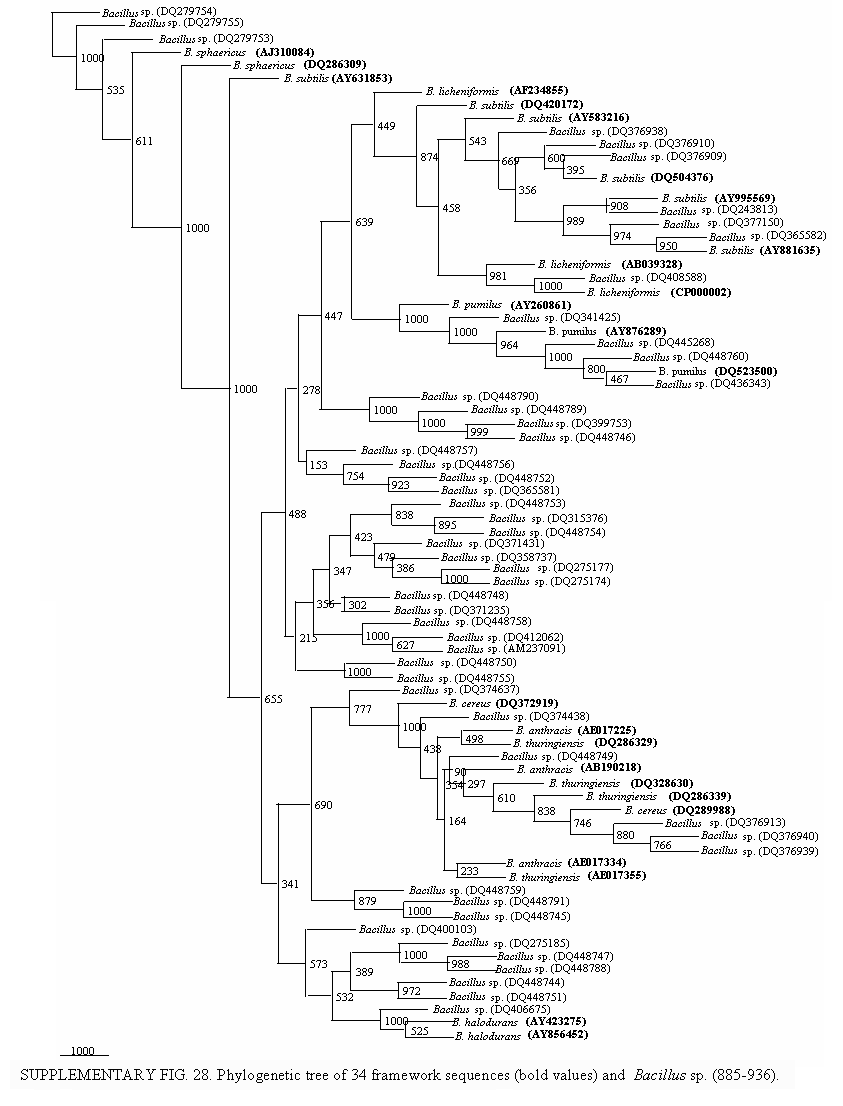

Supplement: Figure S28 — Phylogenetic tree of 34 framework sequences (bold values) and Bacillus sp. at the rate of 52 sequences (1–1136 {including 1025 Bacillus sp. and rest other species to add authenticity to the results}) which could be designated as 10 known Bacillus spp. used in the study. A neighbor-joining analysis with Jukes-Cantor correction and bootstrap support was performed on the gene sequences. Bootstrap values are given at nodes. Values in parentheses are accession numbers (http://rdp.cme.msu.edu/). (0.21 MB TIF) [file pone.0004438.s033.tif]

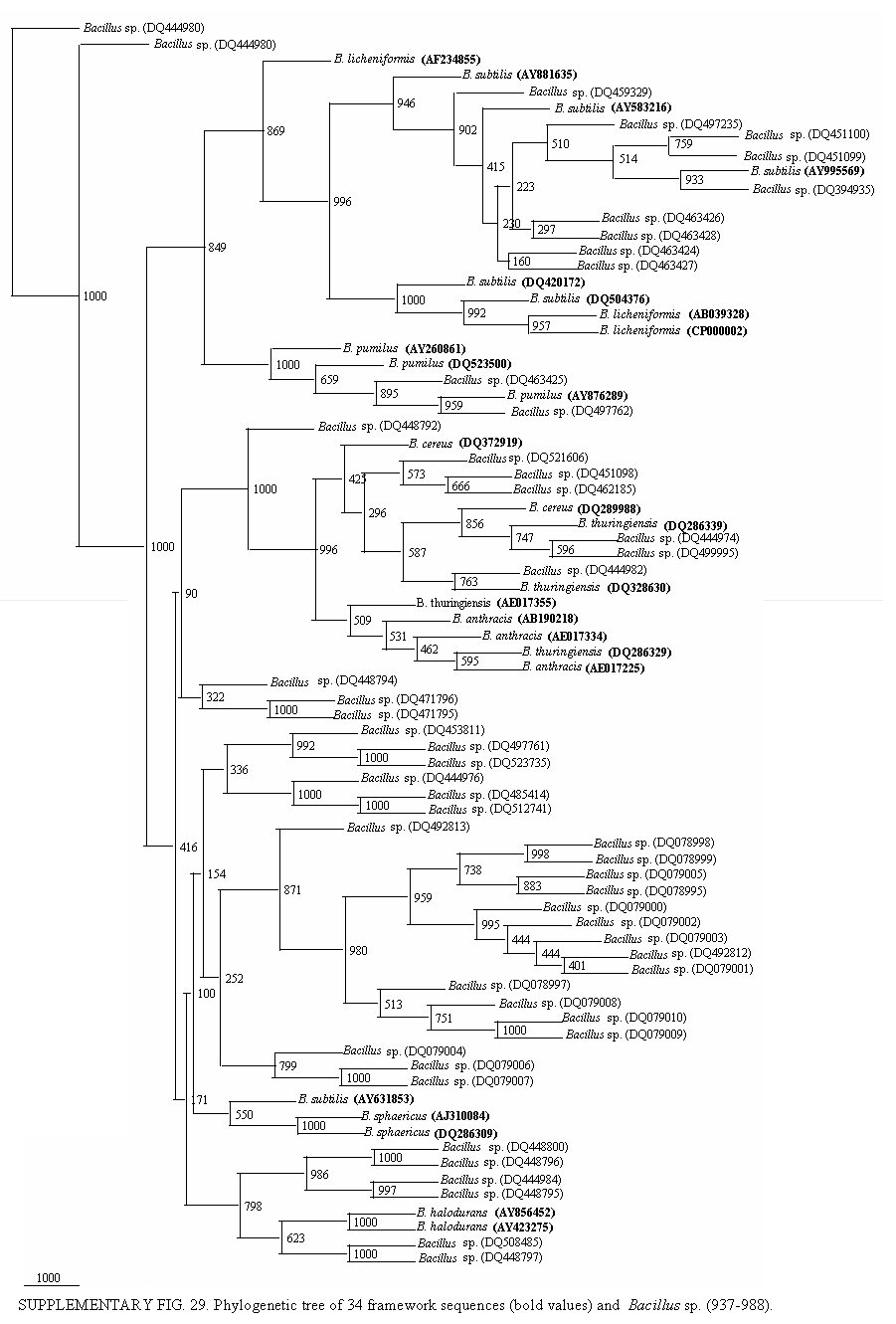

Supplement: Figure S29 — Phylogenetic tree of 34 framework sequences (bold values) and Bacillus sp. at the rate of 52 sequences (1–1136 {including 1025 Bacillus sp. and rest other species to add authenticity to the results}) which could be designated as 10 known Bacillus spp. used in the study. A neighbor-joining analysis with Jukes-Cantor correction and bootstrap support was performed on the gene sequences. Bootstrap values are given at nodes. Values in parentheses are accession numbers (http://rdp.cme.msu.edu/). (0.63 MB TIF) [file pone.0004438.s034.tif]

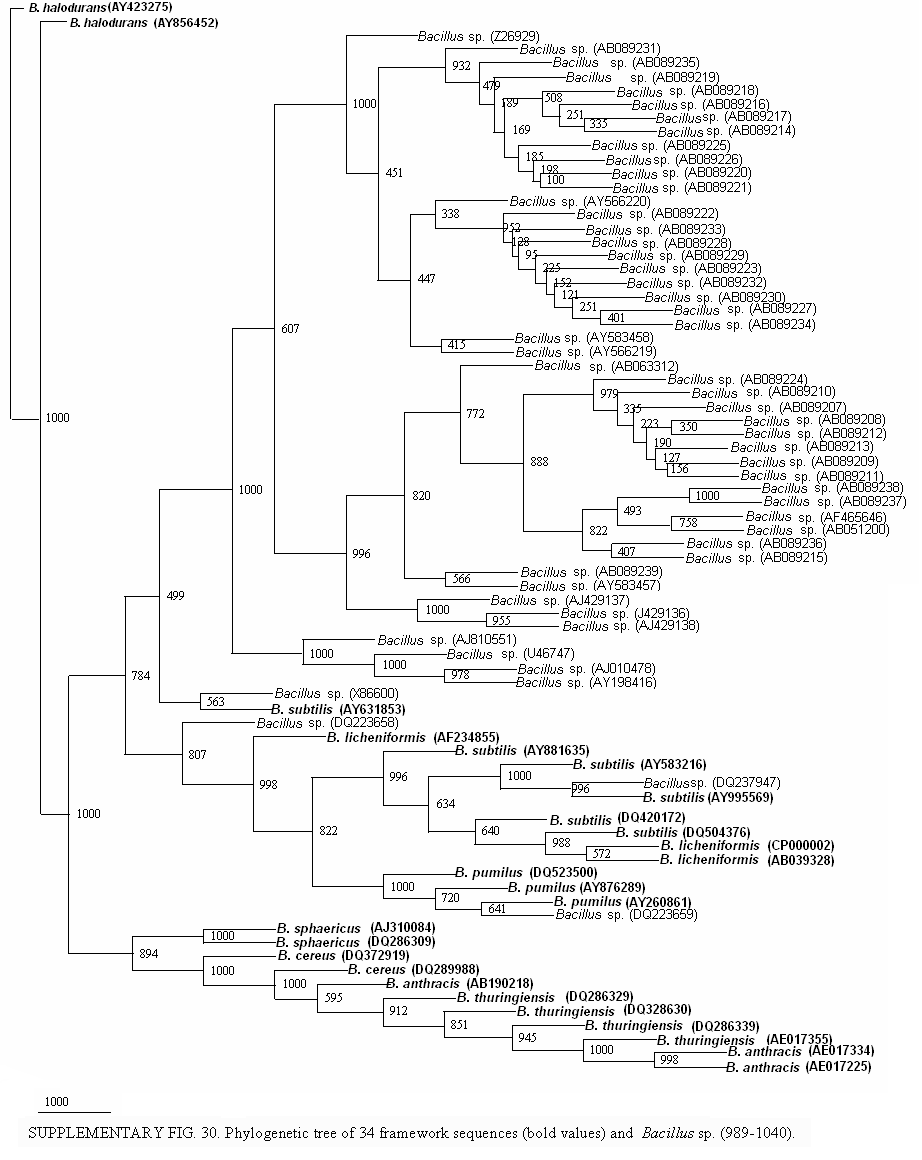

Supplement: Figure S30 — Phylogenetic tree of 34 framework sequences (bold values) and Bacillus sp. at the rate of 52 sequences (1–1136 {including 1025 Bacillus sp. and rest other species to add authenticity to the results}) which could be designated as 10 known Bacillus spp. used in the study. A neighbor-joining analysis with Jukes-Cantor correction and bootstrap support was performed on the gene sequences. Bootstrap values are given at nodes. Values in parentheses are accession numbers (http://rdp.cme.msu.edu/). (0.26 MB TIF) [file pone.0004438.s035.tif]

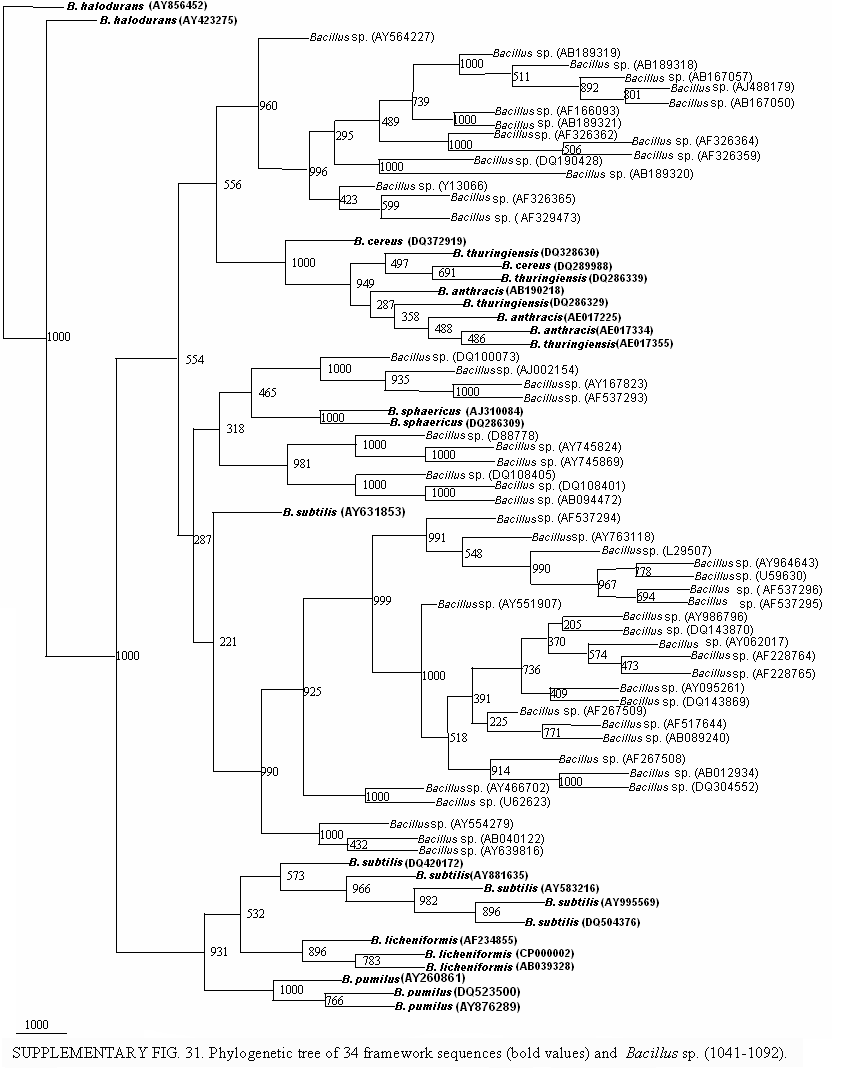

Supplement: Figure S31 — Phylogenetic tree of 34 framework sequences (bold values) and Bacillus sp. at the rate of 52 sequences (1–1136 {including 1025 Bacillus sp. and rest other species to add authenticity to the results}) which could be designated as 10 known Bacillus spp. used in the study. A neighbor-joining analysis with Jukes-Cantor correction and bootstrap support was performed on the gene sequences. Bootstrap values are given at nodes. Values in parentheses are accession numbers (http://rdp.cme.msu.edu/). (0.22 MB TIF) [file pone.0004438.s036.tif]

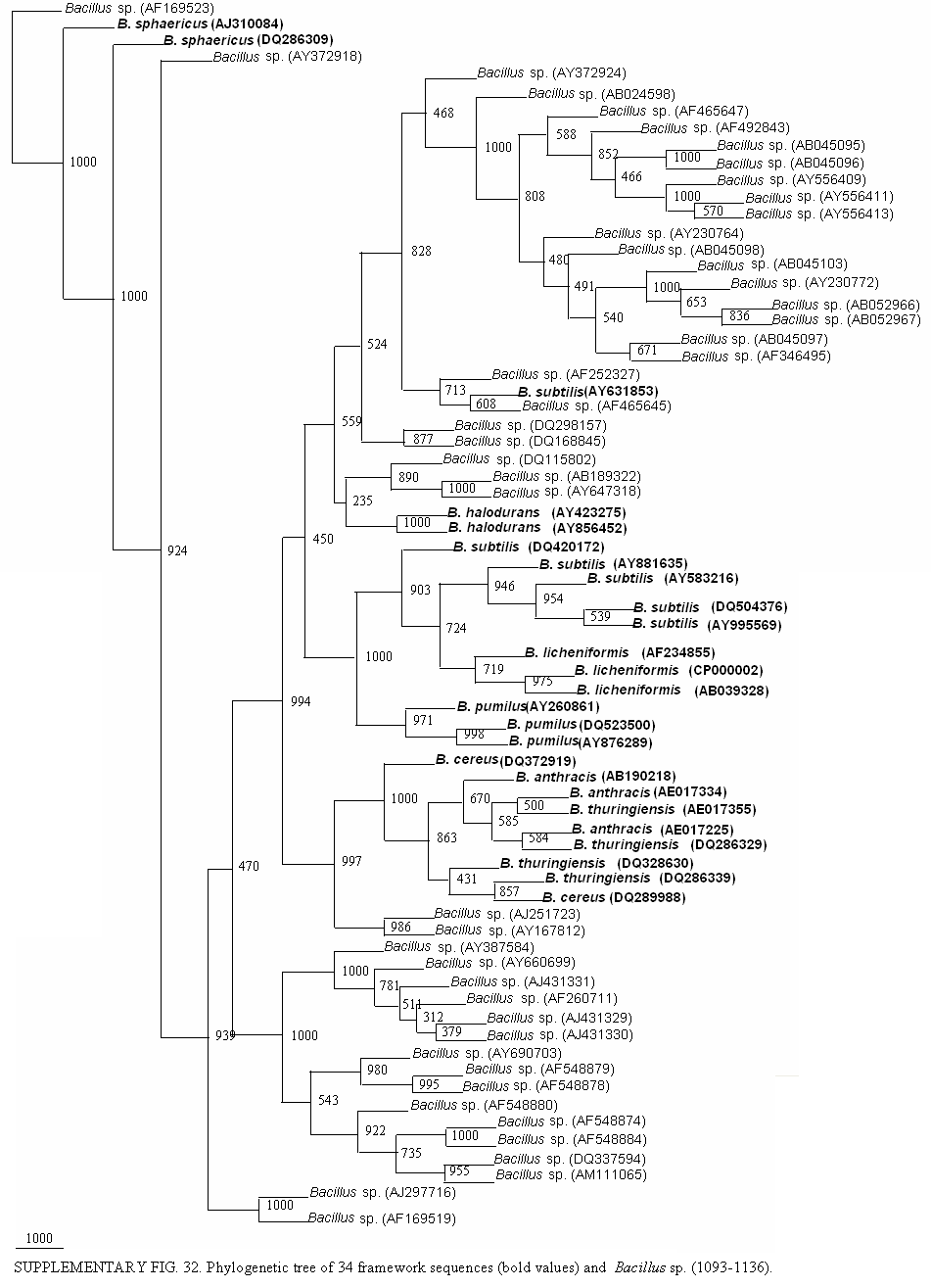

Supplement: Figure S32 — Phylogenetic tree of 34 framework sequences (bold values) and Bacillus sp. at the rate of 52 sequences (1–1136 {including 1025 Bacillus sp. and rest other species to add authenticity to the results}) which could be designated as 10 known Bacillus spp. used in the study. A neighbor-joining analysis with Jukes-Cantor correction and bootstrap support was performed on the gene sequences. Bootstrap values are given at nodes. Values in parentheses are accession numbers (http://rdp.cme.msu.edu/). (0.73 MB TIF) [file pone.0004438.s037.tif]
